# Control of resistance against bacteriophage killing by a metabolic regulator in meningitis-associated *Escherichia coli*

**DOI:** 10.1073/pnas.2210299119

**Published:** 2022-11-02

**Authors:** James P. R. Connolly, Natasha C. A. Turner, Ester Serrano, Patricia T. Rimbi, Douglas F. Browning, Nicky O’Boyle, Andrew J. Roe

**Affiliations:** ^a^Newcastle University Biosciences Institute, Newcastle University, Newcastle-upon-Tyne, NE2 4HH, United Kingdom;; ^b^Institute of Infection, Immunity and Inflammation, University of Glasgow, Glasgow, G12 8TA, United Kingdom;; ^c^College of Health & Life Sciences, Aston University, Birmingham, B4 7ET, United Kingdom;; ^d^School of Microbiology, University College Cork, National University of Ireland, Cork, T12 K8AF, Ireland

**Keywords:** regulation, *E*. *coli*, bacteriophage, transcription factor, capsule

## Abstract

Ecologically beneficial traits in bacteria are encoded by intrinsic and horizontally acquired genes. However, such traits are not universal, and the highly mosaic nature of bacterial genomes requires control at the transcriptional level to drive these processes. It has emerged that regulatory flexibility is widespread in the *Escherichia coli* species, whereby preexisting transcription factors can acquire new and unrelated roles in regulating beneficial traits. DsdC is the regulator of D-serine tolerance in *E*. *coli*, is essential for D-serine catabolism, and is often encoded by two copies in neonatal meningitis–associated *E*. *coli* (NMEC). Here, we reveal that DsdC is a global regulator of transcription in NMEC and does not require D-serine for the control of novel beneficial traits. We show that DsdC binds the chromosome in an unusual manner, with many binding sites arranged in clusters spanning entire operons and within gene coding sequences, such as *neuO*. Importantly, we identify *neuO* as the most significantly down-regulated gene in a strain deleted for both *dsdC* copies, in both the presence and absence of D-serine. NeuO is prophage encoded in several NMEC K1 isolates and mediates capsule *O*-acetylation but has no effect on attachment to or invasion of human brain endothelial cells. Instead, we demonstrate that NeuO provides resistance against K1 bacteriophage attack and that this critical function is regulated by DsdC. This work highlights how a horizontally acquired enzyme that functions in cell-surface modulation can be controlled by an intrinsic regulator to provide a key ecological benefit to an *E*. *coli* pathotype.

## Significance

Bacterial genomes are often subject to extensive horizontal gene transfer. Genes must be controlled at several levels of regulation to ensure their encoding functions are expressed appropriately and offer beneficial traits. Here, we reveal that an *Escherichia coli* transcription factor, historically linked to a narrow-spectrum metabolic pathway, interacts with the chromosome globally and by binding to DNA in an unusual fashion. These interactions affect regulation of several distinct processes, including biogenesis and modification of capsular polysaccharide. Importantly, the latter function occurs independently of the canonical metabolic inducer of this regulator and results in enhanced resistance to killing by bacteriophages that target the capsule. This work highlights that specific regulators can be reprogrammed to perform far-removed, yet critical, functions in bacteria.

Tight regulatory control of gene expression is critical for the fitness and success of bacterial pathogens that colonize multiple distinct host niches. Response systems that sense the characteristics of different environments enable bacteria to rapidly adapt the expression of their genes. Transcriptional control is mediated by many distinct components, including transcription factors (TFs) (1). TFs act by directing the activity of RNA polymerase to target genes, often in response to specific environmental cues such as metabolites (2, 3). The gram-negative bacterial species *Escherichia coli* encodes ∼300 TFs in its genome, many of which directly control the intrinsic properties of the cell (e.g., metabolism) in tandem with acquired functions such as virulence (4, 5). This link between virulence regulation and host metabolism has emerged as a key determinant of bacterial pathogenesis by dictating the niche specificity of such organisms (6). However, the genetic mechanisms that drive the capacity of pathogens to infect multiple distinct ecological niches are not fully understood.

Different *E*. *coli* pathotypes use unique virulence factors to cause infection (7). The expression of these virulence factors is often responsive to the environment and occurs through transcriptional regulation of their encoding genes. We previously discovered that the host metabolite D-serine (D-ser) is a key player in this process, selectively affecting global gene expression in a pathotype-specific manner (8–10). The gut-restricted pathotype enterohemorrhagic *E*. *coli* (EHEC) is a foodborne pathogen that colonizes the large intestine of humans using a pathogenicity island–encoded type 3 secretion system (T3SS) (7). We found that exposure to D-ser induced a shift in the expression of hundreds of genes in EHEC, most strongly defined by the repression of their T3SS and activation of an SOS-like stress response (8). The toxic effects of D-ser have a negative impact on cellular physiology through its accumulation in the cell and bacteriostatic inhibition of growth (11). D-ser accumulation can be overcome by its catabolism and use as a nutrient source (12). This is mediated by a three-gene locus encoding DsdX (an inner membrane D-ser transporter), DsdA (a deaminase that converts D-ser to ammonia and pyruvate), and DsdC (a LysR-type transcriptional regulator of the system), the latter of which is essential for functionality by sensing cellular D-ser levels and up-regulating its catabolism through direct binding to the *dsdXA* operator (12, 13). EHEC has acquired a large truncation in the *dsdCXA* locus (deletion of *dsdCX* and a portion of *dsdA*), explaining its toxic effect on this pathotype. Indeed, an analysis of its carriage across >1,500 *E*. *coli* genomes revealed that there was an equal likelihood of an isolate encoding *dsdCXA* or not. However, strains encoding the T3SS were significantly more likely to lose *dsdCXA*, with cocarriage of both loci being extremely rare (1.6%). In contrast, uropathogenic *E*. *coli* (UPEC) isolates, a pathotype that causes infection of the bladder and kidneys, widely encode an intact *dsdCXA* (>85% of isolates) and are capable of using D-ser as a sole energy source (8). Intriguingly, neonatal meningitis–associated *E*. *coli* (NMEC), a pathotype capable of crossing the blood–brain barrier, has been reported to carry two copies of *dsdCXA* (14). This observation is striking because the brain is regionally abundant in D-ser, where it functions as a neurotransmitter by activating *N-*methyl-D-aspartate receptors (15). Importantly, the relevance of this genetic scenario mirrors the biochemical characteristics of each pathotype’s respective niche. D-ser concentrations in the gut are extremely low, favoring EHEC expansion, whereas D-ser is abundant in urine and the brain, explaining the restriction of EHEC to the gut and the necessity for UPEC/NMEC to retain *dsdCXA* (8). This suggests that there exists an evolutionary pressure to either lose or retain *dsdCXA*, depending on the pathotype status of the isolate. However, the reason for carriage of two complete copies of the *dsdCXA* locus in NMEC was a mystery.

In addition to the ability to catabolize D-ser, we recently observed that the transcriptional response of UPEC and NMEC (strains CFT073 and CE10) to D-ser exposure was highly unique, displaying distinct pleiotropic shifts in global gene expression (16). This led us to hypothesize that D-ser may also be able to regulate gene expression in a specific manner that benefits the lifestyle of individual pathotypes. The recycling of existing TFs to perform new and diverse regulatory roles has emerged as an important theme in bacterial adaptation to challenging environments (2). While there is clearly a diversity in the transcriptional response of multiple *E*. *coli* pathotypes to D-ser, the role that DsdC plays in control of gene expression is unknown. Because DsdC is the classical D-ser response regulator and encoded by two copies in NMEC, we hypothesized that this TF may play a role in global transcriptional adaptation to D-ser beyond that of *dsdCXA.*

Using a combination of genetics and functional genomics, we found that DsdC binds to hundreds of chromosomal regions in NMEC and regulates several distinct virulence factors in response to D-ser, while also controlling key genes irrespective of the presence of D-ser. We identified *neuO*, encoding an *O*-acetyltransferase of the K1 capsule, as the most highly down-regulated gene in a mutant lacking both DsdC copies (17). DsdC regulates *neuO* expression in a unique manner by binding within the gene coding sequence, independently of D-ser. Deletion of *neuO* or *dsdC* had no effect on the ability of NMEC to attach to or invade brain epithelial cells; however, the mutants displayed enhanced killing by a K1-specific bacteriophage. This suggests that a key role for DsdC in NMEC is to directly control a specific bacteriophage defense system, expanding the ecological context and function of this critical TF.

## Results

### DsdC1 and DsdC2 Are Functionally Redundant in Activation of D-ser Catabolism.

**Fig. 1.**
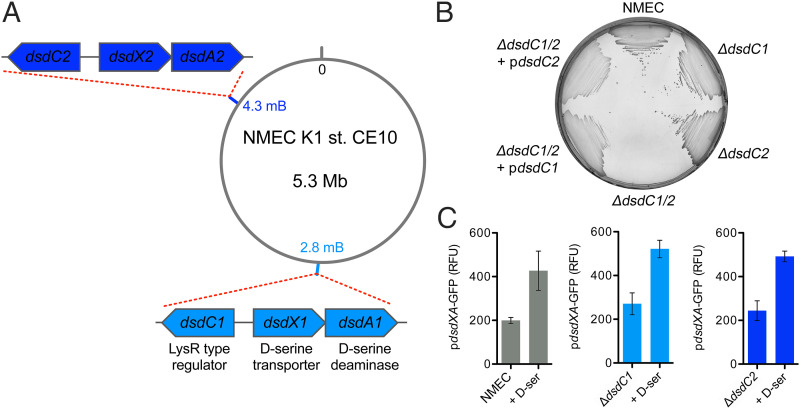
NMEC strain CE10 possesses two functional copies of *dsdCXA*. (*A*) Illustration of the chromosomal location of *dsdCXA1* (light blue) and *dsdCXA2* (dark blue) in NMEC. Details of the associated function of each gene are identical between each locus. (*B*) Growth of NMEC and *dsdC* mutant derivatives streaked onto M9 minimal medium agar plates supplemented with 10 mM D-ser as a sole carbon source. (*C*) Transcriptional reporter assays of NMEC and *dsdC* mutant derivatives carrying a *dsdXA* promoter–green fluorescent protein (GFP) fusion plasmid. Data are depicted as relative fluorescence units (RFUs; GFP/optical density). Reporter assays were performed in biological triplicate, and error bars represent SD.

The NMEC K1 strain CE10 carries two unlinked copies of the *dsdCXA* locus, located at 2.8 and 4.3 Mb along the 5.3-Mb chromosome. (Fig. 1*A*) (18). Sequence alignment of the two DsdC copies (designated DsdC1 and DsdC2) showed high conservation, with 98% homology across 98% of the coding sequence. There were four amino acid substitutions occurring in a cluster within the substrate binding domain, as well as five substitutions in the C-terminal coding sequence (*SI Appendix*, Fig. S1). To test the functionality of NMEC DsdC in D-ser catabolism, we deleted both *dsdC1* and *dsdC2* individually and together in the same background (Δ*dsdC1*/*2*). Growth of both Δ*dsdC1* and Δ*dsdC2* on MOPS minimal medium plates containing 10 mM D-ser as a sole carbon source was comparable to that of the wild type (WT) (Fig. 1*B*). This result was mirrored in an experiment using a *dsdXA* promoter–green fluorescent protein fusion as a reporter for activation of D-ser catabolism. WT NMEC, Δ*dsdC1*, and Δ*dsdC2* displayed identical activation levels of *dsdXA* transcription in response to D-ser, indicating equal functionality of both regulator copies (Fig. 1*C*). Conversely, a Δ*dsdC1*/*2* double mutant was unable to grow on D-ser as a sole carbon source (Fig. 1*B*), but expression of either *dsdC1* or *dsdC2* in trans was able to fully restore the growth defect of Δ*dsdC1*/*2.* Collectively, these data indicate that DsdC1 and DsdC2 have functional redundancy in the context of D-ser catabolism.

### DsdC1 and DsdC2 Bind Globally to the NMEC Chromosome in Two Distinct Patterns.

*E*. *coli* TFs often adapt their functionality to regulate diverse gene sets beyond their primary role (2). However, it was unknown if a role for DsdC existed beyond D-ser catabolism in NMEC, which formed a key question of this study. We first validated the ability of both DsdC1 and DsdC2 to directly bind their respective *dsdCX* promoter regions in vitro. Electrophoretic mobility shift assays (EMSAs) using purified DsdC1 and DsdC2 confirmed that both TF copies were able to bind to their respective promoter regions, but not a control fragment of the *kanamycin* gene (*SI Appendix*, Fig. S2*A*). To increase the resolution of this interaction, DNase I footprinting revealed a region of protection within the *dsdC* and *dsdXA* intergenic regions for both copies of DsdC (*SI Appendix*, Fig. S2*B*). The protected site was 47 bp in length and is identical in sequence between both the *dsdC1* and *dsdC2* promoter regions (*SI Appendix*, Fig. S2*C*). LysR-type transcriptional regulators typically bind as dimers to two 13-bp sites in close proximity upstream of regulated genes, which may explain the size of this protected region (13). Addition of D-ser to the reaction resulted in a reduction of binding to the latter protected site, suggesting D-ser can influence the strength of DsdC binding to target promoters. These data confirmed that both copies of DsdC directly and specifically bind to target DNA, regulating their respective *dsdCXA* loci in a similar fashion in NMEC.

**Fig. 2.**
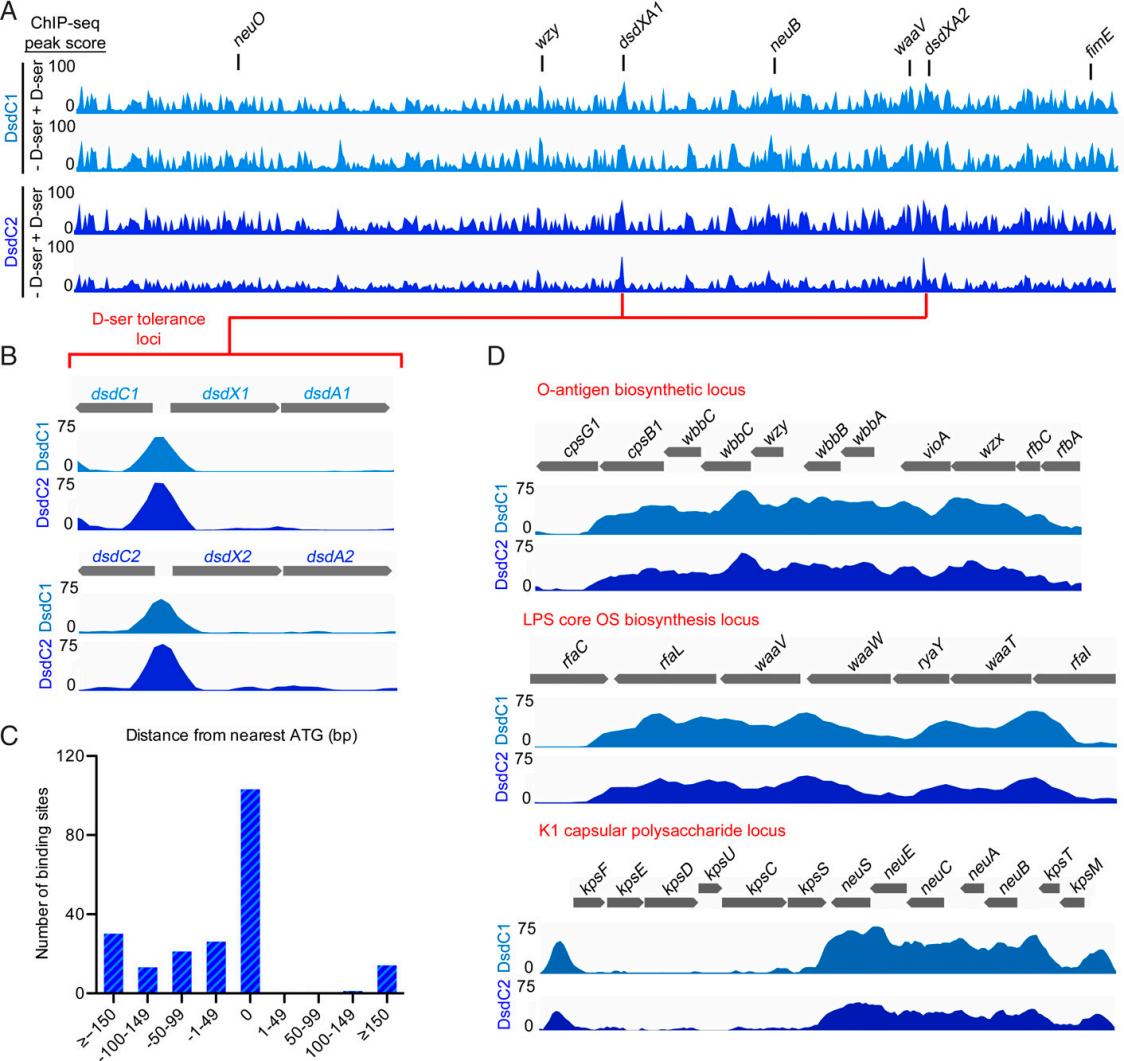
DsdC binds to the NMEC chromosome globally in distinct site-specific patterns. (*A*) Genome-wide binding dynamics of DsdC1^FLAG^ (light blue) and DsdC2^FLAG^ (dark blue) identified by ChIP-seq. Experiments were performed in the presence and absence of D-ser in the medium, indicated to the *Left* of the coverage tracks. Peaks associated with genes of interest (*P <* 0.01; two biological replicates) and their chromosomal position are highlighted above the track graphs. (*B*) Expanded view of the *dsdCXA1/2* loci and their associated DsdC ChIP-seq peaks. (*C*) Genomic context of DsdC binding sites relative to the nearest 5′ gene end clustered in 50-bp bins. (*D*) Expanded view of the O-antigen, LPS core, and K1 capsular biosynthetic loci for the ChIP-seq experiments. Binding of DsdC1/2 can be seen as noncanonical clusters of peaks that span the operons entirely. OS, oligosaccharide.

Given the broad transcriptional responses of distinct *E*. *coli* pathotypes to D-ser and the fact that NMEC encodes two functional copies of DsdC, we hypothesized that this TF may play a wider role in gene regulation (16, 18). To address this, we used chromatin immunoprecipitation sequencing (ChIP-seq) to map all binding sites of DsdC1 and DsdC2 along the NMEC genome in vivo. We first genetically engineered two NMEC strains, where either DsdC1 or DsdC2 was tagged with a 3× FLAG epitope at the C terminus to allow native expression of each regulator fusion. The DsdC1^FLAG^ and DsdC2^FLAG^ strains were then grown in M9 minimal media spiked with 1 mM D-ser at mid exponential phase (hours 3–5 in the growth curve) to assess if the presence of the FLAG-tag fusions had an impact on gross physiology. Growth was identical to that of the WT under these conditions (*SI Appendix*, Fig. S3 *A* and *B*). In parallel, we analyzed samples from these growth assays by whole-cell Western blot to confirm that the DsdC1^FLAG^ and DsdC2^FLAG^ fusions were indeed being expressed. Note that addition of D-ser only resulted in a modest increase in DsdC1^FLAG^ and DsdC2^FLAG^ expression (*SI Appendix*, Fig. S3*C*). ChIP-seq analysis of these strains, grown in the presence and absence of a D-ser spike-in, revealed an unexpected pattern of DsdC binding on a genome-wide scale (Fig. 2*A*). DsdC1^FLAG^ and DsdC2^FLAG^ bound to 95 and 140 sites, respectively, in the absence of D-ser, and 217 and 177 sites, respectively, in the presence of D-ser (*P* ≤ 0.01) (*SI Appendix*, Tables S1–S4). This suggests that D-ser likely influences which genes are targeted by DsdC, but also that DsdC may act as a global regulator of diverse NMEC processes, regardless of whether D-ser is present or not. To validate the interactions, we tested binding of DsdC to one of the novel identified regions (the *waaV* promoter region) by EMSA, confirming the interaction in vitro (*SI Appendix*, Fig. S4). A key question from this global analysis was whether or not DsdC1 or DsdC2 were capable of regulating unique gene sets. Importantly, closer inspection of the read-mapping data revealed that the DsdC1/2-specific ChIP-seq peaks identified in the peak calling pipeline were indeed present in both strains but that some peaks did not meet statistical cutoff during the data processing. This indicated that the function of both DsdC copies is likely redundant, and therefore, for the remainder of the study, we focused on regulation by DsdC1 and DsdC2 as a common process. Gene ontology (GO) analysis of open reading frames located nearest ChIP-seq peaks revealed a diverse functional range of potential targets for DsdC regulation (*SI Appendix*, Fig. S5*A*). The main functional groups of genes associated with DsdC binding sites were metabolism, transport and membrane associated, regulation, and virulence associated. The latter group is of potentially critical importance for understanding NMEC virulence regulation and included genes involved in lipopolysaccharide (LPS) and capsule biosynthesis, fimbrial adhesion, and a putative T3SS (*SI Appendix*, Fig. S5*B*) (18, 19, 20). This result suggested that DsdC is potentially a key regulator of many cellular processes in NMEC and not exclusively a regulator of D-ser catabolism.

During the analysis, we noticed that only some binding sites corresponded to the characteristic peak shape typically expected of TF binding events (an intergenic peak upstream of a gene 5′ end with a bimodal read distribution) (21). This canonical TF binding is exemplified by DsdC at the *dsdXA* promoter region, which displays a discrete peak upstream of the target genes (Fig. 2*B*). Analysis of the peak location with respect to the nearest 5′ gene boundary for all DsdC binding sites revealed that ∼50% of these sites were located close to genes, with the remainder located much farther up- or downstream of genes (Fig. 2*C*). By interrogating the distribution of signal enrichment at the latter regions, we observed an unusual pattern of DsdC binding defined by clusters of intergenic and intragenic peaks appearing in close succession and spanning entire operons (Fig. 2*D*). Importantly, these regions include operons encoding genes for key NMEC virulence–associated processes such as O-antigen, core LPS, and K1 capsular polysaccharide biosynthesis. Collectively, these data reveal that DsdC is a global regulator in NMEC, encoded by two functionally redundant copies, and binds target genes in two highly distinct manners.

### DsdC Regulates the Expression of Capsular and LPS Biosynthesis Genes.

**Fig. 3.**
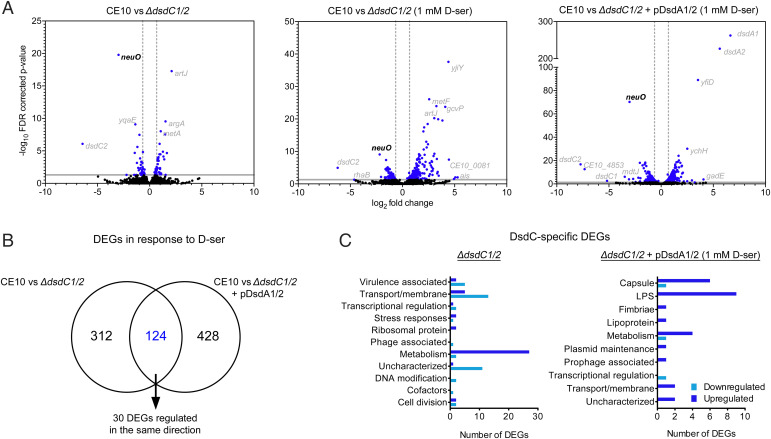
DsdC regulates diverse NMEC genes in the presence and absence of D-ser. (*A*) Volcano plots of RNA-seq analysis displaying the pattern of gene expression for the dsdC1/2 mutant relative to WT NMEC in the absence of D-ser (*Left Graph*), and the Δ*dsdC1*/*2* mutant or Δ*dsdC1*/*2* plus pDsdA complemented strain relative to WT NMEC in the presence of D-ser (*Middle* and *Right Graphs*). Significantly DEGs (false-discovery rate [FDR]–corrected *P* < 0.05) are highlighted in blue, with the gray boundaries representing the cutoff for up- or down-regulated genes. The *neuO* gene is highlighted in black because it was the most significantly down-regulated gene identified across all three comparisons. (*B*) Venn diagram summarizing the overlap in DEGs identified in the Δ*dsdC1*/*2* and Δ*dsdC1*/*2* plus pDsdA strains in response to D-ser. Thirty of these overlapping genes showed the same direction (either up- or down-regulated) of regulation. (*C*) GO analysis of DsdC-specific DEGs that were identified as being related to D-ser accumulation (Δ*dsdC1*/*2*) or dependent on D-ser exposure (Δ*dsdC1*/*2* and Δ*dsdC1*/*2* plus pDsdA).

To determine if the unusual global binding pattern of DsdC identified by ChIP-seq resulted in functional changes to NMEC gene expression, we used comparative RNA-seq analysis. The experimental design consisted of three pairwise transcriptomic comparisons (*SI Appendix*, Fig. S6) to determine DsdC-associated differentially expressed genes (DEGs; false-discovery rate *P* ≤ 0.05 and absolute fold change >1.5). We began by reanalyzing previously published data from our group (that documented the response of NMEC and Δ*dsdC1*/*2* to D-ser) comparing WT NMEC with Δ*dsdC1*/*2* grown in M9 minimal media alone to identify DsdC-regulated genes in the absence of D-ser (16). Next, we tested whether D-ser could alter the regulatory function of DsdC by comparing RNA from WT NMEC and Δ*dsdC1*/*2* cells exposed to D-ser during the exponential phase of growth (culture in M9 for 3 h followed by addition of 1 mM D-ser for 2 h). Eighty-two significant DEGs were identified comparing the WT and Δ*dsdC1*/*2* grown without D-ser, whereas in the presence of D-ser, the Δ*dsdC1*/*2* mutant displayed 436 DEGs (Fig. 3*A* and *SI Appendix*, Tables S5 and S6). Because the cellular accumulation of D-ser in the Δ*dsdC1*/*2* background would be expected to cause shifts in global gene expression associated with D-ser toxicity and not necessarily a lack of DsdC regulatory effects, a third comparison was performed to mitigate this (8). This comprised WT NMEC and Δ*dsdC1*/*2* complemented with a plasmid expressing DsdA in trans (pDsdA) under the same conditions of D-ser exposure. The logic behind this experimental design was to determine the DEGs associated with DsdC that were dependent on the presence of D-ser (i.e., sensing) but, in principle, not caused by the toxic effects of D-ser accumulation in Δ*dsdC1*/*2* because of the expression of D-ser deaminase. This comparison revealed 552 DEGs in the Δ*dsdC1*/*2* pDsdA strain after D-ser exposure (*SI Appendix*, Table S7). To narrow down the specificity of DEGs to DsdC and not the effects of D-ser accumulation, we identified 30 common DEGs regulated in the same direction (i.e., displayed up- or down-regulation) in the mutant and complement comparisons (Fig. 3*B*). This allowed us to identify a core set of DEGs that were specific to DsdC, regardless of whether D-ser was catabolized or accumulated within cells (*SI Appendix*, Table S8). The most striking result from this analysis was the identification of *neuO*, encoding the *O*-acetyltransferase of the K1 capsule, as the most significantly down-regulated gene in all three comparisons (−7.9-fold, *P* = 1.66 × 10^−20^; −4.57-fold, *P* = 8.56 × 10^−10^; and −8.15-fold, *P* = 5.07 × 10^−71^, respectively) (17). This finding suggests that there exist key DsdC-regulated genes in NMEC that are not dependent on a physiological response to D-ser.

We next combined functional grouping of DEGs with the ChIP-seq data set to identify potentially important processes regulated by DsdC. GO analysis of the common DsdC-associated DEGs in response to D-ser revealed that more than half of these were involved in capsular (7/30) and LPS (9/30) biosynthesis (Fig. 3*C*). Given the large number of DEGs identified by RNA-seq, we decided to focus on genes directly regulated by DsdC, irrespective of D-ser exposure, because these likely represent adapted functions that may be specific to NMEC biology. Intriguingly, there was very little overlap between the ChIP-seq and RNA-seq data sets. Indeed, in the absence of D-ser, only three genes were found to show differential expression in the Δ*dsdC1*/*2* background and be directly bound by DsdC. These included *neuO*, *ypdI* (an uncharacterized lipoprotein; −2.79-fold, *P* = 0.05), and *gadB* (glutamate decarboxylase; 1.5-fold, *P* = 0.04). From the 30 common DEGs that overlapped between Δ*dsdC1*/*2* and the complemented strain in the presence of D-ser, it was found that only nine were bound by DsdC. Interestingly, all of these DEGs were involved in LPS and K1 capsule biosynthesis. Taken together, these data suggest that DsdC acts as a global regulator of gene expression in NMEC, directly controlling physiologically relevant functions such as the biogenesis of cell envelope structures.

### *O*-acetyltransferase NeuO Mediates Protection Against K1 Bacteriophage Attack.

**Fig. 4.**
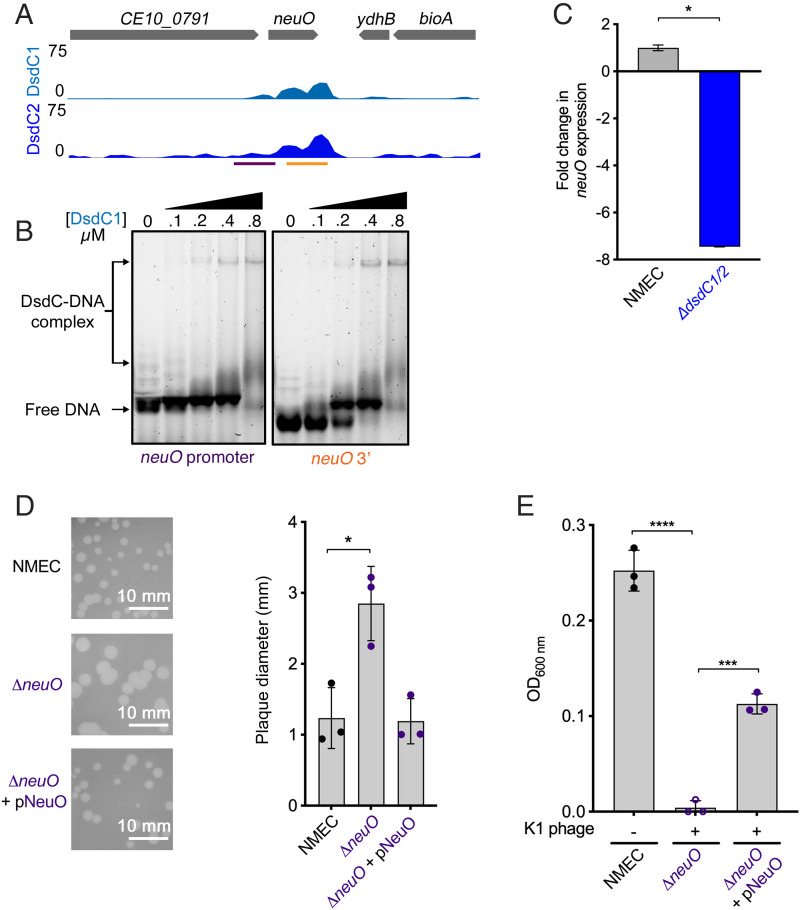
The DsdC-regulated *O*-acetyltransferase NeuO controls NMEC resistance to K1 bacteriophage attack. (*A*) Expanded view of ChIP-seq tracks showing binding of DsdC1/2 within the coding sequence of *neuO*. The peak regions selected for EMSA are illustrated below the tracks. (*B*) EMSA verifying the ability of purified DsdC to bind to the promoter region (highlighted in purple) and 3′ region (highlighted in orange) of the *neuO* coding sequence in vitro. (*C*) qRT-PCR analysis *neuO* transcript levels in the NMEC and Δ*dsdC1*/*2* backgrounds. (*D*) Phage plaque assays showing the diameter (mm) of plaques formed on NMEC-, Δ*neuO*-, or Δ*neuO* plus pNeuO–containing agar plates spotted with K1 bacteriophage–containing buffer. Experiments were performed in biological triplicate, with representative images from each assay indicated to the *Left* of the graph. (*E*) K1 bacteriophage killing assays performed in liquid media for NMEC, Δ*neuO*, or Δ*neuO* plus pNeuO. Data points were determined by measuring optical density (600 nm) after 1 h of phage exposure. *** and **** indicate *P* < 0.001 and 0.0001 respectively from three biological replicates (Student’s *t* test), with SD. *P < 0.05, ***P < 0.001, and ****P < 0.0001 derived from three biological replicates (Student *t* test) with SD.

The combined transcriptomic analyses revealed that *neuO* was the most significantly down-regulated gene as a result *dsdC1* and *dsdC2* deletion, regardless of exposure to D-ser. NeuO is an *O*-acetyltransferase responsible for *O*-acetylation of the K1 capsule and is regulated by a spontaneous phase-variation mechanism, involving slipped-strand mispairing of a heptanucleotide repeat region within the gene termed polyΨ (17). Unusually, ChIP-seq identified highly significant, noncanonical binding of DsdC1^FLAG^ (*P* = 1.98 × 10^−106^) and DsdC2^FLAG^ (*P =* 2.5 × 10^−74^) both within the coding sequence and directly downstream of the *neuO* gene, with only a very modest peak being identified within its promoter region (Fig. 4*A*). To verify these interactions, we confirmed that DsdC was capable of binding to the promoter region and a fragment corresponding to the *neuO* 3′ coding region in vitro, while in vivo binding seemed to be enriched at the intragenic sites (Fig. 4*B*). As another negative control (in addition to the *kanamycin* gene), we used a fragment of the native *adhE* gene, which was not associated with a ChIP-seq peak, to verify that this was a specific DsdC-*neuO* interaction (*SI Appendix*, Fig. S7). Additionally, we used qRT-PCR to verify that *neuO* was indeed down-regulated (more than sevenfold; *P <* 0.05) in the Δ*dsdC1*/*2* mutant background (Fig. 4*C*). These data suggest that the noncanonical binding of DsdC within the *neuO* gene region somehow regulates its expression at the transcriptional level.

We next turned our attention to what the biological role of this regulatory event might be. Adhesion and invasion of the blood–brain barrier are key virulence mechanisms of NMEC, and the K1 capsule is believed to play a role in this process (7, 19, 20, 22, 23). We therefore tested the ability of WT NMEC and a Δ*neuO* mutant to adhere to and invade the human brain microvascular endothelial cell line, hCDMEC/D3. We first confirmed that deletion of *neuO* had no observable growth defect that would have an impact on interpretation of the cell infection assays (*SI Appendix*, Fig. S8). We then determined that Δ*neuO* did not have a decreased ability to adhere to hCDMEC/D3 cells (∼20–30% adherence efficiency comparable to that of WT NMEC). Furthermore, the invasion efficiency was comparable for both WT and Δ*neuO* (∼0.002%), also displaying no statistically significant difference. In addition, we tested the ability of Δ*dsdC1*/*2* to adhere to and invade hCDMEC/D3 cells (*SI Appendix*, Fig. S9). As expected, we observed no phenotype in the Δ*dsdC1*/*2* background, suggesting that neither NeuO nor its regulator, DsdC, was involved in the ability of NMEC to attach to or invade human brain endothelial cells in vitro.

The original report describing the discovery of NeuO in NMEC strain RS218 documented that *neuO* was encoded on the CUS-3 prophage (17, 24–26). Interestingly, CUS-3 is integrated into the genome at a known hypervariable region between *dsdC* and the *argW* transfer RNA (tRNA) gene in RS218. Many K1-specific bacteriophages use the polysialic capsule as their receptor. Prophages often encode enzymes that alter their host receptor, thereby modulating superinfection by additional bacteriophages. Given the role of NeuO in *O*-acetylation of the K1 capsule, we hypothesized that deletion of *neuO* would therefore affect the ability of a K1 bacteriophage to infect and lyse NMEC CE10 (17, 27). To test this, we assayed the formation of plaques on bacterial agar plates overlaid with a mixture of NMEC-containing agar and spotted with a K1-specific bacteriophage. We observed that the average plaque diameter formed was significantly larger (∼3.8 mm; *P* = 0.0146) in the Δ*neuO* mutant background compared with WT NMEC (∼1.15 mm) (Fig. 4*D*). We confirmed that this phenotype was attributed to loss of *neuO* by complementing the mutant in trans, successfully restoring WT plaque size. To further quantify the bacteriophage killing, we monitored the levels of growth in minimal medium cultures of NMEC exposed to K1 phage. This assay revealed complete lysis of Δ*neuO* occurred after 1 h of growth, but only partial killing of the WT was observed, suggesting the ability of NMEC to resist phage attack naturally (Fig. 4*E*). We were able to partially complement this phenotype by expression of *neuO* in trans, significantly reducing the lytic burden of K1 phage exposure by ∼50% (*P* < 0.0001). Collectively, these results reveal that NeuO plays a significant role in bacteriophage defense by NMEC, likely through a mechanism involving *O*-acetylation of the K1 capsule.

### DsdC Regulates NeuO-Associated Resistance to K1 Bacteriophage Killing.

**Fig. 5.**
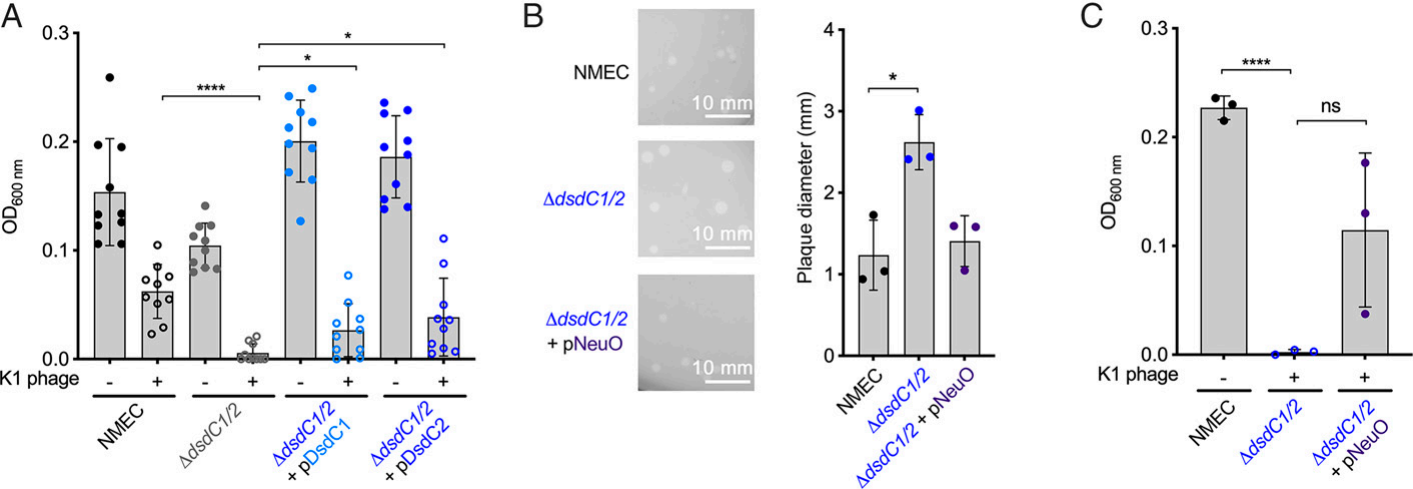
DsdC regulates NeuO*-*associated resistance to K1 bacteriophage killing. (*A*) K1 bacteriophage killing assays performed in liquid media for NMEC, Δ*dsdC1*/*2*, Δ*dsdC1*/*2* plus pDsdC1, or Δ*dsdC1*/*2* plus pDsdC2. Data points were determined by measuring optical density (OD; 600 nm) after 1 h of phage exposure. (*B*) Phage plaque assays showing the diameter (mm) of plaques formed on NMEC-, Δ*dsdC1*/*2*-, and Δ*dsdC1/2* plus pNeuO–containing agar plates spotted with K1 bacteriophage–containing buffer. Experiments were performed in biological triplicate, with representative images from each assay indicated to the *Left* of the graph. (*C*) K1 bacteriophage killing assays performed for NMEC, Δ*dsdC1*/*2*, and Δ*dsdC1*/*2* plus pNeuO. ns, not significant. *P < 0.05 (Student’s *t* test) with SD (*A*) from 10 biological replicates, ****P < 0.0001 (Student *t* test) with SD from (*A*) 10 or (*C*) three biological replicates.

To assess if DsdC played a role in resistance against bacteriophage attack via its regulatory effect on *neuO*, we tested the Δ*dsdC1*/*2* mutant in K1 killing assays. The Δ*dsdC1*/*2* mutant was significantly (*P* < 0.0001) more susceptible to bacteriophage killing than WT NMEC, mirroring the phenotype observed for Δ*neuO* (Fig. 5*A*). To confirm the functional redundancy of both copies of DsdC in regulation of this process, we successfully complemented the double mutant with both *dsdC1* and *dsdC2*. Plasmid-borne expression of DsdC1 and DsdC2 significantly (*P* = 0.0109 and *P =* 0.0045, respectively) reduced the lytic burden of K1 bacteriophage in Δ*dsdC1*/*2* by ∼50% (Fig. 5*A* and *SI Appendix*, Fig. S10). Additionally, the Δ*dsdC1*/*2* double mutant formed significantly larger plaques on bacterial soft agar plates (average 3.65 mm; *P* = 0.0117) than WT NMEC after K1 bacteriophage exposure (Fig. 5*B*). Finally, to confirm that the K1 resistance phenotype was attributable to DsdC-mediated regulation of NeuO, we also complemented the Δ*dsdC1*/*2* mutant indirectly by constitutive expression of *neuO* in trans. This bypassed the requirement for DsdC1/2 and completely restored the WT plaque size, alleviating the lytic burden of K1 phage on the Δ*dsdC1*/*2* mutant (Fig. 5 *B* and *C*). Collectively, these results confirm that NeuO is a key enzyme that plays a role in defense against bacteriophage killing through modulating the K1 capsule and that maximal protection efficiency is facilitated through regulation by the core genome–encoded TF DsdC.

## Discussion

The regulation of gene expression dictates how bacteria control cellular processes central to their survival in specific environments. Bacteria are capable of responding to their surroundings in a precise manner to express the right genes at the right time (1–3). Furthermore, the process of metabolism is often intrinsically linked to other functions, including virulence, to maximize the competitiveness of pathogens at distinct host niches (6). This is particularly important within organisms that have highly mosaic genomes, composed of both core and horizontally acquired genetic elements. Bacteria therefore require TFs to control multiple, overlapping roles in the cell through genetic regulatory adaptation (5). Here, we identify a distinct role for the regulator of D-ser catabolism in the NMEC strain CE10. DsdC controls resistance against bacteriophage attack through direct regulation of the *O*-acetyltransferase encoding gene *neuO*, representing a key regulatory adaptation contributing to the ecological success of NMEC.

NMEC strains typically encode two copies of the *dsdCXA* locus (14). This suggests that metabolism of D-ser represents an ecological advantage to NMEC, presumably offering a fitness advantage during infection of the brain. Accordingly, we demonstrate here that both loci are functionally redundant in D-ser use. However, this represents an unusual scenario, given that homologous genes with identical functions are often not stably maintained within bacterial genomes, unless there is an advantage to the organism (28). This therefore presents three possible reasons for stably maintaining both copies of the locus: double the capacity to uptake and catabolise D-ser, discrete control in two chromosomal contexts, or regulatory functions for DsdC that exist beyond the D-ser catabolism locus. In agreement with the latter hypothesis, our global analysis of DsdC in NMEC identified that both copies of the regulator bound to hundreds of sites along the chromosome, irrespective of the presence of D-ser in the environment. Close inspection of the data revealed that DsdC1 and DsdC2 do not bind to distinct regions, suggesting they are functionally redundant in general as global regulators. Importantly, we identified binding of regions that are reported to be involved in NMEC virulence, such as LPS and capsular biosynthesis (7). Transcriptomic analyses identified that these processes are differentially regulated by DsdC in a D-ser–dependent manner. This finding is in line with our previous work describing the transcriptional effects of D-ser on EHEC and UPEC virulence (16). The regulatory effects of D-ser on virulence also extend beyond the *E*. *coli* species. For instance, D-ser has been reported to induce expression of the *ssp* lipase involved in *Staphylococcus saprophyticus* virulence, and in *Proteus mirabilis*, catabolism of D-ser caused increased fitness during polymicrobial catheter-associated urinary tract infection (29, 30). Furthermore, D-ser was shown to inhibit attachment and biofilm formation in *Staphylococcus aureus*, down-regulating key genes, including *agrA*, *sarS*, and *dltD* (31). These studies, and ours, enforce the concept that D-ser modulates virulence regulation in multiple bacterial species. Despite these findings, deletion of both *dsdC* copies resulted in no observable virulence-related phenotype in NMEC, suggesting other roles for this regulator must exist.

Another striking observation from our ChIP-seq analyses was the pattern of binding to distinct chromosomal regions. Approximately half of the DsdC-bound regions appeared to form clusters spanning entire operons, as opposed to more traditional discrete binding sites located upstream of regulated genes. While this pattern was highly unusual, noncanonical TF binding has been observed in bacteria. For example, the RutR TF in *E*. *coli* K-12 binds to mostly intragenic sites, with no apparent effects on transcription (32). Indeed, most of the DsdC-bound regions in our study were not associated with transcriptional shifts. Nonregulatory binding events could therefore represent evolutionary relics, where the TF no longer functions at this site (32). However, we did observe differential expression at the LPS and capsular biosynthetic loci, which were associated with the unusual clustered binding pattern of DsdC across operons. This may represent a mechanism of transcriptional regulation that mirrors the activity of certain nucleoid-associated proteins, such as H-NS, to bind along large spans of DNA to silence transcription (33, 34). Indeed, the line between what constitutes a TF and a nucleoid-associated protein has become increasingly blurred, with the existence of seemingly overlapping functions in transcriptional regulation (35, 36). It is therefore plausible that DsdC falls into this category, acting upon its targets both by traditional promoter interactions (e.g., at *dsdCXA*) and clustered interactions that may result in effects on local DNA structure and therefore the accessibility of other factors such as RNA polymerase.

The most intriguing discovery in this work was the DsdC-dependent regulation of resistance to K1 bacteriophage attack. There are >70 capsular antigens; however, K1 is overrepresented in NMEC, accounting for 84% of isolates obtained from infected neonates, as well as pathogenic strains of *Neisseria* and *Klebsiella* sp (20, 37). The K1 capsule is a homopolymer of α2,8-linked *Ν*-acetylneuraminic acid residues (Neu5Ac), a molecular mimic of sialic acid produced on neural cells (38). *E*. *coli* K1 strains can modify their capsular structure by *O*-acetylation of carbon 7 or 9 of the sialyl units (39). We identified that maximal expression of *neuO* (encoding *O*-acetyltransferase) was dependent upon DsdC, irrespective of the presence of D-ser. *O*-acetylation of the capsule occurs in many bacterial and fungal species, for example, playing a role in protection from the bactericidal activity of serum in *Neisseria meningitidis* (40). The genomic context of *neuO* suggests it was originally acquired on a prophage, and its regulation by DsdC therefore represents an adaptive regulatory trait in NMEC. K1 bacteriophages use the K1 capsule as an attachment site, employing tailspike proteins to cleave the Neu5Ac (27). To prevent superinfections within the host cell, prophages often encode enzymes that alter their receptor. We hypothesized that shifts in *neuO* expression may affect the ability of a K1 bacteriophage to lyse the bacterial cell. Indeed, we found that NeuO (and its regulation by DsdC) was critical for providing protection against killing by K1 bacteriophages. This activity of NeuO was not dependent on D-ser. Furthermore, neither DsdC nor NeuO appear to play roles in NMEC virulence. Finally, the unusual binding pattern of DsdC within the *neuO* coding region leaves the precise mechanism of regulation unclear. While we do observe transcriptional regulation of *neuO* by DsdC (potentially because of some weak binding within the promoter region), we can also speculate that this intragenic binding event contributes to transcriptional regulation or may somehow affect the rate of slipped-strand mispairing within the *neuO* gene, thereby affecting the phase-variable state of the coding sequence (17). Indeed, we previously identified a TF in UPEC (YhaJ) that alters phase variation of type I fimbria by binding to its invertible promoter and cryptically influencing its rate of expression (41). We anticipate many novel mechanisms of TF regulation at unusual binding sites that are associated with distinct mechanisms of genetic phase variation.

The *argW* tRNA locus is a well-documented hotspot for genetic recombination in *E*. *coli* (14). For example, the acquisition of a sucrose catabolism locus in the EHEC strain EDL933 and related T3SS-encoding isolates has led to the loss of DsdC, resulting in an inability to tolerate D-ser (8). Interestingly, this site is also a common region for insertion of the *neuO*-carrying CUS-3–like phage observed in NMEC strain RS218 (24). As a result, *neuO* is commonly encoded ∼40 Kb from *dsdC*. This CUS-3–like phage appears prone to decay, because in many isolates, including RS218, genes essential for phage replication, including those encoding tail proteins and coat proteins, are annotated as pseudogenes. Moreover, there are several examples of strains exhibiting loss of genetic content in this region such that the proximity of *neuO* to *dsdC* is reduced, often to as little as 8 Kb (e.g., strain CE10 and SCU-175; accession No. CP054379.1) (*SI Appendix*, Table S9). A second, less common insertion site for CUS-3–like phage and therefore *neuO* exists between *pgl* and *bioA*. The size of this insertion also varies between 20 and 50 Kb, indicating that genetic decay may occur following acquisition. Interestingly, CE10 possesses phage-like elements in both locations, with the *argW* insertion appearing extensively degraded, including truncation of the 5′ extremity (nucleotides 537–777 remain) of *neuO*. The second *neuO* allele is, however, intact and as shown here functional in protection against K1 phage attack. Therefore, although genetic decay of CUS-3–like phages is common in *E*. *coli*, the retention of functional *neuO* alleles by the prototype NMEC isolates RS218 and CE10 indicates that capsule *O*-acetylation may provide a pathotype-specific ecological advantage through prevention of phage infection.

### Conclusion.

Tailoring transcriptional regulatory networks by recycling TFs to control alternative and often horizontally acquired genes can lead to the emergence of ecologically beneficial traits in bacteria. However, the extent of this phenomenon and associated mechanisms driving such regulation remains a key question in bacterial genetics. We identify that the TF DsdC, responsible for tolerance to D-ser, has adapted alternative metabolism-independent roles in the regulation of NMEC gene expression. We show that DsdC unusually binds to entire operons involved in envelope biogenesis, affecting their transcription, and is critical for regulating NeuO-mediated protection against bacteriophage attack. Given its carriage on a seemingly dispensable prophage, retention and subsequent control of NeuO are crucial. As such, its regulation has been hijacked by an ancestral TF to promote its continued functionality and enhance the environmental versatility of NMEC K1 isolates in the face of bacteriophage attack.

## Materials and Methods

A complete list of all bacterial strains, plasmids, and primers used in this study can be found in *SI Appendix*, Tables S10–S12. Details of all methodology related to the experiments and analyses are included in the *SI Appendix*, *Materials and Methods*. This includes growth conditions, strain generation, cloning procedures, ChIP-seq, RNA-seq, qRT-PCR, protein overexpression and purification, EMSA, DNaseI footprinting, Western blotting, cell culture, phage killing assays, and data analysis. All raw sequencing data have been deposited in the European Nucleotide Archive under the accession Nos. RNA-seq study PRJEB36547 (ERS4281326-ERS4281334, ERS4281354-ERS4281356, ERS4281338-ERS4281340, ERS4281344-ERS4281346, and ERS4281350-ERS4281352) and ChIP-seq study PRJEB36549 (ERS4281484, ERS4281489-ERS4281494, and ERS12154679-ERS12154680).

## Supplementary Material

Supplementary File

## Data Availability

RNA-seq and ChIP-seq data have been deposited in the European Nucleotide Archive (PRJEB36547, PRJEB36549). No previously published figures or tables have been lifted directly from another source. RNA-seq data described in Fig. 4 were reanalyzed using data generated by us and obtained from a public repository, as detailed in our previous publication (16).
