# Supplementary material for: Control of resistance against bacteriophage killing by a metabolic regulator in meningitis-associated Escherichia coli
Source: Proc Natl Acad Sci U S A. 2022 Nov 2;119(45):e2210299119. doi: 10.1073/pnas.2210299119 (PMC9659370; doi:10.1073/pnas.2210299119)
Supplement: Supplementary File [file pnas.2210299119.sapp.pdf]

Supplementary Information Appendix

Control of resistance against bacteriophage killing by a metabolic regulator in meningitis-associated *Escherichia coli*

James P R Connolly, Natasha C A Turner, Ester Serrano, Patricia T Rimbi, Douglas F Browning, Nicky O'Boyle and Andrew J Roe

This PDF includes:

Material and methods

Figures S1-S10

Tables S1-S12

Supplementary references

Materials and methods

Bacterial strains and growth

All strains and plasmids used in this study are detailed in Tables S1 and S2. Overnight bacterial cultures were inoculated using a single colony and grown in 5 ml of LB (16 hours) at 37°C, 200 RPM. These cultures were then diluted 1/100 into LB, SOB or M9 minimal media the following day, until the desired OD_{600} had been reached. D-serine was used at 1 mM concentration throughout. All chemicals and antibiotics were purchased from Sigma Aldrich.

Lambda Red mediated recombineering

Mutant derivatives of NMEC strain CE10 were generated using Lambda Red recombineering (1). Briefly, PCR products of the resistance cassette from either pKD3 (chloramphenicol) or pKD4 (kanamycin) were amplified to contain 50 bp overhangs directly adjacent to the 5'/3' ends of the gene of interest. PCRs were concentrated by phenol:chloroform extraction and ethanol precipitation. CE10 was transformed with pKD46 prior to the recombination and grown at 30°C. Overnight cultures of CE10 + pKD46 were grown in LB broth containing 100 μg/ml ampicillin and used to inoculate SOB broth containing 100 μg/ml ampicillin followed by culture at 30°C. At an OD_{600} of 0.2, 10 mM arabinose was added and cultures were grown until an OD_{600} of 0.4 was reached. Cells were immediately chilled on ice for 5 minutes and harvested by chilled centrifugation at 3,500 RPM for 5 minutes. The supernatant was removed, and the pellet washed five times in 1 ml of ice-cold ddH_2O. The final pellets were suspended in ice cold ddH_2O at 100X concentration relative to the starting culture volume. 50 μl of cells was mixed with 0.5-1 μg of the PCR product and electroporated. Cells were recovered in 1 ml of SOC at 37°C for 2 hours. 500 μl of the reaction was plated out onto the appropriate antibiotic and incubated overnight at 37°C. The remaining 500 μl was left at room temperature overnight and plated the following day. Colonies were screened by PCR using check primers specific to the gene of interest to determine if successful recombinants had been generated. Positive mutants were subsequently transformed with pCP20 (ampicillin, 30°), recovered and re-streaked non-selectively at 42°C to remove the resistance cassettes, leaving a clean mutant. Clean deletions were confirmed by colony PCR.

Chromosomal tagging of DsdC1 and DsdC2 with the 3X FLAG epitope was performed using a modified Lambda Red method (2). The plasmid pDOC-F was used to amplify the FLAG tag succeeded by the pKD4-derived kanamycin cassette by PCR using primers flanked by 50 bp homologous to the sequence directly adjacent to the natural stop codon of either DsdC1 or DsdC2. The recombination was then performed and screened as above for gene deletions. The resulting strains contained an in-frame 3X FLAG tag fused to the DsdC1 or DsdC2 coding sequences.

Plasmid cloning

The DsdC1 and DsdC2 complementation plasmid were generated by standard restriction ligation cloning. The genes were amplified by PCR to contain *Hind*III (5') and *BamH*I (3') overhangs cloned into pACYC184. The NeuO complementation plasmid was generated by Gibson assembly using the NEBuilder HiFi assembly design tool. The *neuO* gene was amplified by PCR to contain 20bp overhangs complementary to the insert junction of pACYC184 and assembled into PCR-amplified linearised pACYC184 along with the *gapA* promoter to allow constitutive expression. Reactions were set up in 20 μl at a vector to insert ratio of 1:1, using 10 μl of HiFi assembly master mix (NEB). Samples were incubated at 50°C for 15 minutes. For purification of DsdC1 and DsdC2, the gene coding sequences from CE10 were amplified by PCR to contain *Nde*I (5') and *Xho*I (3') overhangs and cloned into plasmid pET28 using standard restriction ligation techniques. DNA sequences to be used for DNAseI footprinting were amplified by PCR to contain *EcoR*I (5') and *Hind*III (3') overhangs and cloned into plasmid pSR using standard restriction ligation techniques. 2 μl of the assembly or ligation mix was transformed into DH5α cells by heat shock and colonies recovered after selective plating. For all cloning reactions, restriction enzymes were purchased from New England Biolabs and T4 ligase was purchased from Invitrogen. Q5 high fidelity polymerase (New England Biolabs) was also used for all cloning strategies. All plasmid inserts were sequenced (Eurofins) to confirm no errors arose during cloning procedures.

GFP-promoter fusion transcriptional reporter assays

Reporter assays were performed as previously described (3). A plasmid based *dsdXA* promoter-GFP was used as a readout of *dsdXA* transcription (4). Reporter activity was

determined by measuring the OD_{600} of the cultures at the same phase of growth. 200 μl of culture was then added to a black microtiter plate and absolute fluorescence measured using a FLUOstar Optima plate reader (BMG Labtech). Relative fluorescence units (RFU) were then determined by dividing the absolute fluorescence values by the OD_{600} of the culture. Experiments were performed in biological triplicate.

Chromatin immunoprecipitation (ChIP)

The ChIP assays were performed as previously described (3). Duplicate NMEC $DsdC1^{FLAG}$ and $DsdC2^{FLAG}$ strains were grown in 50 mL of M9 minimal media for 3 hours at 200 RPM. Samples were split and half of them spiked with 1 mM D-serine, followed by growth for a further 2 hours. WT untagged cells were prepared identically as negative control for ChIP. Cultures were crosslinked with 1% formaldehyde for 20 minutes before the reaction was stopped by addition of 0.5 M glycine. The samples were centrifuged, washed with TBS, and suspended in FA lysis buffer (50 mM Hepes-KOH, pH 7, 150 mM NaCl, 1 mM EDTA, 1% Triton X- 100, 0.1% sodium deoxycholate, 0.1% SDS) with 4 mg/ml lysozyme and incubated at 37°C for 30 minutes. Samples were then sonicated for 2 x 15 cycle runs with 30 second on/off pulses (Bioruptor Pico; Diagenode). The samples were then centrifuged at maximum speed for 5 minutes and the chromatin (supernatant) was removed. A 20μL 'input' sample was saved at this point for ChIP-PCR validation. For immunoprecipitation of the samples, a 50% slurry of Protein A sepharose beads (GE Healthcare) in TBS was pre-blocked overnight with 1% BSA to reduce background. The blocked beads were then cleaned and suspended in a 50% slurry in TBS with 0.1% BSA. The chromatin was next added to 60 μl of blocked beads and rotated for 2 hours at 4°C. The lysate was removed by centrifugation at 4,000 RPM for 1 minute and 60 μl of fresh blocked beads was added to the lysate. 4 μl of M2 mouse-monoclonal anti-FLAG antibody (Sigma Aldrich) was added to the suspension and mixed on a rotator overnight at 4°C.

The beads were then pelleted by centrifugation at 4,000 RPM for 1 minute, the lysate removed, beads suspended in 700 μl of FA lysis buffer (150 mM NaCl) and transferred to a Spin-X column (Corning). These columns were then rotated at room temperature for 3 minutes, before centrifugation for 1 minute at 4,000 RPM, and removal of the supernatant. 2 further washes, rotations and centrifugation with FA lysis buffer (150

mM NaCl) were performed. This was followed by one wash, rotation and centrifugation with FA lysis buffer (500 mM NaCl), followed by one wash, rotation and centrifugation with ChIP wash buffer (10 mM Tris-HCl, pH 8.0, 250 mM LiCl, 1 mM EDTA, 0.5% Nonidet-P40, 0.5% sodium deoxycholate). A final wash, rotation and centrifugation in TE buffer (10 mM Tris-HCl, pH 7.5, 1 mM EDTA) was performed before columns were transferred to dolphin nosed tubes. The supernatant was incubated with 100 μl of ChIP elution buffer (50 mM Tris-HCl, pH 7.5, 10 mM EDTA, 1% SDS) at 65°C for 10 minutes. Samples were then eluted by centrifugation at 4,000 RPM for 1 min. The samples and the inputs collected earlier were then de-crosslinked by boiling for 10 minutes. The supernatants containing ChIP DNA were concentrated using phenol:chloroform extraction followed by ethanol precipitation and air-dried before resuspending in 12 μl nuclease free H_2O.

ChIP coupled with next generation sequencing (ChIP-Seq)

The ChIP samples were sent to the University of Glasgow Polyomics facility where DNA concentration was measured using a Qubit HS DNA kit (ThermoFisher Scientific). Due to the low DNA concentration, the ChIP samples were pooled together before sequencing. The ChIP-Seq libraries were then prepared using a NEBNext Ultra II DNA library prep kit for Illumina (NEB). The libraries were quantified using the Qubit HS DNA kit (ThermoFisher Scientific). The profiles and the size of the libraries were analysed on the Bioanalyser High Sensitivity DNA ChIP (Agilent). Illumina next generation sequencing was performed using an Illumina NextSeq 500 platform (75 bp length; single end).

ChIP-seq data analysis

Reads were quality assessed (minimum Phred threshold of 20) with FastQC (Babraham Bioinformatics) before importing into CLC Genomics Workbench 7 (Qiagen). Raw fastq files were aligned to the CE10 reference genomes (NCBI accession number: CP003034). The ChIP-Seq analysis tool in CLC was used, which calls peaks based on an algorithm that learns the expected characteristic shape (an intersecting bimodal peak, signifying forward and reverse strand reads aligning either side of the transcription factor binding site) of ChIP-seq peaks. Data derived from FLAG-tagged strains was aligned against the mock ChIP WT control samples to establish enrichment over background. The maximum *p*-value for calling enriched

peaks was set to ≤0.05. Peaks were called from two biological replicates, and all computationally determined peaks were manually assessed to ensure they conformed unambiguously to the expected bimodal peak shape (5). Regions that showed peaks determined from sequencing noise, ambiguous alignments or an absence of a peak in both replicates were omitted.

RNA extraction and DNase treatment

Samples were grown in M9 minimal media for 3 hours at 37°C, 200 RPM. Samples were spiked with 1mM D-serine were grown for a further 2 hours. The OD_{600} was adjusted to 1.0 and RNA extraction was carried out using the PureLink RNA Mini Kit (ThermoFisher Scientific) to the manufacturer specifications. Genomic DNA was removed using TURBO DNase (ThermoFisher Scientific) and samples tested for removal of DNA by PCR. RNA was concentrated using phenol:chloroform extraction followed by ethanol precipitation. The samples were analysed on a DS-11+ Spectrophotometer (DeNovix) and assessed for degradation using agarose gel electrophoresis.

Transcriptome profiling by RNA-Seq

Library preparation and sequencing was carried out at the University of Glasgow Polyomics facility. RNA quality was assessed by Agilent Bioanalyzer 2100. Samples were ribosomally depleted using MICROBExpress (ThermoFisher Scientific) according to the manufacturer's instructions. Sequencing libraries were prepared with the TrueSeq Stranded mRNA Library Prep kit (Illumina) according to manufacturer's instructions. Sequencing was carried out on the Illumina NextSeq 500 platform with at least 10 million 75 bp single end reads being obtained. Reads were quality assessed (minimum Phred threshold of 20) with FastQC (Babraham Bioinformatics) before importing into CLC Genomics Workbench (Qiagen) and mapping to the CE10 reference genome and plasmids respectively (NCBI accession number: CP003034, CP003035, CP003036, CP003037, CP003038) using default CLC mapping parameters. Differential expression was performed using the empirical analysis of differential expression tool implemented in CLC (EdgeR) with genes displaying absolute fold changes of ≥ or ≤ 1.5, and a false-discovery rate corrected *p*-value of ≤ 0.05 being considered as differentially expressed (6). Pairwise comparison of WT and

Δ*dsdC1/2* was conducted to identify any changes in gene expression caused by the removal of the transcription factor DsdC. Pairwise comparison of WT + D-ser and Δ*dsdC1/2* + D-ser was used to identify genes differentially expressed in the presence of D-serine. A further pairwise comparison was done with WT + D-ser and Δ*dsdC1/2* + D-ser complemented with pDsdA1/2, to identify genes regulated by DsdC in response to D-serine and not by the toxic effects of D-serine accumulation in the ΔΔ*dsdC1/2* mutant. Functional groups of differentially expressed genes was carried out using STRING.

Quantitative real time PCR (RT-qPCR)

RNA samples were extracted as detailed above and normalised to a total concentration of 10 ng/μl. The LunaScript RT SuperMix kit (NEB) was used for the cDNA synthesis of the RNA samples according to the manufacturer specification. RT-qPCR was performed on cDNA using a CFX-Connect Real-Time PCR detection system (BIORAD) and the Luna Universal qPCR Master Mix kit (NEB), to the manufacturer specification. The reactions were performed in technical duplicate and each gene that was analysed was performed in biological triplicate. All genes were normalised against a housekeeping gene, *gapA*. All primers used in RT-qPCR were checked for efficiency prior to the experiment. 5 standards were made using template cDNA of 100, 20, 4, 0.8 and 0.16 ng/μl. Primers were only used if they had efficiency between 90-110%. The data was then analysed using the CFX-Connect BIORAD software, according to the $2^{-\Delta\Delta CT}$ method (7).

Recombinant DsdC overexpression and purification

Plasmids expressing recombinant 6XHis-DsdC proteins were transformed into BL21 DE3 cells. A single colony was used to inoculate an overnight culture at 37°C with 200 RPM prior to back-diluting into 2 L fresh LB and culture until an OD_{600} of 0.5 was reached. 0.5 mM of IPTG was then added and cultures were left to grow overnight at 30°C. The cells were harvested by centrifuged for 20 minutes at 5,000 RPM and the supernatant removed. The cell pellet was resuspended in Buffer A (50 mM Tris, 0.5 M NaCl and 5 % glycerol) containing lysozyme, EDTA-free protease inhibitor cocktail (Promega) and DNase. The sample was lysed by sonication on ice, 1 second ON and 1 second OFF for 6 minutes and then centrifuged for 50 minutes at 4°C at 18,000

RPM. After centrifugation, the 6XHis-DsdC protein was found in the soluble fraction. The supernatant was removed and filtered through a 0.22 μM filter and loaded onto an Ni^{2+}-chelating column (HisTrap High Performance, GE Healthcare) equilibrated with Buffer A containing 5 mM imidazole. 6XHis-DsdC was eluted with a linear gradient from 10 to 300 mM imidazole in Buffer A. Fractions containing 6XHis-DsdC were pooled and dialyzed against Buffer A and stored at -20 °C in Buffer A. Protein concentration was determined using BCA protein assay kit (Pierce) according to the manufacturer's specifications.

Electrophoretic mobility shift assay (EMSA)

Purified recombinant DsdC1 and DsdC2 proteins was used for this experiment. DsdC DNA-binding sites identified by ChIP-seq were amplified using PCR and purified by QIAquick gel extraction (QIAGEN) for use as binding probes. Equal concentrations of the purified PCR fragments (100 nM) were incubated on ice in binding buffer (50 mM Tris HCl pH 8, 0.1 mM EDTA, 5 mM $MgCl_2$,1 mM DTT, 0.5 ug/ml poly dI-dC and 5 % glycerol) without and with increasing amounts of purified DsdC in a total volume of 16 μl. After careful mixing, samples were incubated for 30 min at 30°C, placed back on ice for 10 min, then loaded onto a 0.8 % agarose gel in 0.5X TBE. Electrophoresis was carried out in 0.5X TBE at 90 volts, 4°C. Finally, the gel was stained with Gel-Red in 0.5X TBE and imaged with UV illumination on a ChemiDoc imaging system (BioRad). EMSAs were performed in triplicate.

DNase I footprinting

DNase I footprinting was carried out as previously described (3). pSR-*dsdCX* was purified using a QIAGEN MaxiPrep with a concentration of at least 100 μg of DNA needed for DNase I footprinting. The fragments were then cut using *Hind*III and incubated for 3 hours at 37°C, before being phosphatase treated with CIP. The fragments were precipitated using a phenol:chloroform extraction followed by an ethanol precipitation. Samples were suspended in 50 μl of TE. The fragments were then further cut with *Aat*II, incubated for 3 hours at 37°C. The samples were then run on a 7.5% acrylamide gel. If the band was the appropriate size, based upon on a molecular ladder, it was cut from the gel and the DNA was electroeluted from the gel slice. Electroelution occurred by preparing dialysis tubing, inserting the gel slice into

the tubing and adding 200 μL of 0.1X TBE. These were then run for 20 minutes at 40 mA, and the supernatant kept. The fragments were precipitated using a phenol:chloroform extraction followed by an ethanol precipitation. The fragments were then radiolabelled. For each 20 μL reaction: 8 μL of DNA fragment, 8 μL of sterile H_2O, 2 μL of PNK buffer, 1 μL of ATP γ P32, and 1 μL of T4 polynucleotide kinase (NEB) was used. This was incubated at 37°C for 30 minutes. The unincorporated nucleotides were then removed by passing the samples through a Sephadex G-50 column. The filtration of the DNA fragments from the unincorporated nucleotides was done by adding 200 μL of Sephadex G-50 beads to a spin column. This was allowed to settle for 5 minutes, before centrifugation at low speed (3.5 RPM) occurred for 2 minutes. The DNA fragments were then pipetted onto the beads and centrifuged for 2 minutes (3.5 RPM). The supernatant was kept as the ^{32}P-end radiolabelled DNA fragments, and the columns disposed of appropriately. For DNase I footprinting, each 20 μL reaction was made up as follows: 0.2 μL of ^{32}P-end labelled DNA fragment, 2 μL of 10X HEPES buffer (200 mM Hepes pH 8.0, 50 mM $MgCl_2$, 500 mM potassium glutamate, 10 mM DTT), 1 μL of 10 mg/ml^{-1} BSA, 1 μL of 0.6 mg/ml^{-1} Herring sperm DNA, varying concentrations of DsdC, and H_2O to bring the volume up to 20 μL. Samples were then incubated for 20 minutes at 37°C and 2 μL of DNase I was added to the sample for 40 seconds before the reaction was stopped with 200 μL of DNase I stop solution (0.3 M sodium acetate pH 7.0, 10 mM EDTA). The samples were then precipitated using phenol-chloroform extraction followed by ethanol precipitation. The samples were suspended in loading buffer (97% v/v deionized formamide, 20 mM EDTA, 0.05% bromophenol blue, 0.05% xylene cyanol FF) and heated to 90°C for 2 minutes. The samples were loaded onto a 6% denaturing gel with a GA ladder and visualised on a Bio-Rad PMI imager.

SDS-PAGE and western blot

1 ml of bacterial culture was resuspended in 4X LDS buffer (Thermo Fisher) and boiled for 10 minutes before centrifuging to remove the cellular debris. 20 μl lysate was loaded into each well of a NuPAGE 4-12 % Bis-Tris mini gel and electrophoresed at 180 volts for 50 minutes. Proteins were transferred to 0.45 μm nitrocellulose membrane (GE Healthcare) at 30 volts for 1 hour. All methods were carried out using the Novex gel tank and XCell II blot module systems (Thermo Fisher). Membranes

were blocked for 1 hour in PBST + 5 % milk. Blocked membranes were then probed with anti-FLAG (1/5000) or anti-DnaK (1/5000) primary antibodies, followed by 1/10000 dilution of HRP-conjugated secondary antibodies (Sigma). Blots were developed using the SuperSignal West Pico kit (Pierce) and imaged using a ChemiDoc imaging system (BioRad). Western blots were performed in duplicate.

hCMEC/D3 adhesion and invasion assays

The hCMEC/D3 blood-brain barrier cell line (Merck) was cultured and maintained in ENDGRO-LS complete medium according to the manufacturers specifications (Merck). Collagen T1 Rat Tail solution (1:20, Sigma) was used to coat T75 flasks prior to seeding. Cells were cultured in vented T75 flasks (37°C, 5 % $CO2_2$) until passage 10. For splitting, 2 ml Trypsin-EDTA was added to the flask for 5 minutes before 8 ml of pre-warmed hCMEC/D3 ENDGRO-LS complete medium was added and cells removed. Cell count was determined using a haemocytometer. For adhesion and invasion assays, 24-well tissue culture plates were coated in a 0.5% (v/v) collagen solution for 1 hour prior to seeding and kept at 37°C. The wells were washed once with PBS. 40,000 cells were seeded per well and left for 24 hours to form a confluent monolayer. The CE10 strains were grown in M9 minimal media for 5 hours at 37°C, with 200 RPM, and normalised to an OD_{600} of 0.1. For an MOI of 100; 50 µl of bacteria was added to each well with 450 µl of hCMEC/D3 ENDOGro-LS Complete media. The plates were centrifuged at 200 g for 2 minutes and incubated for 2 hours at 37°C, 5% CO_2.

For the adhesion assay, cells were washed three times with 500 µl of PBS, with the wash supernatant kept to serially dilute in PBS and calculate the CFU/ml. 300 µl of 1% Triton X-100 was added to the wells and left for 5 minutes. The cells were removed from the wells and serially diluted in PBS and the CFU/ml was calculated. For the invasion assay, cells were again washed three times with 500 µl of PBS and the CFU/ml determined as above. 500 µl of hCMEC/D3 ENDOGro-LS Complete media with 100 µg/ml of gentamicin was added to the cells and left to incubate at 37°C, 5% CO_2 for a further hour. 300 µl of 1% Triton X-100 was added to the wells and left for 5 minutes. The cells were removed from the wells and serially diluted in PBS and the CFU/ml was calculated.

K1 bacteriophage titre and plaque assay

E. coli strains were grown in LB at 37°C, 200 RPM for 16 hours. The samples were then diluted 1/100 in M9 minimal media and grown until an OD_{600} of 0.6 was reached. 100 μl of bacterial culture was added to 3 ml of warm PTA agar, spread onto PB plates, and allowed to dry. Serial dilutions of K1 bacteriophage (SSI Diagnostica) were done in phage buffer and 10 μl of each dilution was spotted onto the plates in technical replicates. PFU/ml was calculated as:

$$\frac{PFU}{ml} = \frac{number\ of\ plaques}{dilution\ of\ plate\ counted\ x\ volume\ of\ lysate\ plated}$$

Plaque size was measured using ImageJ software (Fiji). All experiments were completed in triplicate. Unpaired student *t* tests were used for statistical analysis.

K1 bacteriophage killing assay

E. coli strains were grown in LB at 37°C, 200 RPM for 16 hours. The strains were diluted 1/100 in M9 minimal media in a 96 well microplate and grown for 3 hours in a humidity chamber at 37°C, 200 RPM. 1 μl of K1 phage (SSI Diagnostica) was added to the samples and the OD_{600} was measured every 30 minutes or hour for 3 hours using a FLUOstar Optima plate reader (BMG Labtech). All experiments were completed in triplicate. Unpaired Student *t* tests were used for statistical analysis.

Data analysis and software

ChIP-seq and RNA-seq analysis was performed within CLC Genomic Workbench version 7.5 (Qiagen). All other statistical analyses were carried out using GraphPad Prism version 8. Sequenced alignment of *neuO* genomic regions and genomic context visualisation was performed using EasyFig version 2.1.

A

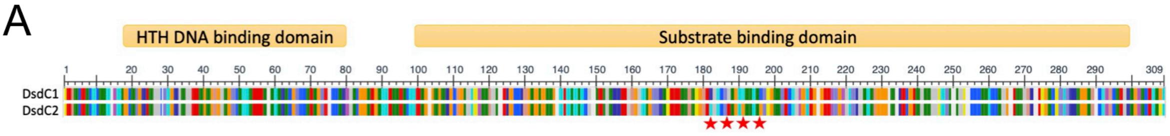

B

```
CE10_DsdC1      MEPLREIRNRLLNGWQLSKLHTFEVAARHQSFALAAEELSLSPSAVSHRINQLEEELGIQ 60
CE10_DsdC2      MEPLREIRNRLLNGWQLSKLHTFEVAARHQSFALAAEELSLSPSAVSHRINQLEEELGIQ 60
                ************************************************************

CE10_DsdC1      LFVRSHRKVELTHEGKRVYWALKSSLDTLNQEILDIKNQELSGTLTLYSRPSIAQCWLVP 120
CE10_DsdC2      LFVRSHRKVELTHEGKRVYWALKSSLDTLNQEILDIKNQELSGTLTLYSRPSIAQCWLVP 120
                ************************************************************

CE10_DsdC1      ALGDFTRRYPSISLTVLTGNDNVNLQRAGIDLAIYFDDAPSAQLTHHFLMDEEILPVCSP 180
CE10_DsdC2      ALGDFTRRYPSISLTVLTGNDNVNLQRAGIDLAIYFDDAPSAQLTHHFLMDEEILPVCSP 180
                ************************************************************

CE10_DsdC1      EYAQRHALTNQVINLRHCTLLHDRQAWSNDSGTDEWHSWAQHYAVNLPTSSGIGFDRSDL 240
CE10_DsdC2      ENAQRHDLTNTVINLSHCTLLHDRQAWSNDSGTDEWHSWAQHYAVNLPTSSGIGFDRSDL 240
                * **** *** **** ********************************************

CE10_DsdC1      AVIAAMNHIGVAMGRKRLVQKRLASGELVAPFGDMTVKCHQHYYITTLPGRQWPKIEAFI 300
CE10_DsdC2      AVIAAMNHIGVAMGRKRLVQKRLASGELVAPFGDMTVKCHQHYYITTLPGRQWPKIEAFI 300
                ************************************************************

CE10_DsdC1      TWLREQVSQYECYTL   315
CE10_DsdC2      TWLREQVCQSCQYQ-   314
                *******.*   *
```

Fig S1. Sequence comparison of DsdC1 and DsdC2 from NMEC CE10. (A) predicted functional domains according to sequence. Amino acid differences are highlighted by red stars. (B) Sequence alignment of DsdC1 and DsdC2. Amino acid changes are highlighted in grey.

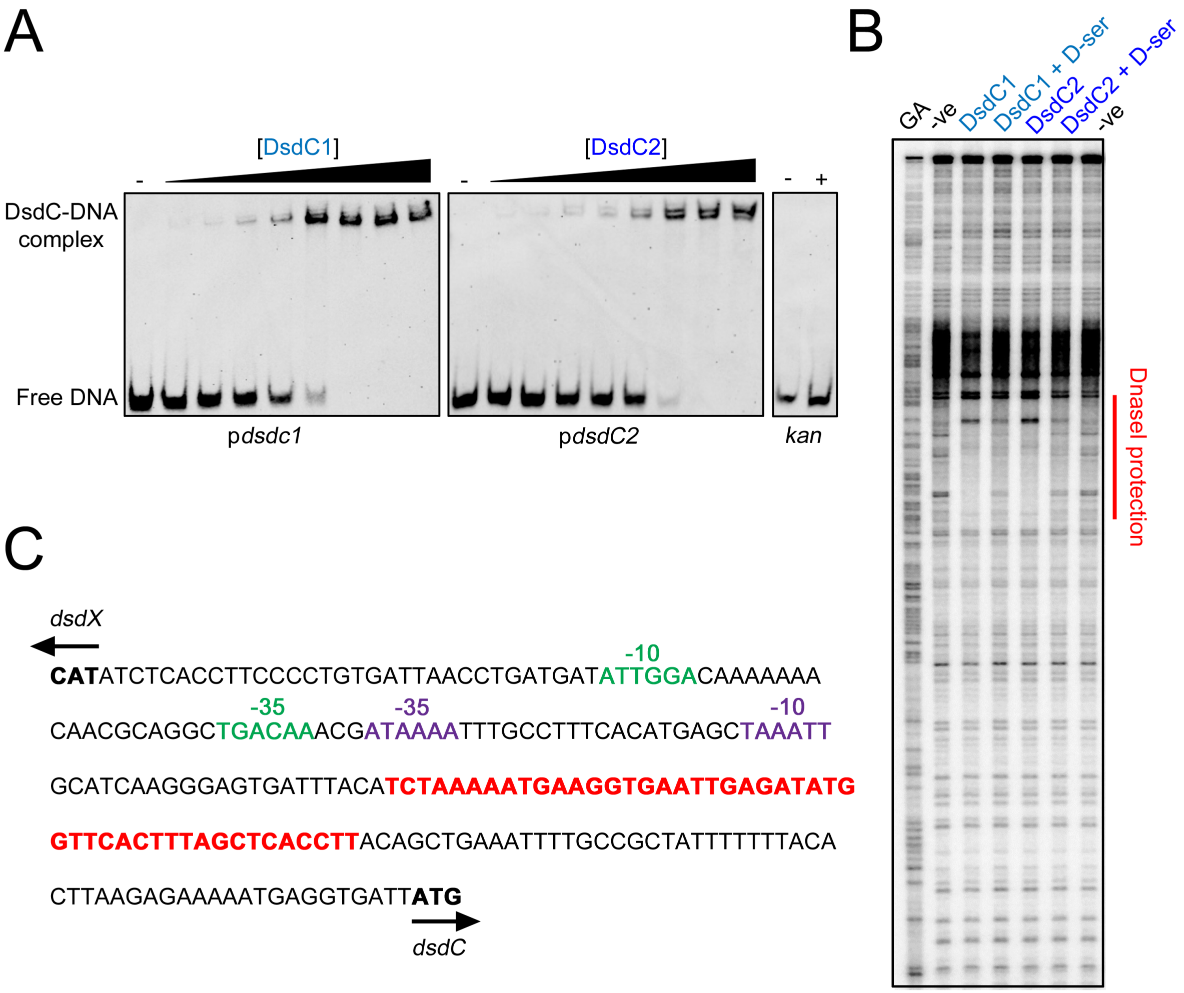

Fig S2. Validation of DsdC DNA binding properties. (A) EMSA analysis of purified DsdC1 and DsdC2 binding to the *dsdXA* promoter region. Concentrations of DsdC used range from 0 to 1.6 µM (0.2 µM increments). A fragment of the *kan* gene was used as a negative control for non-specific binding of DsdC1 (2 µM) to DNA. (B) DNAse I Footprint analysis of DsdC1 and DsdC2 binding to the *dsdXA* promoter region in the absence and presence of D-ser. The region of DNAse protection is indicated by the red block. (C) Sequence of the *dsdXA-dsdC* intergenic region. The *dsdXA* and *dsdC* promoters are highlighted in green and blue respectively. The region of DsdC binding is highlighted in red. Note, this region of protection is completely conserved between the *dsdC1* and *dsdC2* promoter regions.

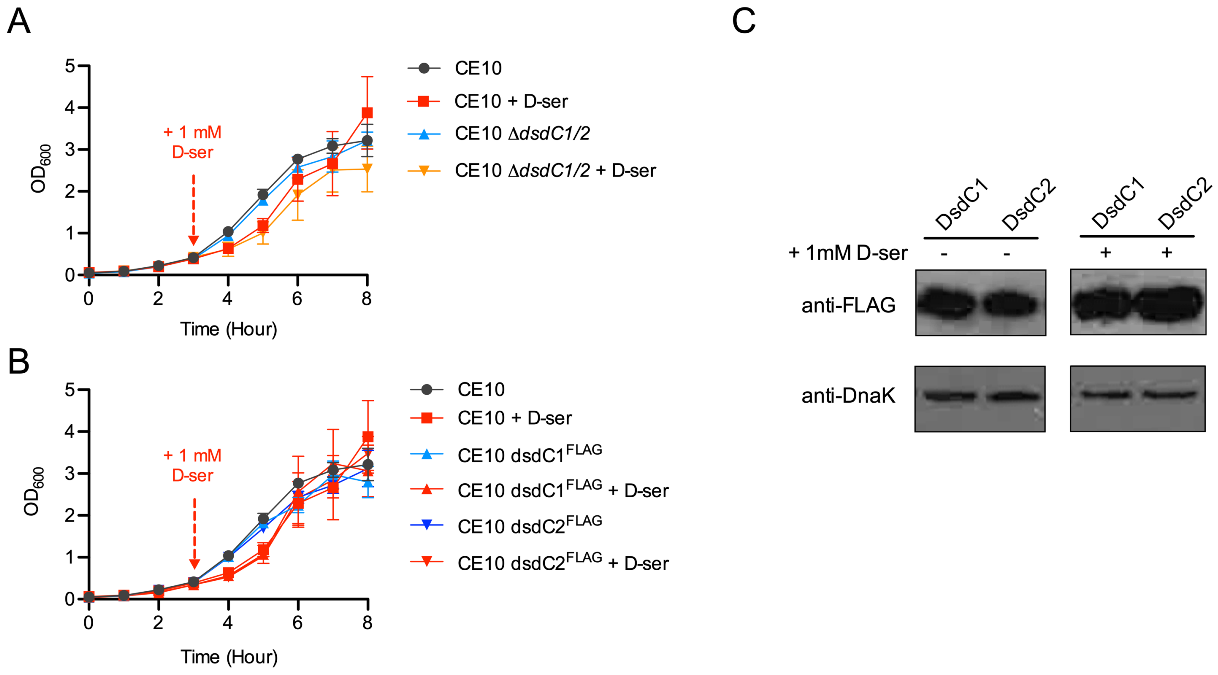

Fig S3. Physiology of DsdC-FLAG variants. (A) Growth dynamics of wild type CE10 and mutant derivatives in M9 minimal media with 1 mM D-ser spiked in after 3 hours of growth. (B) Growth dynamics of CE10 DsdC1^{FLAG} and DsdC2^{FLAG} cultured under the same conditions. (C) Western blot analysis of DsdC1^{FLAG} and DsdC2^{FLAG} expression after 5 hours of growth in M9 minimal media with (+) or without (-) D-ser spike. DnaK levels were used to determine equal loading. Growth curves and western blots were performed in biological triplicate. Error bars represent standard deviation.

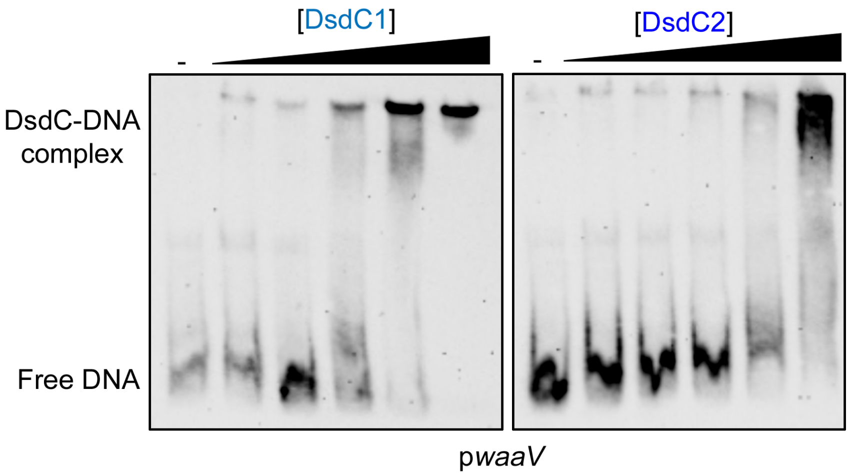

Fig S4. EMSA analysis confirming that DsdC can bind to the *waaV* promoter region. Concentrations of DsdC used range from 0 to 1.25 μM (0.25 μM increments).

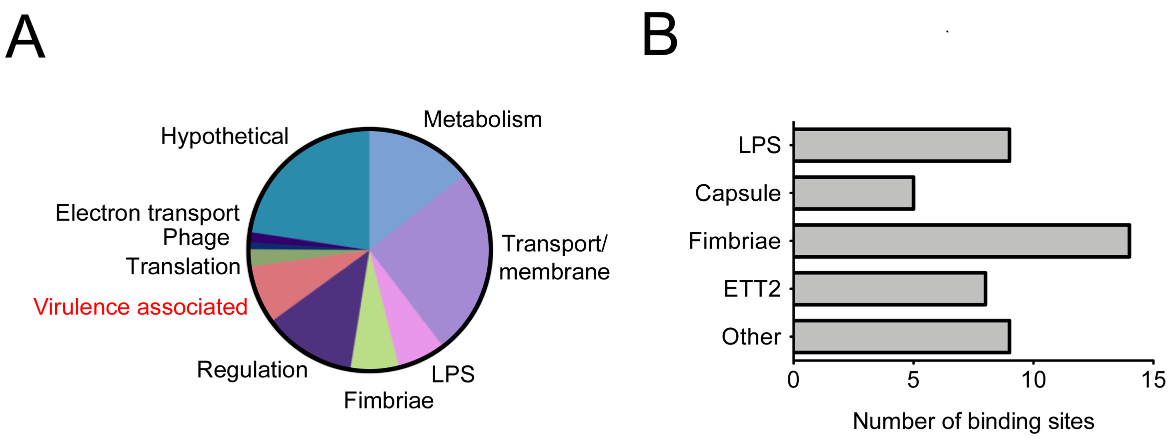

Fig S5. Functional categories of DsdC-bound gene targets. (A) Pie chart illustrating the split of GO categories identified for DsdC. (B) Genes bound by DsdC related to virulence processes broken down by subcategory.

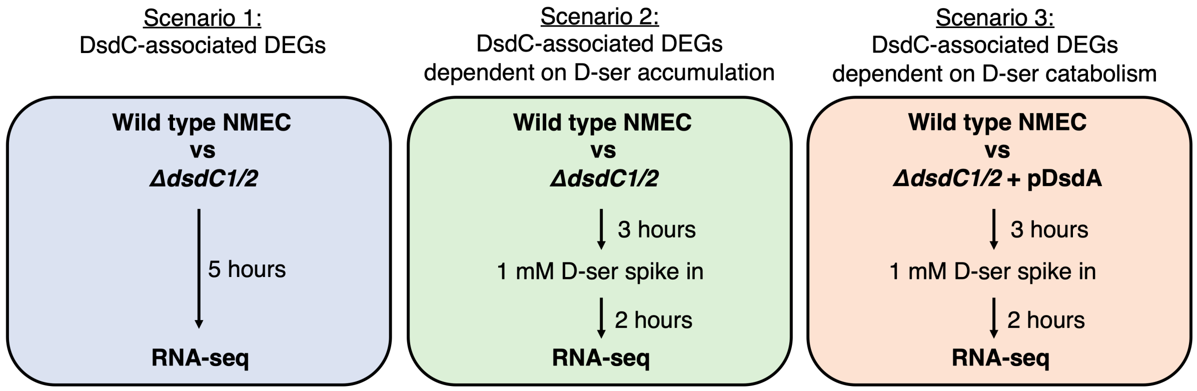

Fig S6. Experimental strategy for determining DsdC regulated genes by RNA-seq. Three scenarios were tested to determine - the DEGs associated with deletion of *dsdC1/2* in the absence of D-ser (scenario 1); deletion of *dsdC1/2* in the presence of D-ser to allow D-ser accumulation (scenario 2); and deletion of *dsdC1/2* in the presence of D-ser with pDsdA complementation to alleviate the stress response of D-ser accumulation (scenario 3).

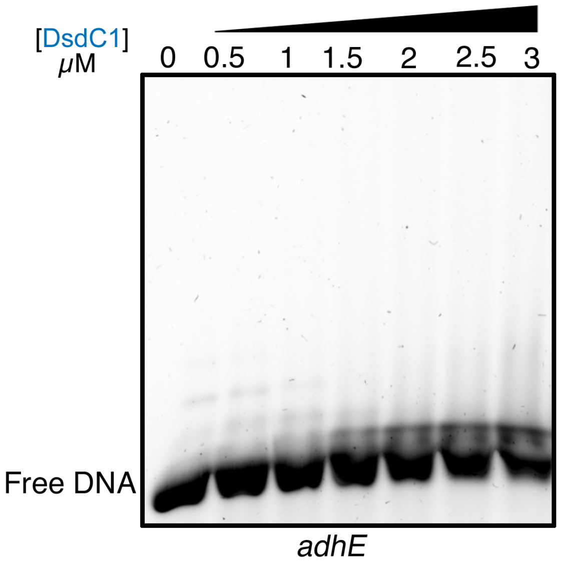

Fig S7. EMSA analysis of purified DsdC and a DNA fragment corresponding to the *adhE* gene.

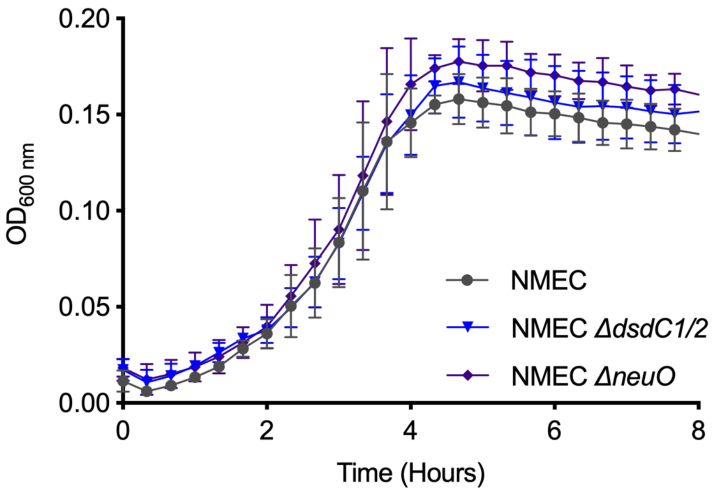

Fig S8. Growth dynamics of wild type the *ΔneuO* mutant compared to wild type NMEC and *ΔdsdC1/2*. Growth curves were performed in biological triplicate and error bars represent standard deviation.

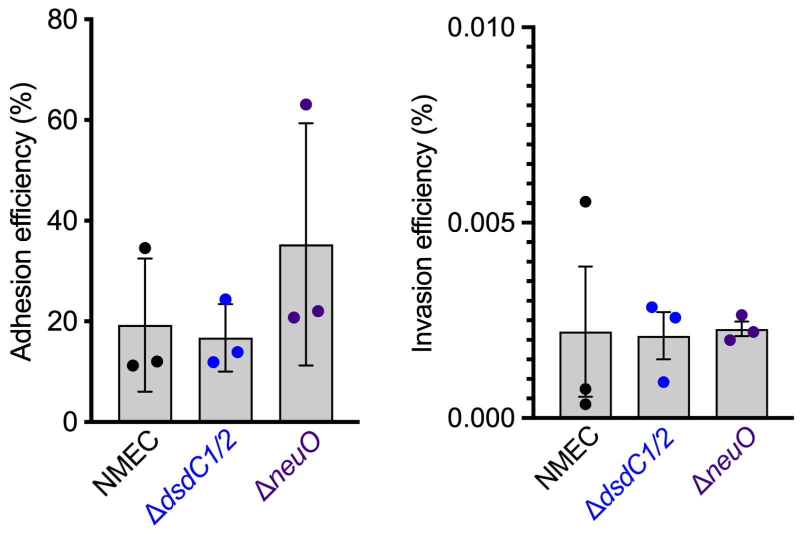

Fig S9. Efficiency of NMEC, *ΔneuO* and *ΔdsdC1/2* strains to adhere to and invade hCDMEC/D3 brain microvascular endothelial cells *in vitro.* Cell infection assays were performed in biological triplicate and error bars represent standard deviation.

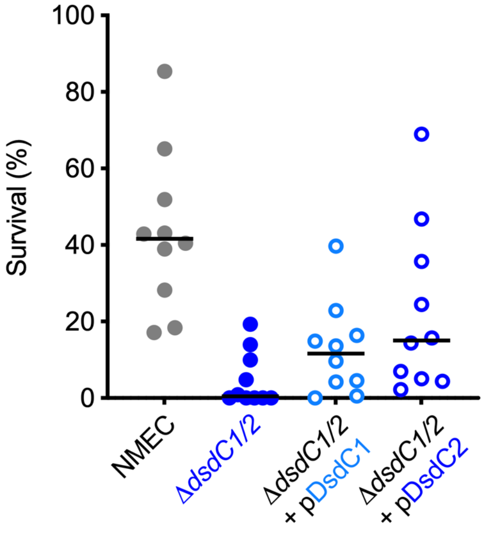

Figure S10. Survival of NMEC, *ΔdsdC1/2* and complemented strains (pDsdC1 and pDsdC2) during growth in the presence of K1 bacteriophage. Survival efficiency (%) was calculated relative to the parent strain grown without bacteriophage present. Underlying data is derived from Figure 5A. Phage killing assays were performed N=10.

Table S1. Binding sites identified by ChIP-seq for NMEC DsdC1^{FLAG} in M9 minimal media

			Replicate 1					Replicate 2				
Gene	Dist. Gene	Context	Co-ordinates	Centre	Length	Peak score	P-value	Co-ordinates	Centre	Length	Peak score	P-value
CE10_0023	-701	Intragenic	27441..27818	27655	378	52.08	0.00+00E	27570..27709	27655	140	14.01	6.54E-45
CE10_0067	0	Intragenic	79557..79975	79725	419	43.16	0.00+00E	79539..79698	79594	160	8.30	5.26E-17
yadN	0	Intergenic	166926..167348	167185	423	42.16	0.00+00E	166217..166355	166272	139	6.78	6.19E-12
tauA	-100	Intergenic	365148..365547	365378	400	42.36	0.00+00E	365288..365432	365378	145	9.85	3.50E-23
CE10_0889	0	Intergenic	940940..941277	941109	338	40.46	0.00+00E	941073..941226	941173	154	8.27	6.93E-17
ysdS	0	Intergenic	2249533..2249989	2249820	457	40.39	0.00+00E	2249802..2249931	2249877	130	8.68	1.99E-18
wbbC	0	Intragenic	2367786..2368225	2368057	440	61.35	0.00+00E	2367937..2368062	2368008	126	17.76	7.20E-71
wzy	-105	Intergenic	2368985..2369359	2369154	375	51.61	0.00+00E	2369039..2369172	2369118	134	12.62	8.65E-37
CE10_2740	4	Intergenic	2788664..2789046	2788830	383	48.83	0.00+00E	2788877..2788999	2788949	123	11.62	1.66E-31
dsdC1	0	Intergenic	2791235..2791582	2791414	348	48.25	0.00+00E	2791368..2791477	2791423	110	26.56	9.87E-156
CE10_3076	0	Intergenic	3138070..3138508	3138240	439	43.25	0.00+00E	3138181..3138283	3138234	103	10.42	9.80E-26
yqeI	-59	Intergenic	3340403..3340735	3340557	333	40.49	0.00+00E	3340524..3340674	3340624	151	11.91	4.97E-33
ygeG	-62	Intergenic	3343448..3343743	3343556	296	47.65	0.00+00E	3343481..3343606	3343533	126	13.72	3.75E-43
prgI	28	Intragenic	3350452..3350809	3350656	358	47.71	0.00+00E	3350640..3350776	3350722	137	11.01	1.71E-28
eprH	0	Intergenic	3351564..3351974	3351806	411	44.53	0.00+00E	3351640..3351757	3351705	118	12.11	4.76E-34
epaS	0	Intragenic	3354052..3354436	3354278	385	50.92	0.00+00E	3354192..3354316	3354262	125	14.23	2.86E-46
epaO	0	Intergenic	3356511..3356931	3356764	421	42.61	0.00+00E	3356785..3356857	3356814	73	9.25	1.17E-20
neuE	-169	Intragenic	3544618..3544986	3544780	369	44.39	0.00+00E	3544725..3544861	3544780	137	10.00	7.61E-24
neuA	-364	Intragenic	3547233..3547655	3547381	423	42.36	0.00+00E	3546798..3546956	3546853	159	7.46	4.32E-14
neuB	-278	Intergenic	3548187..3548660	3548503	474	52.96	0.00+00E	3548362..3548475	3548417	114	11.07	9.15E-29
slp	-296	Intragenic	4101919..4102392	4102088	474	47.18	0.00+00E	4102402..4102530	4102477	129	11.19	2.34E-29
rfaL	0	Intragenic	4258249..4258601	4258438	353	46.51	0.00+00E	4258330..4258481	4258436	152	10.51	3.80E-26
waaV	0	Intergenic	4260038..4260392	4260235	355	47.63	0.00+00E	4260181..4260334	4260235	154	11.21	1.76E-29

waaT	0	Intergenic	4262851..4263201	4263019	351	50.22	0.00+00E	4262962..4263125	4263017	164	11.49	7.34E-31
dsdC2	0	Intergenic	4332276..4332616	4332446	341	47.05	0.00+00E	4332401..4332520	4332456	120	26.13	7.67E-151
sipD	0	Intergenic	4365627..4366074	4365905	448	42.98	0.00+00E	4365896..4366048	4365996	153	8.65	2.65E-18
CE10_4297	0	Intergenic	4367298..4367739	4367571	442	42.64	0.00+00E	4367521..4367649	4367595	129	10.00	7.93E-24
espY4	0	Intragenic	4441550..4442034	4441719	485	45.46	0.00+00E	4441179..4441292	4441238	114	8.94	2.01E-19
CE10_4362	118	Intragenic	4442253..4442560	4442395	308	50.06	0.00+00E	4442079..4442190	4442136	112	11.23	1.52E-29
CE10_4466	-357	Intragenic	4553284..4553676	4553453	393	41.50	0.00+00E	4552933..4553055	4553001	123	5.97	1.16E-09
espX4	0	Intragenic	4852614..4853038	4852784	425	40.69	0.00+00E	4852729..4852847	4852784	119	10.61	1.33E-26
ubiC	-671	Intragenic	4853255..4853725	4853565	471	50.85	0.00+00E	4853445..4853536	4853500	92	11.76	3.11E-32
yjbM	0	Intragenic	4862216..4862614	4862386	399	39.72	0.00+00E	4863124..4863266	4863178	143	7.85	2.15E-15
CE10_4768	163	Intragenic	4870355..4870783	4870524	429	40.40	0.00+00E	4870388..4870551	4870443	164	10.53	3.25E-26
CE10_4943	0	Intergenic	5047562..5047946	5047777	385	43.37	0.00+00E	5047680..5047795	5047735	116	12.09	5.73E-34
fimE	-48	Intergenic	5174559..5174954	5174728	396	39.62	0.00+00E	5174609..5174720	5174663	112	5.55	1.45E-08
yjiC	0	Intergenic	5189283..5189779	5189610	497	50.71	0.00+00E	5189546..5189702	5189653	157	10.73	3.65E-27
yhhZ	0	Intragenic	4029255..4029623	4029454	369	38.34	6.40E-322	4029061..4029192	4029138	132	11.19	2.26E-29
yfaL	0	Intergenic	2651239..2651608	2651399	370	38.09	8.47E-318	2650745..2650878	2650824	134	7.10	6.18E-13
eivC	-375	Intragenic	3360277..3360778	3360445	502	37.88	2.69E-314	3360412..3360544	3360493	133	9.76	8.52E-23
hutU	0	Intergenic	769533..769906	769737	374	37.76	2.62E-312	769648..769781	769727	134	7.56	2.00E-14
dusA	-266	Intragenic	4862853..4863311	4863013	459	37.47	1.23E-307	4862252..4862408	4862306	157	11.94	3.57E-33
sipB	0	Intergenic	4362509..4363016	4362847	508	37.42	1.02E-306	4362820..4362943	4362875	124	10.36	1.88E-25
CE10_4870	0	Intergenic	4974868..4975249	4975038	382	37.18	7.45E-303	4863124..4863266	4863178	143	7.85	2.15E-15
rfaY	0	Intragenic	4261765..4262267	4261929	503	36.57	4.35E-293	4974995..4975105	4975051	111	12.89	2.72E-38
fucA	-12	Intergenic	3285996..3286441	3286164	446	36.45	3.97E-291	3286095..3286228	3286149	134	9.47	1.41E-21
leuO	-90	Intergenic	92721..93073	92888	353	36.44	5.01E-291	92834..92931	92887	98	8.09	2.96E-16
yghJ	0	Intergenic	3567445..3567945	3567615	501	35.95	3.00E-283	3567538..3567665	3567579	128	7.97	7.89E-16
cmtB	0	Intragenic	3449900..3450402	3450233	503	35.13	1.08E-270	3450052..3450182	3450106	131	7.95	9.36E-16
ydeK	-49	Intergenic	1743092..1743461	1743292	370	34.74	9.24E-265	1743217..1743352	1743298	136	8.95	1.71E-19

CE10_2939	0	Intergenic	2996411..2996816	2996581	406	34.72	2.17E-264	2996566..2996705	2996651	140	8.72	1.34E-18
envR	0	Intergenic	3874440..3874808	3874610	369	34.62	6.73E-263	3874519..3874636	3874566	118	9.32	5.87E-21
caiT	-60	Intergenic	50204..50678	50509	475	34.47	1.12E-260	50442..50571	50517	130	7.59	1.62E-14
CE10_4228	-73	Intergenic	4305007..4305391	4305176	385	34.35	7.04E-259	4305108..4305261	4305163	154	5.66	7.41E-09
sfmA	0	Intergenic	561180..561551	561349	372	33.94	9.25E-253	561286..561403	561349	118	11.53	4.50E-31
yliE	-718	Intragenic	908923..909268	909093	346	33.29	2.83E-243	909011..909154	909066	144	8.06	3.70E-16
CE10_3627	0	Intergenic	3707366..3707869	3707701	504	33.23	2.06E-242	3707646..3707779	3707736	134	7.31	1.38E-13
yhfL	0	Intergenic	3944853..3945223	3945023	371	33.08	2.62E-240	3944945..3945046	3945001	102	8.45	1.47E-17
ycbQ	0	Intergenic	1039138..1039516	1039308	379	32.73	2.65E-235	1039278..1039383	1039330	106	7.21	2.83E-13
kpsM	-233	Intragenic	3550328..3550769	3550600	442	32.58	4.32E-233	3550189..3550321	3550267	133	7.08	7.16E-13
yjbE	0	Intergenic	4839210..4839675	4839506	466	32.39	1.67E-230	4839462..4839591	4839493	130	7.87	1.79E-15
CE10_1660	299	Intragenic	1690807..1691181	1691023	375	32.19	1.10E-227	1690990..1691128	1691045	139	5.93	1.47E-09
CE10_1948	-40	Intergenic	1970983..1971368	1971152	386	31.07	3.21E-212	1971119..1971281	1971227	163	5.10	1.67E-07
yeeN	0	Intergenic	2298004..2298367	2298203	364	30.45	6.41E-204	2298099..2298252	2298203	154	6.26	1.92E-10
CE10_4567	-60	Intergenic	4657507..4657867	4657703	361	30.42	1.60E-203	4657648..4657784	4657701	137	8.59	4.46E-18
CE10_4598	0	Intergenic	4685814..4686203	4686034	390	30.16	4.41E-200	4685991..4686151	4686046	161	9.29	7.97E-21
setC	-19	Intergenic	4358033..4358399	4358240	367	30.05	9.56E-199	4358168..4358290	4358216	123	7.42	6.06E-14
CE10_0066	0	Intergenic	78738..79150	78986	413	29.77	4.80E-195	78834..78948	78896	115	8.25	7.89E-17
yiiG	0	Intergenic	4651369..4651762	4651539	394	29.68	7.58E-194	4651497..4651615	4651561	119	10.66	7.52E-27
csiD	-303	Intragenic	3140284..3140638	3140454	355	28.92	3.30E-184	3140404..3140518	3140458	115	9.31	6.38E-21
stpA	0	Intergenic	3150387..3150730	3150557	344	28.90	5.20E-184	3150497..3150637	3150551	141	7.01	1.18E-12
CE10_1661	0	Intergenic	1692605..1692994	1692825	390	28.71	1.37E-181	1692767..1692883	1692821	117	7.69	7.50E-15
yiaW	0	Intergenic	4217589..4217945	4217759	357	27.47	2.10E-166	4217675..4217805	4217751	131	6.83	4.27E-12
ecpD	0	Intergenic	166102..166449	166272	348	27.40	1.22E-165	166217..166355	166272	139	6.78	6.19E-12
fimB	0	Intergenic	5173511..5173987	5173681	477	27.32	1.33E-164	5173596..5173706	5173649	111	3.98	3.52E-05
speF	-187	Intergenic	730553..731056	730887	504	27.30	1.88E-164	730743..730811	730784	69	6.69	1.12E-11
CE10_5175	999	Intragenic	5280007..5280442	5280177	436	27.20	3.62E-163	5280928..5281088	5281034	161	10.63	1.08E-26

fucP	0	Intragenic	3286583..3286944	3286776	362	27.12	2.65E-162	3286722..3286845	3286777	124	9.88	2.52E-23
yiaY	0	Intergenic	4222357..4222771	4222602	415	26.65	9.83E-157	4222623..4222735	4222681	113	7.12	5.49E-13
fdrA	0	Intergenic	549560..550008	549839	449	26.57	7.72E-156	549686..549819	549741	134	7.96	8.66E-16
yqiK	0	Intergenic	3647776..3648120	3647951	345	26.52	3.20E-155	3647855..3647992	3647938	138	6.27	1.81E-10
ais	0	Intergenic	2677305..2677651	2677475	347	26.20	1.35E-151	2677414..2677528	2677464	115	8.07	3.48E-16
yfbL	0	Intergenic	2697398..2697781	2697568	384	26.15	5.01E-151	2697489..2697584	2697547	96	6.69	1.13E-11
ypdI	0	Intergenic	2808139..2808484	2808306	346	25.83	2.05E-147	2808225..2808359	2808279	135	5.66	7.36E-09
nanC	0	Intergenic	5172391..5172767	5172598	377	25.55	2.72E-144	5172451..5172570	5172517	120	6.82	4.46E-12
rpmE2	-210	Intergenic	281778..282115	281951	338	25.42	6.69E-143	281850..282013	281905	164	5.58	1.19E-08
yeiT	0	Intergenic	2539340..2539736	2539510	397	24.89	4.77E-137	2539468..2539591	2539520	124	7.12	5.37E-13
yobF	-202	Intergenic	2126724..2127140	2126973	417	24.75	1.52E-135	2126925..2127080	2127028	156	6.90	2.51E-12
yahL	0	Intergenic	326755..327210	326924	456	23.97	2.96E-127	326904..327028	326959	125	6.48	4.71E-11
CE10_0335	-24	Intergenic	370032..370485	370193	454	23.38	3.63E-121	370106..370265	370211	160	5.88	2.08E-09
CE10_3982	0	Intragenic	4045074..4045400	4045219	327	23.32	1.26E-120	4045141..4045246	4045203	106	6.61	1.91E-11
agaB	0	Intergenic	3746884..3747262	3747100	379	22.13	9.08E-109	3747000..3747149	3747095	150	8.06	3.80E-16
neuO	59	Intragenic	839634..840140	839803	507	21.88	1.98E-106	839932..840043	839987	112	4.9	4.87E-07
ycbR	0	Intergenic	1039827..1040283	1039995	457	21.57	1.61E-103	1039972..1040091	1040027	120	8.63	3.18E-18
yjeJ	0	Intergenic	4993586..4993924	4993757	339	21.38	1.01E-101	4993712..4993820	4993766	109	6.43	6.35E-11

Table S2. Binding sites identified by ChIP-seq for NMEC DsdC1^{FLAG} in M9 minimal media plus 1 mM D-ser

			Replicate 1					Replicate 2				
**Gene**	**Dist. Gene**	**Context**	**Co-ordinates**	**Centre**	**Length**	**Peak score**	**P-value**	**Co-ordinates**	**Centre**	**Length**	**Peak score**	**P-value**
CE10_0023	-671	Intragenic	27499..27746	27649	248	49.93	0.00E+00	27439..27831	27664	393	23.93	7.89E-127
tauA	-160	Intergenic	365260..365487	365354	228	40.45	0.00E+00	365165..365532	365363	368	21.02	2.09E-98
wbbC	0	Intragenic	2367910..2368130	2368008	221	49.57	0.00E+00	2367780..2368239	2368070	460	25.27	3.24E-141
CE10_2740	-203	Intergenic	2788863..2789052	2788948	190	40.38	0.00E+00	2789978..2790338	2790146	361	21.21	3.88E-100
dsdC1	-5	Intergenic	2791325..2791520	2791423	196	56.72	0.00E+00	2791243..2791582	2791413	340	29.86	3.73E-196
CE10_2939	0	Intergenic	2996531..2996749	2996652	219	41.56	0.00E+00	2996439..2996793	2996624	355	18.57	2.76E-77
ygeG	0	Intergenic	3343554..3343783	3343652	230	40.55	0.00E+00	3342998..3343347	3343186	350	21.19	5.89E-100
CE10_3282	0	Intergenic	3347661..3347860	3347759	200	42.75	0.00E+00	3347572..3348074	3347740	503	23.89	1.92E-126
eprH	-55	Intergenic	3351683..3351896	3351799	214	42.47	0.00E+00	3351566..3351932	3351763	367	19.89	2.50E-88
neuB	-378	Intergenic	3548287..3548559	3548384	273	40.41	0.00E+00	3548221..3548619	3548480	399	22.40	1.97E-111
yhhZ	0	Intergenic	4029031..4029235	4029138	205	42.58	0.00E+00	4029334..4029732	4029497	399	18.58	2.42E-77
yiaW	0	Intergenic	4217648..4217872	4217775	225	38.65	0.00E+00	4217597..4217952	4217784	356	13.54	4.65E-42
CE10_4160	0	Intragenic	4233642..4233911	4233814	270	42.50	0.00E+00	4233461..4233943	4233774	483	20.89	3.25E-97
rfaL	615	Intragenic	4258299..4258534	4258437	236	49.48	0.00E+00	4258248..4258605	4258436	358	22.80	2.53E-115
waaV	0	Intergenic	4260105..4260377	4260280	273	43.30	0.00E+00	4260039..4260378	4260209	340	21.19	6.41E-100
waaT	0	Intergenic	4262918..4263149	4263016	232	43.09	0.00E+00	4262799..4263173	4263006	375	23.42	1.34E-121
CE10_4229	0	Intergenic	4308317..4308519	4308422	203	41.02	0.00E+00	4308239..4308607	4308409	369	17.98	1.28E-72
dsdC2	0	Intergenic	4332349..4332573	4332447	225	54.90	0.00E+00	4332272..4332621	4332452	350	30.23	5.07E-201
CE10_4270	0	Intragenic	4347338..4347592	4347497	255	45.28	0.00E+00	4347255..4347740	4347425	486	23.35	7.41E-121
sipD	0	Intergenic	4365860..4366057	4365960	198	41.21	0.00E+00	4365603..4366105	4365937	503	19.63	4.38E-86
CE10_4297	0	Intergenic	4367470..4367709	4367612	240	41.21	0.00E+00	4368027..4368395	4368231	369	18.16	5.29E-74
yjbE	-61	Intergenic	4839351..4839589	4839492	239	42.66	0.00E+00	4839261..4839654	4839430	394	18.00	9.42E-73
espX4	0	Intragenic	4852657..4852854	4852754	198	40.23	0.00E+00	4852605..4853086	4852775	482	21.07	8.16E-99

ubiC	0	Intragenic	4853401..4853639	4853499	239	45.55	0.00E+00	4853330..4853734	4853565	405	23.16	6.39E-119
yjbM	0	Intragenic	4862291..4862525	4862389	235	41.75	0.00E+00	4862208..4862551	4862375	344	20.73	8.68E-96
CE10_4768	317	Intragenic	4870344..4870630	4870442	287	38.54	0.00E+00	4870317..4870811	4870487	495	21.01	2.86E-98
CE10_4870	0	Intergenic	4974954..4975169	4975072	216	41.21	0.00E+00	4974872..4975235	4975066	364	18.33	2.33E-75
CE10_4943	0	Intergenic	5047640..5047864	5047738	225	41.43	0.00E+00	5047570..5047909	5047740	340	20.46	2.50E-93
yjiC	-179	Intergenic	5189472..5189709	5189613	238	39.77	0.00E+00	5189325..5189740	5189571	416	22.11	1.33E-108
epaS	265	Intragenic	3354076..3354339	3354242	264	38.36	3.10E-322	3354022..3354405	3354242	384	22.88	3.64E-116
wzy	-116	Intergenic	2368996..2369247	2369093	252	38.26	1.20E-320	2368912..2369293	2369124	382	22.36	5.19E-111
CE10_5175	0	Intragenic	5280860..5281131	5281034	272	38.16	6.89E-319	5280803..5281146	5280973	344	18.02	6.96E-73
lpfA	-13	Intergenic	4456513..4456771	4456674	259	38.10	6.76E-318	4456436..4456798	4456603	363	18.42	4.85E-76
wzx	0	Intergenic	2373675..2373872	2373770	198	37.97	1.03E-315	2373570..2373904	2373739	335	18.25	1.04E-74
slp	-116	Intergenic	4102360..4102572	4102475	213	37.75	4.15E-312	4101932..4102290	4102101	359	21.06	9.09E-99
envR	-40	Intergenic	3874501..3874703	3874606	203	37.68	6.27E-311	3874440..3874838	3874610	399	18.12	1.01E-73
setC	-60	Intergenic	4358138..4358358	4358235	221	37.01	4.00E-300	4358063..4358415	4358232	353	15.81	1.34E-56
sfmA	-14	Intergenic	561287..561490	561385	204	36.90	2.03E-298	561191..561547	561360	357	17.08	1.03E-65
CE10_3398	-118	Intergenic	3470862..3471063	3470966	202	36.82	4.24E-297	3470796..3471222	3470966	427	15.67	1.14E-55
CE10_4363	504	Intragenic	4445578..4445794	4445697	217	36.60	1.65E-293	4445543..4445908	4445713	366	22.23	8.52E-110
yhiM	0	Intergenic	4083276..4083495	4083398	220	36.31	4.91E-289	4083128..4083475	4083298	348	20.40	7.63E-93
ygiL	0	Intergenic	3641678..3641883	3641776	206	36.11	7.17E-286	3641600..3641975	3641769	376	20.12	2.33E-90
CE10_3627	-18	Intergenic	3707654..3707851	3707755	198	36.00	4.22E-284	3707493..3707888	3707719	396	18.11	1.23E-73
yhaI	0	Intergenic	3713998..3714219	3714096	222	35.52	1.23E-276	3713925..3714268	3714095	344	16.08	1.69E-58
eivC	-437	Intragenic	3360339..3360630	3360437	292	35.40	7.47E-275	3360325..3360830	3360495	506	16.87	3.90E-64
CE10_0067	-52	Intragenic	79557..79762	79665	206	35.28	5.62E-273	79497..79989	79666	493	22.37	3.48E-111
pitB	-45	Intergenic	3592013..3592252	3592155	240	35.27	8.54E-273	3591908..3592286	3592117	379	17.22	9.69E-67
espY4	0	Intragenic	4441633..4441849	4441731	217	35.13	1.33E-270	4439564..4439923	4439734	360	21.28	7.93E-101
yqeI	-39	Intergenic	3340559..3340755	3340657	197	35.10	2.99E-270	3340364..3340802	3340634	439	17.26	4.91E-67
torY	-12	Intergenic	2176815..2177022	2176913	208	34.73	1.36E-264	2176745..2177084	2176915	340	15.94	1.67E-57

fepE	-80	Intergenic	638504..638708	638602	205	34.59	1.92E-262	638447..638874	638617	428	11.99	2.12E-33
CE10_4312	398	Intragenic	4390373..4390601	4390505	229	34.55	7.09E-262	4390143..4390586	4390417	444	19.85	5.52E-88
yfaL	-40	Intergenic	2651310..2651511	2651408	202	34.14	7.95E-256	2651224..2651650	2651394	427	19.76	3.47E-87
prgI	-96	Intragenic	3350520..3350752	3350656	233	33.82	5.93E-251	3350469..3350830	3350638	362	21.19	5.96E-100
yhaC	716	Intragenic	3730344..3730635	3730538	292	33.63	2.88E-248	3730245..3730703	3730535	459	22.28	3.01E-110
yadN	-146	Intergenic	167088..167288	167192	201	33.28	3.31E-243	166959..167353	167184	395	19.55	1.95E-85
epaO	-41	Intergenic	3356717..3356947	3356814	231	32.85	5.27E-237	3356528..3356943	3356774	416	18.86	1.14E-79
CE10_4466	-433	Intragenic	4553360..4553551	4553453	192	32.78	5.16E-236	4553249..4553607	4553438	359	20.18	6.84E-91
yfcV	-209	Intergenic	2769906..2770124	2770029	219	32.78	5.43E-236	2769710..2770190	2770021	481	19.99	3.64E-89
speF	-416	Intergenic	730782..730985	730888	204	32.74	2.45E-235	730580..731027	730858	448	16.05	2.97E-58
rfaY	0	Intragenic	4261842..4262076	4261940	235	32.64	5.12E-234	4261768..4262126	4261938	359	18.29	5.43E-75
CE10_4598	0	Intergenic	4685949..4686161	4686047	213	32.63	8.07E-234	4685820..4686203	4686034	384	16.05	3.02E-58
garP	0	Intragenic	3735586..3735781	3735684	196	32.43	5.16E-231	3735500..3735905	3735670	406	15.24	9.92E-53
tnaA	0	Intergenic	4430400..4430614	4430498	215	32.41	9.00E-231	4430100..4430607	4430438	508	15.06	1.44E-51
yqiK	0	Intergenic	3647840..3648034	3647938	195	32.31	2.68E-229	3647709..3648091	3647922	383	13.65	9.47E-43
CE10_4228	-155	Intergenic	4305094..4305309	4305212	216	32.25	2.04E-228	4305017..4305384	4305215	368	18.05	4.03E-73
yghT	0	Intergenic	3589574..3589779	3589672	206	32.22	4.42E-228	3589494..3589850	3589681	357	14.82	5.52E-50
yiiG	0	Intergenic	4651447..4651642	4651545	196	32.20	7.59E-228	4651380..4651744	4651550	365	14.40	2.49E-47
CE10_4613	0	Intergenic	4703832..4704039	4703929	208	31.84	7.96E-223	4703697..4704049	4703880	353	15.97	9.86E-58
CE10_3508	0	Intergenic	3578743..3578967	3578870	225	31.84	1.03E-222	3578557..3579003	3578834	447	15.84	7.73E-57
ytcA	-32	Intergenic	4904288..4904497	4904384	210	31.72	4.02E-221	4904108..4904552	4904384	445	13.57	3.10E-42
kpsF	-306	Intergenic	3533457..3533707	3533614	251	31.66	2.85E-220	3533443..3533784	3533615	342	21.35	2.02E-101
cmtB	0	Intragenic	3450039..3450329	3450233	291	31.50	4.82E-218	3449910..3450401	3450233	492	18.03	6.04E-73
sipB	0	Intergenic	4362810..4363009	4362913	200	31.36	4.07E-216	4362523..4363030	4362861	508	17.34	1.28E-67
ybcK	0	Intragenic	569810..570028	569907	219	31.35	4.50E-216	569765..570178	569935	414	17.31	1.87E-67
CE10_4997	-145	Intergenic	5106630..5106836	5106727	207	31.09	1.89E-212	5106531..5106937	5106701	407	17.60	1.17E-69
yghJ	0	Intergenic	3567481..3567702	3567579	222	31.07	2.98E-212	3567439..3567947	3567609	509	15.27	6.03E-53

fdrA	-40	Intergenic	549671..549893	549796	223	30.99	4.04E-211	549602..549982	549816	381	14.61	1.17E-48
yfgH	0	Intergenic	2938103..2938308	2938211	206	30.77	3.32E-208	2938013..2938372	2938180	360	13.63	1.28E-42
espX1	0	Intergenic	28494..28696	28587	203	30.79	2.06E-208	28364..28727	28558	364	20.48	1.72E-93
ecpD	0	Intergenic	166165..166403	166306	239	30.69	4.46E-207	166136..166494	166305	359	15.07	1.29E-51
caiT	0	Intergenic	50069..50289	50192	221	30.07	5.56E-199	50350..50667	50498	318	15.85	7.46E-57
yicO	0	Intergenic	4380906..4381144	4381052	239	30.03	2.01E-198	4380817..4381160	4380987	344	15.01	3.12E-51
CE10_4567	-102	Intergenic	4657609..4657825	4657707	217	29.87	2.19E-196	4657528..4657918	4657698	391	14.26	1.82E-46
yhfL	0	Intergenic	3944903..3945145	3945001	243	29.85	4.41E-196	3944872..3945215	3945042	344	15.59	4.10E-55
yliE	796	Intragenic	908996..909193	909096	198	29.81	1.42E-195	908922..909264	909092	343	15.10	7.78E-52
yiaY	-38	Intergenic	4222519..4222737	4222640	219	29.73	1.65E-194	4222372..4222797	4222628	426	14.97	5.94E-51
fimE	-169	Intergenic	5174629..5174833	5174736	205	29.70	3.78E-194	5174540..5174936	5174710	397	19.70	1.07E-86
sat	0	Intergenic	3517517..3517729	3517614	213	29.58	1.46E-192	3517436..3517832	3517606	397	16.11	1.07E-58
agaB	0	Intergenic	3746962..3747189	3747092	228	29.46	5.33E-191	3746910..3747252	3747086	343	12.72	2.30E-37
yajR	-72	Intergenic	429382..429611	429480	230	29.42	1.44E-190	429398..429750	429581	353	17.17	2.38E-66
hutU	-51	Intergenic	769569..769824	769727	256	29.41	1.87E-190	769528..769895	769726	368	16.58	4.87E-62
neuA	0	Intragenic	3546798..3547023	3546926	226	29.13	7.23E-187	3546711..3547074	3546941	364	16.59	4.16E-62
adiY	-41	Intergenic	4937233..4937436	4937331	204	28.95	1.46E-184	4937018..4937510	4937348	493	14.42	1.84E-47
ygcG	0	Intergenic	3256315..3256517	3256420	203	28.83	3.92E-183	3256239..3256739	3256409	501	16.32	3.71E-60
fucA	-74	Intergenic	3286052..3286271	3286149	220	28.79	1.64E-182	3286026..3286389	3286220	364	17.25	5.68E-67
CE10_3076	0	Intergenic	3138278..3138518	3138421	241	28.78	1.80E-182	3138028..3138521	3138197	494	18.51	8.55E-77
kpsM	-524	Intergenic	3550543..3550703	3550600	161	28.68	3.08E-181	3550428..3550768	3550597	341	14.89	2.02E-50
CE10_0335	-219	Intergenic	370073..370290	370171	218	28.60	3.30E-180	370014..370522	370184	509	12.13	3.69E-34
CE10_0889	0	Intergenic	941040..941265	941168	226	28.64	1.17E-180	941351..941810	941515	460	19.59	9.99E-86
fucP	133	Intragenic	3286700..3286946	3286798	247	28.41	6.66E-178	3286645..3286999	3286811	355	12.24	9.10E-35
ycdT	-65	Intergenic	1166824..1167033	1166936	210	28.40	9.38E-178	1166755..1167115	1166925	361	16.29	5.86E-60
CE10_0818	0	Intragenic	865714..865932	865835	219	28.30	1.77E-176	865675..866139	865844	465	18.92	4.21E-80
ycbQ	-5	Intergenic	1039210..1039454	1039357	245	28.17	6.27E-175	1039140..1039489	1039310	350	17.65	5.18E-70

nmpC	0	Intergenic	2501916..2502198	2502014	283	28.13	1.89E-174	2501889..2502245	2502076	357	16.88	2.96E-64
csiD	-377	Intergenic	3140357..3140564	3140454	208	28.09	6.51E-174	3140288..3140657	3140458	370	13.86	5.22E-44
CE10_3557	478	Intragenic	3627437..3627729	3627535	293	28.09	6.87E-174	3627343..3627745	3627512	403	15.56	6.65E-55
fimZ	398	Intragenic	567147..567345	567245	199	27.99	1.06E-172	567095..567597	567265	503	12.93	1.49E-38
iraP	-31	Intergenic	380127..380352	380255	226	27.98	1.63E-172	380050..380418	380220	369	16.09	1.43E-58
yfbL	0	Intergenic	2697449..2697698	2697547	250	27.94	4.75E-172	2697376..2697715	2697546	340	14.95	8.05E-51
CE10_5091	-2	Intergenic	5210721..5210948	5210852	228	27.89	1.60E-171	5210688..5211031	5210858	344	19.57	1.44E-85
leuO	-181	Intergenic	92770..92982	92887	213	27.85	5.50E-171	92719..93064	92885	346	16.43	5.60E-61
gadA	-136	Intergenic	4125363..4125562	4125465	200	27.55	1.99E-167	4125231..4125617	4125450	387	12.58	1.35E-36
ibrA	-245	Intergenic	2313456..2313668	2313582	213	27.44	4.87E-166	2313396..2313798	2313629	403	18.62	1.05E-77
gadX	-69	Intergenic	4123627..4123890	4123794	264	27.11	3.56E-162	4123422..4123929	4123760	508	15.01	3.30E-51
dusA	-308	Intergenic	4862982..4863269	4863176	288	27.02	4.14E-161	4862851..4863319	4863150	469	18.76	8.07E-79
yeiT	-45	Intergenic	2539401..2539612	2539515	212	26.90	1.15E-159	2539367..2539714	2539545	348	11.75	3.52E-32
yjeJ	0	Intergenic	4993629..4993865	4993771	237	26.86	3.01E-159	4993556..4993922	4993726	367	13.62	1.55E-42
CE10_4295	0	Intergenic	4364825..4365038	4364941	214	26.86	3.42E-159	4364740..4365136	4364908	397	16.14	6.97E-59
yebN	-218	Intergenic	2124196..2124399	2124302	204	26.81	1.17E-158	2123968..2124471	2124302	504	13.47	1.15E-41
yehD	0	Intergenic	2455198..2455489	2455392	292	26.67	5.01E-157	2455067..2455497	2455329	431	16.93	1.36E-64
CE10_0523	-40	Intergenic	575784..576005	575882	222	26.48	7.25E-155	575775..576281	575943	507	13.85	6.50E-44
tdcR	0	Intragenic	3728505..3728731	3728638	227	26.36	1.96E-153	3728426..3728879	3728593	454	15.59	4.00E-55
CE10_4234	0	Intragenic	4313033..4313233	4313131	201	26.18	2.02E-151	4312961..4313314	4313131	354	15.76	2.88E-56
ysdS	0	Intergenic	2249761..2250048	2249951	288	25.70	5.19E-146	2249711..2250083	2249914	373	16.71	5.24E-63
eivF	-67	Intergenic	3365626..3365891	3365724	266	25.52	5.28E-144	3365542..3365916	3365747	375	15.95	1.33E-57
CE10_4553	0	Intergenic	4641285..4641558	4641383	274	25.42	6.98E-143	4641242..4641633	4641404	392	16.66	1.34E-62
yiaT	0	Intergenic	4215061..4215279	4215182	219	25.34	5.40E-142	4214988..4215356	4215187	369	14.06	3.42E-45
CE10_3277	0	Intragenic	3342009..3342207	3342107	199	25.33	7.34E-142	3341909..3342253	3342079	345	16.50	1.84E-61
CE10_3982	0	Intragenic	4045106..4045312	4045203	207	25.31	1.22E-141	4045036..4045387	4045219	352	13.51	6.70E-42
matA	-95	Intergenic	280175..280390	280295	216	25.26	4.62E-141	280131..280478	280301	348	16.72	4.97E-63

CE10_4519	0	Intragenic	4605362..4605587	4605490	226	25.21	1.43E-140	4605298..4605800	4605464	503	11.92	4.75E-33
ydeK	-129	Intergenic	1743172..1743407	1743311	236	25.01	2.12E-138	1743100..1743465	1743267	366	16.05	2.67E-58
eivG	0	Intragenic	3364690..3364893	3364787	204	24.88	6.94E-137	3364593..3364953	3364758	361	12.92	1.79E-38
ybcM	0	Intragenic	571111..571326	571229	216	24.83	1.92E-136	571058..571422	571253	365	13.80	1.33E-43
CE10_0264	-129	Intergenic	289056..289330	289154	275	24.77	9.27E-136	289032..289427	289202	396	12.47	5.29E-36
ybdN	-50	Intergenic	656899..657119	656997	221	24.49	9.56E-133	656777..657283	656947	507	13.76	2.34E-43
msbB	0	Intergenic	5142221..5142418	5142322	198	24.47	1.67E-132	5142143..5142485	5142313	343	12.47	5.59E-36
yegH	-88	Intergenic	2401797..2402036	2401895	240	24.45	2.68E-132	2401624..2402086	2401919	463	14.08	2.66E-45
stpA	0	Intergenic	3150441..3150636	3150539	196	24.45	2.77E-132	3150362..3150767	3150531	406	12.87	3.51E-38
CE10_2689	-7	Intergenic	2734495..2734703	2734593	209	24.42	4.65E-132	2734379..2734787	2734618	409	14.27	1.60E-46
CE10_5099	-189	Intergenic	5220663..5220891	5220794	229	24.21	8.19E-130	5220619..5221045	5220788	427	15.24	9.50E-53
ais	0	Intergenic	2677370..2677597	2677500	228	24.21	8.62E-130	2677304..2677649	2677480	346	12.80	7.77E-38
CE10_0734	0	Intergenic	789835..790057	789932	223	23.86	3.57E-126	789781..790118	789951	338	12.32	3.55E-35
CE10_0366	0	Intragenic	403466..403659	403562	194	23.72	1.01E-124	403407..403808	403575	402	15.77	2.50E-56
rpmE2	-259	Intergenic	281827..282055	281958	229	23.64	7.77E-124	281766..282121	281935	356	13.85	6.00E-44
yjcS	0	Intergenic	4906402..4906633	4906536	232	23.51	1.54E-122	4906293..4906678	4906510	386	9.22	1.42E-20
eutS	-77	Intergenic	2884428..2884644	2884526	217	23.41	1.54E-121	2884321..2884700	2884491	380	9.29	7.52E-21
ykgI	0	Intergenic	296714..296948	296852	235	23.36	5.29E-121	296520..296971	296802	452	14.70	3.14E-49
ydeP	-21	Intergenic	1733197..1733430	1733295	234	23.31	1.76E-120	1733150..1733514	1733320	365	13.13	1.09E-39
ycbR	0	Intergenic	1039926..1040129	1040024	204	23.07	4.40E-118	1039865..1040268	1040029	404	10.34	2.23E-25
CE10_1660	0	Intergenic	1691377..1691573	1691470	197	22.86	6.44E-116	1690525..1690914	1690695	390	15.17	2.80E-52
CE10_3790	-120	Intergenic	3866170..3866358	3866272	189	22.89	2.74E-116	3866095..3866520	3866265	426	13.06	2.63E-39
ugd	-66	Intergenic	2361658..2361857	2361756	200	22.81	1.90E-115	2361443..2361917	2361748	475	12.24	1.01E-34
yeeN	-21	Intergenic	2298060..2298291	2298196	232	22.80	2.40E-115	2297991..2298362	2298196	372	14.56	2.61E-48
fimB	-128	Intergenic	5173598..5173794	5173696	197	22.72	1.51E-114	5173458..5173870	5173701	413	15.39	9.51E-54
sfmC	0	Intergenic	562157..562355	562258	199	22.65	6.27E-114	562039..562383	562208	345	10.05	4.47E-24
ttdR	344	Intragenic	3660788..3661007	3660910	220	22.60	2.18E-113	3660728..3661075	3660906	348	10.27	4.99E-25

glgS	-21	Intergenic	3647068..3647359	3647262	292	22.46	5.66E-112	3647009..3647421	3647179	413	12.54	2.37E-36
CE10_3626	0	Intragenic	3706899..3707188	3706995	290	22.45	5.89E-112	3706849..3707273	3707017	425	14.54	3.39E-48
frc	-189	Intergenic	2806933..2807132	2807029	200	22.43	1.08E-111	2806858..2807355	2807027	498	11.93	3.98E-33
yegR	0	Intergenic	2432662..2432859	2432760	198	22.30	1.90E-110	2432420..2432900	2432733	481	11.58	2.75E-31
CE10_1948	132	Intergenic	1971088..1971324	1971227	237	22.25	5.92E-110	1970982..1971354	1971152	373	16.35	2.15E-60
CE10_4706	0	Intergenic	4811342..4811545	4811448	204	22.03	6.76E-108	4811275..4811649	4811440	375	8.80	6.56E-19
ybdO	0	Intergenic	657859..658149	658053	291	22.00	1.45E-107	657677..658144	657975	468	15.67	1.13E-55
rfbC	0	Intragenic	2374416..2374657	2374560	242	21.89	1.47E-106	2374347..2374712	2374543	366	12.95	1.22E-38
focA	-136	Intergenic	994945..995227	995042	283	21.88	2.04E-106	994832..995219	995053	388	14.41	2.10E-47
CE10_0066	-319	Intergenic	78407..78640	78505	234	21.85	3.75E-106	78828..79327	78997	500	14.10	1.85E-45
yebB	0	Intergenic	2165015..2165237	2165140	223	21.75	3.18E-105	2164914..2165333	2165084	420	12.53	2.72E-36
rcsA	-174	Intergenic	2239506..2239729	2239604	224	21.68	1.76E-104	2239450..2239788	2239620	339	10.46	6.76E-26
CE10_1671	0	Intragenic	1703020..1703237	1703140	218	21.56	2.06E-103	1702934..1703307	1703104	374	11.07	8.84E-29
ypdI	0	Intergenic	2808200..2808425	2808328	226	21.50	7.97E-103	2808133..2808473	2808306	341	14.80	7.17E-50
ymgG	-64	Intergenic	1355774..1356066	1355872	293	21.20	5.18E-100	1355756..1356137	1355968	382	15.15	3.68E-52
yobF	-398	Intergenic	2126920..2127135	2127038	216	21.14	1.83E-99	2126725..2127180	2127011	456	12.23	1.04E-34
CE10_4717	-163	Intragenic	4821040..4821329	4821234	290	21.02	2.00E-98	4820987..4821333	4821157	347	10.77	2.37E-27
nanC	0	Intergenic	5172449..5172718	5172547	270	21.01	2.52E-98	5172384..5172835	5172554	452	14.70	3.17E-49
hlyE	0	Intergenic	1363183..1363379	1363279	197	20.96	7.77E-98	1363069..1363423	1363254	355	13.55	3.71E-42
yfdF	0	Intragenic	2776998..2777270	2777173	273	20.95	8.77E-98	2776795..2777230	2776961	436	14.96	7.15E-51
yahL	0	Intergenic	326795..327027	326893	233	20.81	1.72E-96	326738..327153	326908	416	13.59	2.19E-42
CE10_1661	-8	Intergenic	1692752..1692955	1692849	204	20.58	2.21E-94	1692610..1692983	1692814	374	13.32	8.73E-41
nanR	-71	Intergenic	3825000..3825209	3825116	210	20.45	3.06E-93	3824959..3825338	3825169	380	12.17	2.19E-34
yjdA	-14	Intergenic	4926152..4926382	4926250	231	20.44	4.06E-93	4926072..4926458	4926239	387	12.87	3.31E-38
yiiE	-72	Intergenic	4643647..4643845	4643748	199	20.43	4.16E-93	4643595..4643982	4643765	388	12.91	2.08E-38
dpiB	-28	Intergenic	670822..671031	670934	210	20.33	3.28E-92	670615..671120	670952	506	15.25	8.14E-53
CE10_0275	0	Intragenic	303267..303554	303457	288	20.26	1.47E-91	303067..303573	303404	507	13.71	4.24E-43

feoA	-134	Intergenic	3980944..3981174	3981042	231	20.19	5.68E-91	3980903..3981321	3981073	419	9.01	1.07E-19
ybbW	0	Intergenic	540830..541052	540928	223	20.13	1.87E-90	540772..541143	540942	372	10.72	4.05E-27
CE10_0560	185	Intergenic	604888..605102	605005	215	20.06	8.20E-90	604795..605170	604965	376	13.01	5.60E-39
CE10_0290	0	Intergenic	320948..321212	321041	265	19.64	3.26E-86	320887..321231	321063	345	12.97	9.13E-39
CE10_0724	-72	Intergenic	777616..777815	777712	200	19.65	2.72E-86	777554..777900	777723	347	14.94	8.78E-51
ompN	-2	Intergenic	1605511..1605798	1605702	288	19.58	1.11E-85	1605472..1605820	1605642	349	13.45	1.44E-41
CE10_4709	0	Intergenic	4813131..4813407	4813229	277	19.27	4.69E-83	4813065..4813417	4813250	353	9.35	4.26E-21
CE10_3786	35	Intragenic	3862473..3862763	3862666	291	19.14	5.35E-82	3862492..3862833	3862662	342	10.72	3.94E-27
CE10_3301	53	Intragenic	3367459..3367708	3367557	250	18.98	1.32E-80	3366310..3366720	3366556	411	13.31	1.06E-40
yiaO	0	Intragenic	4204947..4205166	4205070	220	18.61	1.22E-77	4204828..4205239	4205070	412	10.19	1.07E-24
ydjO	0	Intergenic	2033450..2033735	2033638	286	18.14	7.78E-74	2033079..2033399	2033253	321	15.98	8.73E-58
yhbX	345	Intragenic	3778658..3778875	3778756	218	18.14	8.12E-74	3778577..3778957	3778747	381	10.94	3.79E-28
tsx2	0	Intergenic	4700453..4700681	4700551	229	17.90	5.95E-72	4700384..4700722	4700553	339	10.47	5.83E-26
yfgF	-101	Intragenic	2937553..2937750	2937653	198	17.88	7.91E-72	2937413..2937810	2937641	398	11.57	2.80E-31
araE	-58	Intergenic	3334534..3334729	3334632	196	17.83	2.18E-71	3334435..3334801	3334632	367	9.48	1.26E-21
agaC	0	Intragenic	3747549..3747788	3747647	240	17.68	3.24E-70	3747454..3747902	3747735	449	6.71	9.92E-12
cyoA	-269	Intergenic	436794..436990	436892	197	17.66	4.01E-70	436528..437034	436865	507	10.36	1.87E-25
pagP	0	Intergenic	675237..675495	675335	259	17.67	3.40E-70	675121..675484	675316	364	14.64	7.51E-49
ybeF	-190	Intergenic	680395..680594	680499	200	17.57	2.07E-69	679992..680353	680162	362	12.22	1.15E-34
yjgN	0	Intragenic	5110033..5110322	5110225	290	17.41	3.40E-68	5109929..5110378	5110099	450	12.60	1.01E-36
yahA	-152	Intergenic	310056..310291	310195	236	17.01	3.32E-65	309708..310090	309878	383	11.29	6.96E-30
ydjE	769	Intragenic	2071605..2071861	2071764	257	16.85	5.06E-64	2071601..2071970	2071771	370	10.62	1.14E-26
CE10_0258	0	Intergenic	283608..283893	283706	286	16.89	2.65E-64	283551..283948	283779	398	13.64	1.13E-42
yceJ	0	Intragenic	1190137..1190368	1190234	232	16.73	3.88E-63	1190093..1190540	1190263	448	9.63	3.09E-22
yciD	-271	Intergenic	1500835..1501071	1500974	237	16.59	3.97E-62	1500310..1500676	1500509	367	9.52	8.60E-22
mocA	0	Intragenic	3383931..3384148	3384051	218	16.51	1.57E-61	3383875..3384289	3384044	415	5.89	1.92E-09
CE10_5101	0	Intragenic	5223835..5224127	5223933	293	16.20	2.44E-59	5223824..5224191	5223994	368	7.57	1.94E-14

sfmH	159	Intragenic	565835..566060	565930	226	15.67	1.15E-55	565700..566062	565869	363	8.46	1.34E-17
gadB	-153	Intergenic	1718885..1719158	1719062	274	15.48	2.52E-54	1718763..1719137	1718933	375	11.14	3.85E-29
yffB	0	Intergenic	2899548..2899793	2899696	246	15.40	7.69E-54	2899433..2899823	2899602	391	11.45	1.12E-30
neuO	269	Intragenic	839812..840043	839946	232	14.82	5.43E-50	839606..839902	839776	297	8.94	1.89E-19
CE10_4222	0	Intergenic	4299049..4299300	4299203	252	14.48	8.13E-48	4298934..4299442	4299104	509	8.65	2.55E-18

Table S3. Binding sites identified by ChIP-seq for NMEC DsdC2^{FLAG} in M9 minimal media

			Replicate 1					Replicate 2				
**Gene**	**Dist. Gene**	**Context**	**Co-ordinates**	**Centre**	**Length**	**Peak score**	**P-value**	**Co-ordinates**	**Centre**	**Length**	**Peak score**	**P-value**
CE10_0023	-534	Intragenic	27362..27811	27681	450	39.18	0.00E+00	27442..27871	27704	430	31.11	9.35E-213
ycgX	-686	Intergenic	1342133..1342499	1342311	367	40.61	0.00E+00	1342023..1342527	1342193	505	31.25	1.23E-214
wbbC	0	Intragenic	2367810..2368203	2367985	394	47.54	0.00E+00	2367814..2368253	2367984	440	40.19	0.00E+00
dsdC1	0	Intergenic	2791240..2791618	2791420	379	71.00	0.00E+00	2791226..2791600	2791395	375	66.07	0.00E+00
waaT	0	Intragenic	4262774..4263197	4263021	424	40.68	0.00E+00	4262768..4263185	4263017	418	27.42	8.06E-166
dsdC2	0	Intergenic	4332279..4332649	4332458	371	71.53	0.00E+00	4332276..4332631	4332446	356	64.40	0.00E+00
CE10_4270	0	Intragenic	4347265..4347784	4347430	520	42.11	0.00E+00	4347264..4347758	4347434	495	32.98	7.32E-239
CE10_4363	1061	Intragenic	4444704..4445178	4445027	475	39.22	0.00E+00	4445502..4445873	4445654	372	32.90	1.25E-237
lpfA	0	Intergenic	4456422..4456804	4456599	383	39.00	0.00E+00	4456392..4456771	4456628	380	22.12	1.11E-108
vioA	0	Intragenic	2372516..2372867	2372689	352	38.39	8.90E-323	2372423..2372664	2372593	242	26.10	1.76E-150
wzy	0	Intergenic	2368896..2369216	2369049	321	37.95	1.82E-315	2368871..2369265	2369173	395	29.62	4.50E-193
neuB	-421	Intergenic	3548330..3548635	3548441	306	37.16	1.37E-302	3548251..3548595	3548412	345	29.67	9.22E-194
CE10_2740	-18	Intergenic	2788678..2789063	2788885	386	37.13	4.43E-302	2788665..2789166	2789003	502	27.92	7.81E-172
waaV	0	Intergenic	4259991..4260437	4260258	447	36.74	7.38E-296	4260082..4260441	4260280	360	29.24	2.76E-188
rfaL	499	Intragenic	4258236..4258626	4258447	391	36.71	2.78E-295	4258276..4258652	4258442	377	26.45	1.61E-154
epaS	188	Intragenic	3354065..3354430	3354251	366	36.18	5.54E-287	3354022..3354464	3354304	443	30.59	8.69E-206
ubiC	706	Intragenic	4853339..4853690	4853591	352	35.84	1.30E-281	4853266..4853700	4853584	435	30.97	7.05E-211
yfcV	-55	Intergenic	2769752..2770201	2769931	450	35.65	1.33E-278	2769693..2770190	2770021	498	22.91	1.69E-116
yjbM	0	Intragenic	4862245..4862634	4862363	390	35.34	7.55E-274	4862245..4862639	4862414	395	24.38	1.45E-131
yjiC	0	Intergenic	5189271..5189805	5189628	535	34.73	1.45E-264	5189302..5189756	5189464	455	27.15	1.38E-162
espX1	0	Intergenic	28370..28773	28600	404	34.60	1.10E-262	28209..28538	28287	330	24.60	6.41E-134
ygiL	0	Intergenic	3641569..3642041	3641733	473	34.37	3.65E-259	3641572..3642003	3641740	432	22.42	1.39E-111
yhaC	667	Intragenic	3730287..3730712	3730466	426	33.89	4.99E-252	3730447..3730759	3730597	313	27.38	2.64E-165

CE10_4160	0	Intragenic	4233464..4233945	4233766	482	33.78	2.26E-250	4233513..4233854	4233685	342	28.55	1.41E-179
ybjQ	-414	Intragenic	942239..942775	942418	537	33.29	2.53E-243	942545..942818	942645	274	22.96	5.34E-117
CE10_0067	0	Intergenic	79472..79809	79662	338	33.28	4.14E-243	79624..80018	79862	395	27.50	7.90E-167
tauA	-100	Intergenic	365174..365547	365353	374	33.17	1.34E-241	365200..365553	365384	354	24.00	1.45E-127
espX4	0	Intragenic	4852588..4853001	4852768	414	33.00	4.71E-239	4852613..4852982	4852813	370	26.11	1.46E-150
yjbE	0	Intergenic	4839307..4839675	4839496	369	32.84	6.74E-237	4839305..4839662	4839494	358	22.32	1.31E-110
hutU	-14	Intergenic	769437..769861	769682	425	32.83	1.17E-236	769481..769849	769651	369	21.76	2.76E-105
CE10_4870	0	Intergenic	4974849..4975240	4975061	392	32.64	5.12E-234	4974865..4975238	4975086	374	21.66	2.38E-104
yhhZ	0	Intergenic	4028972..4029264	4029151	293	32.44	3.40E-231	4029028..4029274	4029198	247	21.62	5.48E-104
CE10_4297	0	Intergenic	4367341..4367731	4367502	391	32.24	2.40E-228	4367294..4367769	4367619	476	22.03	7.47E-108
kpsF	-102	Intergenic	3533379..3533911	3533557	533	32.16	3.43E-227	3533361..3533868	3533531	508	26.57	6.94E-156
ibrA	-228	Intergenic	2313439..2313870	2313580	432	32.10	2.33E-226	2313352..2313794	2313522	443	22.70	2.46E-114
dusA	-227	Intragenic	4862943..4863300	4863027	358	32.06	6.78E-226	4862921..4863413	4863244	493	25.42	8.59E-143
CE10_4943	0	Intergenic	5047548..5047940	5047770	393	31.99	6.65E-225	5047602..5047974	5047772	373	25.44	4.37E-143
CE10_0818	0	Intragenic	865658..866109	865838	452	30.36	9.36E-203	865680..866090	865850	411	21.94	5.01E-107
yqeI	0	Intergenic	3340261..3340795	3340619	535	30.22	5.84E-201	3340376..3340781	3340630	406	21.86	2.98E-106
sipB	0	Intergenic	4362531..4363052	4362886	522	30.20	1.32E-200	4362545..4362997	4362828	453	22.61	1.78E-113
yfaL	0	Intergenic	2651212..2651655	2651388	444	30.02	3.05E-198	2650598..2651022	2650754	425	16.62	2.54E-62
cmtB	0	Intragenic	3449979..3450401	3450155	423	29.63	3.05E-193	3449934..3450266	3450154	333	18.28	6.45E-75
leuO	-103	Intergenic	92697..93060	92877	364	29.40	2.90E-190	92685..93057	92888	373	22.08	2.35E-108
sfmA	0	Intergenic	561144..561543	561365	400	29.20	9.68E-188	561186..561544	561375	359	21.73	5.48E-105
nmpC	0	Intergenic	2501882..2502248	2502062	367	28.97	9.00E-185	2501915..2502252	2502082	338	19.39	4.42E-84
envR	0	Intergenic	3874432..3874842	3874602	411	28.92	3.50E-184	3874441..3874892	3874608	452	22.00	1.39E-107
CE10_1948	0	Intergenic	1970999..1971344	1971161	346	28.50	6.71E-179	1970975..1971377	1971145	403	19.82	9.16E-88
ykgI	0	Intragenic	296587..297000	296821	414	28.40	1.03E-177	296534..296957	296802	424	22.66	5.01E-114
CE10_4228	-48	Intergenic	4305007..4305416	4305187	410	28.36	2.78E-177	4305007..4305370	4305176	364	22.98	3.90E-117
yadN	-20	Intergenic	166962..167347	167171	386	28.21	2.17E-175	166987..167325	167156	339	26.00	2.42E-149

yhaI	0	Intergenic	3713935..3714331	3714114	397	28.10	5.08E-174	3713932..3714346	3714096	415	20.29	7.53E-92
CE10_2939	0	Intergenic	2996428..2996801	2996622	374	27.97	1.64E-172	2996395..2996787	2996556	393	18.00	1.02E-72
ecpD	0	Intergenic	166089..166455	166319	367	27.85	5.10E-171	166137..166580	166307	444	17.59	1.51E-69
ysdS	-2	Intergenic	2249696..2249982	2249774	287	27.75	9.91E-170	2249697..2250095	2249928	399	21.50	8.53E-103
fimE	0	Intergenic	5174540..5175005	5174719	466	27.54	2.90E-167	5174553..5174991	5174706	439	22.83	1.24E-115
eivF	0	Intergenic	3365537..3365942	3365766	406	27.53	3.43E-167	3365476..3365951	3365641	476	19.67	2.06E-86
CE10_5099	-53	Intergenic	5220615..5221027	5220787	413	27.52	4.82E-167	5220846..5221065	5220944	220	16.31	3.91E-60
CE10_2689	0	Intergenic	2734378..2734801	2734629	424	27.42	8.09E-166	2734336..2734811	2734648	476	19.92	1.47E-88
CE10_4295	0	Intragenic	4364621..4365097	4364979	477	27.42	8.67E-166	4364636..4365088	4364974	453	19.6	7.28E-86
yfdF	0	Intergenic	2776752..2777229	2776930	478	27.29	2.97E-164	2776797..2777268	2776946	472	19.20	2.01E-82
ycbQ	0	Intergenic	1039076..1039514	1039335	439	27.10	5.13E-162	1039143..1039484	1039313	342	21.61	7.36E-104
yiiG	0	Intergenic	4651354..4651747	4651569	394	27.07	9.84E-162	4651373..4651738	4651542	366	16.28	6.90E-60
CE10_3508	0	Intergenic	3578622..3579000	3578826	379	26.40	6.22E-154	3578596..3578958	3578866	363	18.54	4.72E-77
CE10_3627	0	Intergenic	3707535..3707897	3707711	363	26.29	1.21E-152	3707473..3707903	3707634	431	21.28	8.84E-101
ymgG	0	Intergenic	1355705..1356177	1355885	473	26.25	3.86E-152	1355703..1356174	1355872	472	19.32	1.75E-83
fucA	-48	Intergenic	3286026..3286411	3286206	386	25.95	8.73E-149	3285981..3286374	3286151	394	22.70	2.09E-114
torY	0	Intergenic	2176727..2177084	2176887	358	25.91	2.30E-148	2176744..2177192	2176914	449	18.89	6.53E-80
kpsM	-372	Intergenic	3550391..3550760	3550608	370	25.91	2.32E-148	3550432..3550730	3550509	299	22.53	1.12E-112
matA	-57	Intergenic	280137..280561	280314	425	25.78	7.16E-147	280121..280598	280262	478	19.67	2.11E-86
CE10_4598	0	Intragenic	4685849..4686220	4686045	372	25.76	1.38E-146	4685779..4686266	4686099	488	16.34	2.64E-60
ydjO	225	Intragenic	2033083..2033319	2033244	237	25.71	4.06E-146	2033342..2033737	2033576	396	20.10	3.47E-90
yfbL	0	Intergenic	2697381..2697743	2697560	363	25.64	2.99E-145	2697379..2697799	2697547	421	18.24	1.31E-74
ygcG	0	Intergenic	3256240..3256635	3256419	396	25.60	6.84E-145	3256265..3256590	3256454	326	20.01	2.13E-89
yhfL	0	Intergenic	3944842..3945211	3945021	370	25.55	2.46E-144	3944850..3945263	3945015	414	21.97	2.95E-107
ycdT	0	Intergenic	1166750..1167135	1166927	386	25.37	2.46E-142	1166695..1167145	1166976	451	20.23	2.84E-91
yliE	650	Intragenic	908860..909306	909139	447	25.12	1.70E-139	908896..909299	909149	404	17.68	2.95E-70
CE10_4613	0	Intergenic	4703706..4704134	4703859	429	24.94	1.40E-137	4703716..4704119	4703886	404	19.80	1.55E-87

ydeK	-14	Intergenic	1743057..1743518	1743342	462	24.82	2.95E-136	1743081..1743453	1743251	373	16.77	1.87E-63
yiaY	0	Intergenic	4222409..4222788	4222589	380	24.81	3.93E-136	4222384..4222793	4222553	410	17.88	8.17E-72
caiT	0	Intergenic	50024..50329	50201	306	24.71	4.09E-135	50023..50271	50191	249	22.75	7.11E-115
iraP	0	Intergenic	380060..380434	380239	375	24.70	5.10E-135	379991..380420	380286	430	18.83	2.05E-79
yajR	-8	Intergenic	429318..429824	429492	507	24.68	9.68E-135	429366..429789	429533	424	22.36	4.88E-111
yghJ	0	Intragenic	3567434..3567898	3567611	465	24.63	2.95E-134	3567470..3567875	3567638	406	21.57	1.60E-103
pagP	-249	Intergenic	674770..675139	674944	370	24.43	3.87E-132	675168..675524	675334	357	17.03	2.58E-65
CE10_4234	0	Intragenic	4312928..4313380	4313105	453	24.36	2.38E-131	4312961..4313301	4313131	341	18.80	3.97E-79
pitB	0	Intergenic	3591899..3592395	3592078	497	24.19	1.32E-129	3591909..3592352	3592183	444	17.13	4.22E-66
ybdO	0	Intergenic	657734..658132	657967	399	24.17	2.06E-129	657795..658194	658025	400	16.88	2.99E-64
rfbC	0	Intragenic	2374333..2374738	2374559	406	24.07	2.65E-128	2374329..2374695	2374569	367	16.23	1.62E-59
setC	0	Intergenic	4358051..4358447	4358274	397	24.00	1.32E-127	4358061..4358437	4358231	377	18.77	7.13E-79
ais	0	Intergenic	2677273..2677731	2677556	459	23.89	1.78E-126	2677300..2677681	2677521	382	12.92	1.81E-38
CE10_3398	0	Intergenic	3470812..3471198	3471026	387	23.74	6.90E-125	3470820..3471244	3470982	425	19.09	1.41E-81
hipB	-500	Intragenic	1739028..1739535	1739358	508	23.73	8.72E-125	1739163..1739521	1739352	359	15.77	2.40E-56
CE10_3626	0	Intergenic	3706823..3707193	3707003	371	23.68	2.74E-124	3706855..3707229	3707025	375	20.42	5.77E-93
fimB	0	Intergenic	5173476..5174004	5173646	529	23.23	1.21E-119	5173463..5173786	5173685	324	14.36	4.73E-47
fdrA	0	Intergenic	549610..549982	549790	373	23.07	4.95E-118	549513..549940	549801	428	18.20	2.69E-74
sat	0	Intergenic	3517461..3517916	3517634	456	23.14	1.00E-118	3517411..3517844	3517677	434	18.28	5.79E-75
tnaA	0	Intergenic	4430054..4430587	4430234	534	23.01	1.80E-117	4430209..4430597	4430434	389	16.24	1.23E-59
yqiG	0	Intragenic	3642402..3642914	3642582	513	22.96	6.12E-117	3642397..3642702	3642630	306	18.90	5.57E-80
garP	0	Intragenic	3735483..3735991	3735662	509	22.84	9.76E-116	3735486..3735929	3735640	444	20.78	3.60E-96
yghT	0	Intergenic	3589485..3589864	3589685	380	22.73	1.17E-114	3589507..3589892	3589723	386	12.92	1.65E-38
CE10_4567	0	Intergenic	4657542..4657936	4657770	395	22.67	4.47E-114	4657524..4657832	4657743	309	17.61	9.52E-70
CE10_2267	-177	Intergenic	2290168..2290519	2290432	352	22.60	2.20E-113	2290164..2290552	2290331	389	20.56	2.86E-94
CE10_3557	500	Intragenic	3627487..3627733	3627536	247	22.54	9.24E-113	3627485..3627894	3627655	410	18.17	4.27E-74
yiaT	0	Intergenic	4214955..4215397	4215127	443	22.38	3.12E-111	4214986..4215330	4215154	345	19.27	4.85E-83

yicO	0	Intergenic	4380807..4381176	4380977	370	22.28	2.84E-110	4380736..4381220	4381051	485	17.01	3.75E-65
yegH	-48	Intergenic	2401698..2402076	2401876	379	22.25	5.27E-110	2401645..2402032	2401772	388	20.18	7.60E-91
yeeN	0	Intergenic	2298026..2298400	2298206	375	22.24	7.14E-110	2297949..2298402	2298256	454	18.24	1.18E-74
CE10_0275	0	Intragenic	303207..303549	303328	343	22.01	1.07E-107	303163..303556	303388	394	15.39	9.07E-54
speF	-275	Intergenic	730641..731053	730874	413	21.99	1.91E-107	730585..731020	730853	436	16.54	9.40E-62
fimA	-167	Intergenic	5175542..5175912	5175798	371	21.98	2.15E-107	5175600..5175850	5175782	251	18.40	6.78E-76
CE10_3982	0	Intragenic	4045022..4045392	4045213	371	21.94	5.01E-107	4044997..4045435	4045146	439	16.90	2.07E-64
yfgH	0	Intergenic	2938010..2938381	2938208	372	21.68	1.47E-104	2938055..2938475	2938221	421	16.89	2.75E-64
nanC	0	Intergenic	5172434..5172888	5172605	455	21.49	1.06E-102	5172427..5172782	5172614	356	14.91	1.34E-50
CE10_1661	0	Intergenic	1692553..1693004	1692835	452	21.36	1.53E-101	1692581..1693019	1692850	439	15.50	1.74E-54
yiaW	0	Intergenic	4217591..4217912	4217775	322	21.27	9.98E-101	4217635..4218015	4217800	381	16.50	1.77E-61
dpiB	0	Intergenic	670720..671107	670863	388	21.14	1.83E-99	670710..671140	670973	431	15.17	2.79E-52
gadX	0	Intergenic	4123396..4123905	4123732	510	21.13	1.90E-99	4123414..4123921	4123752	508	23.02	1.63E-117
adiY	0	Intergenic	4937134..4937553	4937314	420	21.07	7.88E-99	4937053..4937508	4937362	456	18.07	2.93E-73
gadA	0	Intergenic	4125210..4125622	4125383	413	21.06	8.81E-99	4125220..4125686	4125373	467	16.49	2.15E-61
stpA	0	Intergenic	3150326..3150740	3150505	415	21.04	1.51E-98	3150388..3150759	3150596	372	17.29	2.76E-67
yebB	0	Intergenic	2164887..2165369	2165056	483	21.02	2.07E-98	2164900..2165404	2165070	505	16.53	1.06E-61
fucP	75	Intragenic	3286606..3287099	3286748	494	20.94	1.15E-97	3286596..3287010	3286764	415	15.57	5.54E-55
ykgE	-86	Intergenic	299401..299767	299579	367	20.89	3.46E-97	299337..299757	299588	421	12.37	1.86E-35
CE10_0735	-119	Intergenic	790984..791457	791318	474	20.58	2.25E-94	791118..791462	791299	345	15	3.46E-51
rpmE2	-218	Intergenic	281786..282149	281959	364	20.38	1.37E-92	281769..282123	281954	355	14.38	3.63E-47
CE10_0366	0	Intragenic	403367..403755	403545	389	20.31	4.75E-92	403415..403787	403585	373	16.36	1.88E-60
fimZ	285	Intragenic	567051..567472	567230	422	20.26	1.56E-91	567094..567464	567264	371	18.42	4.27E-76
csiD	-967	Intragenic	3139530..3139974	3139705	445	19.98	4.09E-89	3139562..3139981	3139812	420	15.30	4.07E-53
ypdI	0	Intergenic	2808118..2808499	2808324	382	19.87	3.40E-88	2808126..2808526	2808357	401	14.92	1.18E-50
yobF	-268	Intergenic	2126790..2127203	2127024	414	19.84	7.30E-88	2126793..2127148	2126962	356	16.05	2.65E-58
CE10_0724	0	Intergenic	777533..777898	777683	366	19.29	3.45E-83	777556..777950	777645	395	14.10	1.95E-45

glgS	0	Intergenic	3647029..3647437	3647258	409	19.26	6.00E-83	3647008..3647465	3647178	458	14.14	1.05E-45
yjdA	0	Intergenic	4926058..4926477	4926298	420	19.18	2.80E-82	4926030..4926402	4926289	373	14.39	3.04E-47
frc	0	Intergenic	2806899..2807342	2807163	444	19.09	1.45E-81	2806880..2807282	2807017	403	14.00	7.72E-45
yebN	-138	Intergenic	2124095..2124479	2124275	385	19.09	1.53E-81	2123997..2124498	2124330	502	15.84	7.66E-57
ytcA	0	Intergenic	4904194..4904592	4904372	399	19.01	7.52E-81	4904214..4904550	4904382	337	16.48	2.48E-61
lfpB	0	Intragenic	4455596..4455976	4455809	381	18.99	1.06E-80	4455579..4456010	4455842	432	16.73	4.25E-63
yihN	0	Intergenic	4624721..4625143	4624988	423	18.68	3.42E-78	4624732..4625142	4624976	411	14.17	7.21E-46
neuO	180	Intragenic	839731..840037	839842	307	18.2	2.50E-74	839122..839433	839361	312	9.66	2.24E-22
CE10_4222	0	Intergenic	4299056..4299433	4299236	378	14.44	1.43E-47	4299050..4299330	4299238	281	11.78	2.40E-32

Table S4. Binding sites identified by ChIP-seq for NMEC $DsdC2^{FLAG}$ in M9 minimal media plus 1 mM D-ser

			**Replicate 1**					**Replicate 2**				
**Gene**	**Dist. Gene**	**Context**	**Co-ordinates**	**Centre**	**Length**	**Peak score**	**P-value**	**Co-ordinates**	**Centre**	**Length**	**Peak score**	**P-value**
dsdC1	0	Intergenic	2791201..2791623	2791424	423	59.95	0.00E+00	2791239..2791631	2791405	393	60.87	0.00E+00
dsdC2	0	Intergenic	4332245..4332661	4332462	417	58.92	0.00E+00	4332276..4332653	4332446	378	61.24	0.00E+00
wbbC	0	Intragenic	2367801..2368218	2368019	418	33.47	7.61E-246	2367849..2368187	2368018	339	48.73	0.00E+00
CE10_4270	0	Intragenic	4347302..4347887	4347499	586	29.89	1.37E-196	4347239..4347682	4347409	444	48.52	0.00E+00
CE10_4160	0	Intragenic	4233415..4233971	4233772	557	27.43	6.72E-166	4233478..4233946	4233779	469	48.93	0.00E+00
wzy	-61	Intergenic	2368941..2369336	2369143	396	27.16	9.88E-163	2368949..2369329	2369075	381	45.96	0.00E+00
CE10_0023	-567	Intragenic	27395..27867	27682	473	27.05	1.75E-161	27398..27802	27567	405	51.19	0.00E+00
neuB	-351	Intergenic	3548260..3548659	3548505	400	26.93	5.44E-160	3548210..3548563	3548377	354	44.34	0.00E+00
CE10_2740	-31	Intergenic	2788691..2789078	2788926	388	26.78	2.70E-158	2789250..2789602	2789408	353	42.91	0.00E+00
waaV	0	Intergenic	4259986..4260392	4260149	407	26.73	9.89E-158	4260036..4260393	4260224	358	46.44	0.00E+00
ygiL	0	Intergenic	3641588..3642068	3641783	481	26.42	3.73E-154	3641569..3642072	3641737	504	47.10	0.00E+00
espX4	0	Intragenic	4852560..4852976	4852760	417	26.11	1.24E-150	4852574..4852929	4852769	356	44.53	0.00E+00
rfaL	419	Intragenic	4258187..4258738	4258380	552	25.71	4.45E-146	4258266..4258601	4258436	336	54.31	0.00E+00
epaS	154	Intragenic	3353928..3354450	3354126	523	25.57	1.66E-144	3354034..3354408	3354242	375	48.11	0.00E+00
yjiC	0	Intergenic	5189241..5189815	5189623	575	25.55	2.49E-144	5189297..5189760	5189591	464	41.38	0.00E+00
ycgX	600	Intergenic	1342145..1342585	1342410	441	25.53	5.17E-144	1342107..1342478	1342309	372	45.12	0.00E+00
waaT	0	Intragenic	4262782..4263209	4263010	428	25.34	5.22E-142	4262767..4263185	4263019	419	47.57	0.00E+00
espY4	349	Intragenic	4441008..4441461	4441273	454	25.17	4.53E-140	4440975..4441325	4441190	351	37.02	2.83E-300
CE10_4870	0	Intergenic	4974839..4975298	4975038	460	24.86	9.58E-137	4974862..4975255	4975032	394	52.93	0.00E+00
CE10_0067	0	Intragenic	79527..80036	79718	510	24.71	4.12E-135	79505..79780	79674	276	38.64	0.00E+00
prgI	-15	Intragenic	3350439..3350870	3350639	432	24.22	6.54E-130	3350441..3350883	3350611	443	46.29	0.00E+00
yhhZ	0	Intergenic	4028998..4029273	4029197	276	23.69	2.42E-124	4028968..4029295	4029138	328	47.58	0.00E+00
yfaL	0	Intergenic	2651189..2651693	2651494	505	23.56	5.57E-123	2651235..2651598	2651405	364	34.92	1.92E-267

yjbM	70	Intragenic	4862267..4862581	4862385	315	22.48	3.24E-112	4862932..4863330	4863089	399	35.44	2.32E-275
kpsF	-135	Intergenic	3533449..3533878	3533679	430	22.44	8.64E-112	3533422..3533766	3533614	345	36.92	1.14E-298
ubiC	-640	Intragenic	4853358..4853756	4853561	399	22.44	8.80E-112	4853286..4853763	4853597	478	48.02	0.00E+00
CE10_4297	0	Intergenic	4367262..4367846	4367652	585	22.07	2.91E-108	4367300..4367779	4367611	480	44.46	0.00E+00
tauA	-124	Intergenic	365045..365523	365326	479	21.95	4.47E-107	365186..365528	365359	343	46.10	0.00E+00
ibrA	-150	Intergenic	2313361..2313946	2313549	586	21.42	4.04E-102	2313418..2313818	2313583	401	36.60	1.44E-293
yhaC	601	Intragenic	3730248..3730669	3730558	422	21.37	1.23E-101	3730146..3730645	3730316	500	40.36	0.00E+00
eivC	-386	Intragenic	3360288..3360700	3360502	413	21.07	7.30E-99	3360273..3360679	3360443	407	36.54	1.52E-292
yghJ	0	Intragenic	3567495..3567903	3567694	409	20.82	1.32E-96	3567412..3567851	3567579	440	35.35	5.16E-274
eprH	0	Intergenic	3351518..3352032	3351839	515	20.81	1.81E-96	3351593..3351969	3351800	377	40.77	0.00E+00
envR	0	Intergenic	3874394..3874832	3874633	439	20.60	1.33E-94	3874434..3874804	3874604	371	39.53	0.00E+00
dusA	-251	Intragenic	4862922..4863326	4863120	405	20.14	1.79E-90	4862932..4863330	4863089	399	35.44	2.32E-275
ybcK	0	Intragenic	569700..570111	569916	412	20.05	9.24E-90	569742..570133	569908	392	36.60	1.21E-293
CE10_3508	0	Intergenic	3578606..3579015	3578816	410	19.78	2.43E-87	3578606..3579032	3578863	427	34.65	2.23E-263
setC	0	Intergenic	4357955..4358444	4358246	490	19.71	8.58E-87	4358028..4358401	4358232	374	31.60	2.00E-219
espX1	0	Intergenic	28309..28800	28603	492	19.79	1.97E-87	28392..28756	28559	365	39.58	0.00E+00
sfmA	0	Intergenic	561132..561550	561351	419	19.56	1.71E-85	561191..561560	561361	370	32.63	7.04E-234
CE10_0818	-110	Intragenic	865607..866128	865806	522	19.52	3.95E-85	865643..866088	865813	446	40.74	0.00E+00
CE10_3398	-4	Intergenic	3470772..3471177	3470964	406	19.37	6.67E-84	3470777..3471175	3470947	399	31.99	7.28E-225
yjbE	0	Intergenic	4839264..4839724	4839462	461	19.23	9.84E-83	4839262..4839689	4839521	428	39.43	0.00E+00
CE10_4943	0	Intergenic	5047521..5048012	5047717	492	19.25	7.13E-83	5047518..5047942	5047773	425	42.86	0.00E+00
sipB	0	Intergenic	4362518..4363039	4362894	522	19.16	3.76E-82	4362542..4363020	4362854	479	42.52	0.00E+00
gadX	0	Intergenic	4123530..4123934	4123735	405	19.09	1.66E-81	4123408..4123914	4123745	507	30.27	1.24E-201
eivF	0	Intergenic	3365516..3365886	3365686	371	18.92	3.69E-80	3365504..3365952	3365783	449	27.09	5.75E-162
ysdS	0	Intergenic	2249552..2250108	2249911	557	18.91	4.69E-80	2249708..2250086	2249917	379	29.92	4.66E-197
CE10_4228	0	Intergenic	4304996..4305491	4305195	496	18.75	8.81E-79	4305009..4305514	4305179	506	36.15	1.88E-286
yehD	0	Intergenic	2455112..2455543	2455344	432	18.64	6.98E-78	2455141..2455485	2455307	345	24.64	2.52E-134

CE10_3557	0	Intergenic	3628241..3628807	3628437	567	18.71	2.16E-78	3628255..3628751	3628424	497	33.52	1.25E-246
rfaY	0	Intragenic	4261738..4262158	4261857	421	18.31	3.44E-75	4261801..4262123	4261980	323	35.64	1.47E-278
lpfA	0	Intergenic	4456369..4456823	4456624	455	18.28	5.57E-75	4456471..4456810	4456642	340	47.19	0.00E+00
fimZ	271	Intragenic	567067..567513	567317	447	18.20	2.79E-74	567021..567429	567266	409	26.02	1.58E-149
ycbQ	0	Intergenic	1039119..1039559	1039363	441	18.13	8.68E-74	1039098..1039524	1039356	427	37.70	2.36E-311
yiaY	0	Intergenic	4222437..4222812	4222611	376	18.06	3.07E-73	4222380..4222772	4222603	393	31.66	3.22E-220
neuA	0	Intragenic	3546713..3547161	3546913	449	18.05	4.29E-73	3546735..3547143	3546897	409	32.16	3.46E-227
cmtB	0	Intragenic	3449964..3450395	3450164	432	18.02	6.85E-73	3449903..3450404	3450235	502	31.50	4.11E-218
tnaA	0	Intergenic	4430163..4430581	4430363	419	18.00	9.87E-73	4430328..4430607	4430441	280	28.93	2.34E-184
yliE	583	Intragenic	908813..909295	909096	483	17.82	2.28E-71	908913..909264	909096	352	26.68	4.15E-157
hutU	-18	Intergenic	769262..769857	769658	596	17.84	1.78E-71	769533..769887	769718	355	41.38	0.00E+00
nmpC	0	Intergenic	2501805..2502289	2502090	485	17.70	2.12E-70	2501843..2502269	2502013	427	36.42	9.50E-291
yajR	0	Intergenic	429282..429793	429477	512	17.61	9.81E-70	429395..429749	429580	355	34.22	5.17E-257
CE10_4598	0	Intragenic	4685781..4686273	4686074	493	17.69	2.38E-70	4685806..4686215	4686046	410	34.94	9.96E-268
yadN	0	Intergenic	166932..167338	167148	407	17.61	1.12E-69	166938..167356	167187	419	46.15	0.00E+00
fimE	-4	Intergenic	5174588..5174998	5174788	411	17.60	1.28E-69	5174557..5174977	5174727	421	36.01	2.97E-284
fucA	0	Intragenic	3285938..3286459	3286138	522	17.43	2.45E-68	3285970..3286380	3286213	411	32.80	2.94E-236
CE10_4567	0	Intergenic	4657513..4657954	4657713	442	17.36	8.36E-68	4657572..4657925	4657738	354	28.57	7.83E-180
CE10_2939	0	Intergenic	2996379..2996812	2996579	434	17.32	1.72E-67	2996461..2996801	2996632	341	40.67	0.00E+00
sipD	0	Intergenic	4364806..4365170	4364944	365	17.16	2.48E-66	4365735..4366090	4365923	356	40.29	0.00E+00
yhaI	0	Intergenic	3713855..3714452	3714055	598	17.05	1.68E-65	3713910..3714266	3714080	357	41.87	0.00E+00
garP	0	Intragenic	3735491..3736089	3735691	599	17.05	1.79E-65	3735521..3735844	3735691	324	36.21	2.52E-287
CE10_2689	0	Intergenic	2734327..2734783	2734587	457	17.02	3.07E-65	2734299..2734791	2734626	493	32.61	1.40E-233
pitB	0	Intergenic	3591846..3592320	3592045	475	16.88	3.33E-64	3591924..3592289	3592120	366	37.93	3.87E-315
caiT	0	Intergenic	50113..50672	50478	560	16.84	6.57E-64	50362..50693	50524	332	33.07	4.33E-240
yegH	-1	Intergenic	2401649..2402123	2401924	475	16.75	3.00E-63	2401704..2402035	2401865	332	28.45	2.50E-178
CE10_3627	0	Intergenic	3707414..3707887	3707688	474	16.72	4.51E-63	3707478..3707905	3707736	428	36.69	5.20E-295

kpsM	-250	Intergenic	3550245..3550773	3550588	529	16.58	4.71E-62	3550359..3550819	3550650	461	28.76	3.54E-182
CE10_4613	0	Intergenic	4703675..4704137	4703869	463	16.41	8.55E-61	4703713..4704075	4703906	363	31.62	8.90E-220
sat	0	Intergenic	3517434..3517910	3517626	477	16.29	5.64E-60	3517389..3517834	3517674	446	28.84	3.14E-183
ydeP	0	Intergenic	1733125..1733538	1733339	414	16.23	1.65E-59	1733128..1733543	1733374	416	21.27	1.02E-100
focA	-42	Intergenic	994851..995268	995072	418	16.01	5.48E-58	994837..995244	995079	408	26.57	7.45E-156
ecpD	0	Intergenic	166100..166433	166301	334	15.99	7.55E-58	166101..166485	166271	385	35.68	4.73E-279
neuO	460	Intragenic	839980..840574	840375	595	15.91	2.92E-57	839682..840049	839850	368	16.64	1.84E-62
fimB	0	Intergenic	5173383..5173942	5173582	560	15.62	2.79E-55	5172313..5172781	5172613	469	28.08	9.36E-174
matA	-7	Intergenic	280087..280499	280309	413	15.57	5.70E-55	280132..280487	280302	356	24.07	2.54E-128
stpA	0	Intragenic	3150391..3150799	3150588	409	15.38	1.20E-53	3150389..3150796	3150557	408	27.91	8.81E-172
leuO	0	Intergenic	92657..93240	92857	584	15.37	1.38E-53	92696..93128	92864	433	34.98	1.97E-268
ykgE	-118	Intergenic	299238..299735	299431	498	15.35	1.88E-53	299344..299739	299582	396	25.39	1.72E-142
ycdT	0	Intergenic	1166780..1167160	1166947	381	15.27	6.48E-53	1166719..1167223	1167057	505	29.38	4.78E-190
ydeK	-11	Intergenic	1743054..1743494	1743254	441	15.17	2.64E-52	1743103..1743473	1743304	371	33.42	3.33E-245
yiaW	0	Intergenic	4217559..4218001	4217802	443	15.12	5.78E-52	4217589..4217942	4217759	354	30.06	9.29E-199
ykgI	0	Intergenic	296616..296991	296780	376	15.12	5.62E-52	296611..296965	296801	355	23.23	1.20E-119
ymgG	0	Intergenic	1355685..1356124	1355885	440	14.97	5.64E-51	1355700..1356166	1355868	467	30.42	1.60E-203
yfcV	-6	Intergenic	2769704..2770197	2769884	494	14.81	6.00E-50	2769666..2770169	2770000	504	38.75	0.00E+00
CE10_4234	0	Intragenic	4312886..4313336	4313141	451	14.75	1.57E-49	4312952..4313319	4313117	368	34.35	7.90E-259
CE10_0724	0	Intergenic	777460..777936	777740	477	14.77	1.10E-49	777542..778046	777712	505	20.03	1.50E-89
CE10_3790	0	Intergenic	3866043..3866625	3866432	583	14.46	1.02E-47	3866051..3866515	3866177	465	20.57	2.54E-94
ybcM	62	Intragenic	571029..571426	571230	398	14.45	1.17E-47	571034..571492	571201	459	24.50	6.70E-133
pagP	0	Intergenic	675200..675525	675326	326	14.45	1.18E-47	675148..675511	675275	364	27.36	4.33E-165
glgS	0	Intergenic	3646974..3647421	3647225	448	14.38	3.61E-47	3646900..3647400	3647231	501	25.77	1.02E-146
CE10_0366	0	Intragenic	403402..404000	403602	599	14.33	7.37E-47	403365..403834	403525	470	25.95	9.80E-149
yiiG	0	Intergenic	4651287..4651811	4651615	525	14.27	1.66E-46	4651381..4651749	4651582	369	33.32	1.11E-243
nanR	0	Intergenic	3824875..3825370	3825171	496	14.27	1.79E-46	3824940..3825262	3825109	323	20.93	1.35E-97

CE10_0258	0	Intergenic	283534..283987	283733	454	14.26	2.03E-46	283567..283945	283776	379	19.84	7.20E-88
yfbL	0	Intergenic	2697340..2697935	2697537	596	14.21	3.90E-46	2697375..2697816	2697545	442	33.79	1.20E-250
ttdR	270	Intragenic	3660664..3661092	3660915	429	14.14	1.03E-45	3660735..3661078	3660909	344	22.57	3.99E-113
yeaI	0	Intragenic	2088989..2089430	2089240	442	14.08	2.68E-45	2089041..2089446	2089211	406	23.53	1.11E-122
ygcG	0	Intergenic	3256217..3256779	3256399	563	14.08	2.45E-45	3256230..3256703	3256400	474	30.61	5.06E-206
yeeN	0	Intergenic	2297945..2298387	2298145	443	13.92	2.37E-44	2297960..2298355	2298198	396	23.05	7.72E-118
adiY	0	Intergenic	4937037..4937578	4937380	542	13.90	2.98E-44	4937059..4937501	4937332	443	31.35	5.50E-216
agaB	0	Intergenic	3746867..3747317	3747125	451	13.90	3.16E-44	3746875..3747245	3747078	371	28.42	5.87E-178
nanC	0	Intergenic	5172352..5172793	5172545	442	13.90	3.35E-44	5172313..5172781	5172613	469	28.08	9.36E-174
ybbW	0	Intergenic	540753..541206	540951	454	13.86	5.86E-44	540741..541217	540907	477	19.64	3.44E-86
yjfI	0	Intragenic	5028723..5029121	5028920	399	13.85	6.16E-44	5028685..5029152	5028839	468	27.64	2.05E-168
ytcA	0	Intergenic	4904147..4904575	4904347	429	13.80	1.22E-43	4904104..4904563	4904397	460	28.18	5.28E-175
yhfL	0	Intergenic	3944807..3945277	3945083	471	13.75	2.46E-43	3944846..3945244	3945014	399	39.73	0.00E+00
yiaT	0	Intergenic	4214953..4215409	4215149	457	13.69	5.93E-43	4214983..4215330	4215162	348	31.65	3.70E-220
CE10_5099	0	Intergenic	5220777..5221310	5221111	534	13.68	6.42E-43	5220623..5221088	5220791	466	30.35	1.36E-202
CE10_3982	0	Intragenic	4044927..4045465	4045270	539	13.66	9.27E-43	4045050..4045404	4045220	355	25.36	3.81E-142
iraP	0	Intergenic	379970..380442	380156	473	13.56	3.42E-42	380017..380409	380184	393	25.28	2.64E-141
ais	0	Intergenic	2677253..2677737	2677543	485	13.29	1.24E-40	2677245..2677642	2677476	398	29.25	2.37E-188
speF	-254	Intergenic	730620..731105	730906	486	13.27	1.76E-40	730699..731027	730863	329	31.01	1.83E-211
fdrA	0	Intergenic	549578..549979	549774	402	13.27	1.83E-40	549626..549978	549809	353	28.60	3.33E-180
torY	0	Intergenic	2176663..2177104	2176905	442	13.11	1.42E-39	2176728..2177090	2176896	363	37.79	9.43E-313
rpmE2	-185	Intergenic	281753..282201	282006	449	13.11	1.43E-39	281742..282113	281907	372	29.68	6.57E-194
yeiT	0	Intragenic	2539229..2539663	2539426	435	13.01	5.14E-39	2539357..2539676	2539509	320	24.17	2.29E-129
CE10_0735	-142	Intergenic	791007..791403	791214	397	13.01	5.41E-39	791122..791460	791290	339	24.07	2.71E-128
yiiE	0	Intergenic	4643638..4644071	4643838	434	12.89	2.57E-38	4643550..4644028	4643718	479	20.25	1.65E-91
gadA	0	Intergenic	4125236..4125634	4125437	399	12.85	4.55E-38	4125267..4125633	4125437	367	28.27	3.83E-176
CE10_0066	0	Intergenic	78824..79258	79024	435	12.81	7.11E-38	78777..79156	78987	380	26.07	3.96E-150

fimA	-83	Intergenic	5175568..5175996	5175763	429	12.79	9.04E-38	5175462..5175893	5175735	432	24.89	4.64E-137
CE10_1661	0	Intergenic	1692466..1693013	1692815	548	12.76	1.40E-37	1692603..1692999	1692836	397	25.35	4.86E-142
feoA	-23	Intergenic	3980886..3981285	3981088	400	12.68	3.87E-37	3980897..3981257	3981092	361	25.04	1.21E-138
siiCA	0	Intergenic	498197..498591	498397	395	12.68	3.90E-37	498178..498672	498346	495	24.97	7.15E-138
fucP	104	Intragenic	3286630..3287054	3286819	425	12.47	5.55E-36	3286611..3287057	3286781	447	29.13	6.53E-187
CE10_0335	-39	Intergenic	370082..370470	370282	389	12.39	1.41E-35	370039..370542	370209	504	25.70	5.41E-146
yghT	0	Intergenic	3589432..3589897	3589699	466	12.23	1.01E-34	3589472..3589821	3589642	350	25.96	6.43E-149
yqiG	0	Intragenic	3642370..3642917	3642523	548	12.17	2.24E-34	3642410..3642844	3642579	435	24.41	6.14E-132
CE10_4295	-781	Intragenic	4363563..4364019	4363746	457	12.03	1.26E-33	4364266..4364621	4364436	356	21.64	3.76E-104
CE10_0290	0	Intergenic	320797..321310	321125	514	11.81	1.69E-32	320918..321276	321088	359	19.66	2.39E-86
CE10_1948	0	Intragenic	1971012..1971415	1971220	404	11.69	7.04E-32	1970976..1971340	1971146	365	30.91	4.95E-210
yebN	-190	Intergenic	2123960..2124427	2124236	468	11.83	1.39E-32	2124036..2124415	2124206	380	31.37	2.78E-216
yicO	0	Intergenic	4380780..4381184	4380980	405	11.51	5.56E-31	4380813..4381260	4380983	448	29.64	2.23E-193
lfpB	0	Intergenic	4455502..4456088	4455702	587	11.65	1.21E-31	4455601..4455950	4455771	350	22.21	1.26E-109
CE10_0275	0	Intergenic	303172..303592	303358	421	11.63	1.37E-31	303137..303577	303408	441	25.51	6.99E-144
yffB	0	Intergenic	2899415..2899899	2899611	485	11.66	1.07E-31	2899399..2899841	2899673	443	22.57	4.55E-113
yfgH	0	Intergenic	2938015..2938399	2938226	385	11.34	4.07E-30	2938024..2938372	2938194	349	36.15	1.66E-286
ompN	0	Intergenic	1605353..1605864	1605666	512	11.33	4.66E-30	1605480..1605815	1605645	336	23.53	1.00E-122
yobF	-210	Intergenic	2126732..2127148	2126929	417	11.43	1.51E-30	2126788..2127160	2126958	373	23.15	7.80E-119
yjgN	0	Intergenic	5109874..5110390	5110236	517	11.39	2.24E-30	5109939..5110347	5110107	409	22.85	6.79E-116
yjeJ	0	Intergenic	4993504..4993937	4993748	434	11.34	4.27E-30	4993567..4993930	4993761	364	33.12	7.73E-241
yhbX	155	Intragenic	3778601..3779064	3778786	464	11.06	9.46E-29	3778615..3779013	3778750	399	18.26	9.12E-75
yfgF	0	Intergenic	2937368..2937800	2937603	433	11.08	8.10E-29	2937404..2937812	2937643	409	23.87	3.31E-126
yihN	0	Intergenic	4624725..4625147	4624948	423	11.16	3.25E-29	4624711..4625155	4624991	445	19.44	1.68E-84
tsx2	0	Intergenic	4700266..4700782	4700587	517	11.00	1.90E-28	4700378..4700758	4700546	381	21.88	2.16E-106
CE10_4706	0	Intergenic	4811266..4811714	4811449	449	10.90	5.86E-28	4811301..4811756	4811462	456	18.11	1.27E-73
dpiB	0	Intergenic	670579..671136	670939	558	10.83	1.18E-27	670718..671125	670968	408	26.96	1.91E-160

hipB	-478	Intragenic	1739006..1739567	1739206	562	10.69	5.88E-27	1739164..1739566	1739321	403	24.28	1.77E-130
CE10_1671	0	Intragenic	1702964..1703370	1703173	407	10.35	2.02E-25	1702938..1703343	1703105	406	23.39	2.64E-121
yihQ	0	Intergenic	4631789..4632250	4632053	462	10.37	1.76E-25	4631790..4632294	4632125	505	16.99	5.24E-65
gadB	0	Intergenic	1718702..1719278	1719096	577	10.10	2.75E-24	1718780..1719174	1718950	395	19.97	5.54E-89
yceJ	0	Intragenic	1190063..1190659	1190261	597	10.18	1.24E-24	1190104..1190554	1190255	451	14.98	4.84E-51
ydjE	741	Intragenic	2071515..2071933	2071734	419	10.16	1.47E-24	2071492..2071971	2071823	480	22.08	2.56E-108
yjfZ	0	Intragenic	5045764..5046347	5046162	584	10.07	3.90E-24	5045991..5046332	5046161	342	20.73	8.84E-96
CE10_3786	0	Intragenic	3863969..3864391	3864192	423	9.79	6.32E-23	3862491..3862827	3862644	337	26.62	1.93E-156
CE10_4709	0	Intergenic	4812986..4813474	4813276	489	9.65	2.45E-22	4813018..4813406	4813265	389	19.25	6.79E-83
CE10_3570	0	Intergenic	3639848..3640320	3640144	473	9.51	9.64E-22	3639939..3640229	3640086	291	19.77	2.45E-87
ypdI	0	Intergenic	2808050..2808461	2808249	412	9.64	2.78E-22	2808140..2808489	2808306	350	34.53	1.33E-261
yegR	0	Intergenic	2432401..2432923	2432753	523	9.43	1.99E-21	2432457..2432888	2432721	432	22.75	7.56E-115
ompT	0	Intragenic	607490..607945	607749	456	9.43	2.04E-21	607592..608028	607728	437	14.50	6.42E-48
araE	0	Intergenic	3334405..3334850	3334605	446	9.42	2.35E-21	3334471..3334797	3334626	327	13.28	1.54E-40
CE10_0264	0	Intergenic	289003..289480	289200	478	9.39	2.88E-21	289016..289498	289185	483	26.03	1.02E-149
yeiL	313	Intragenic	2560996..2561409	2561216	414	9.16	2.69E-20	2561140..2561398	2561222	259	15.07	1.36E-51
allB	0	Intragenic	542144..542568	542344	425	9.11	4.19E-20	542248..542581	542412	334	13.91	2.68E-44
yjdA	0	Intergenic	4926009..4926492	4926209	484	8.89	3.01E-19	4926068..4926402	4926254	335	23.76	4.39E-125
CE10_4222	0	Intergenic	4299036..4299530	4299227	495	7.44	5.03E-14	4298913..4299400	4299083	488	15.28	5.06E-53

Table S5. Differentially expressed genes identified by RNA-seq for NMEC wild type versus *ΔdsdC1/2* in M9 minimal media

Feature ID	Fold change	FDR p-value correction
neuO	-7.9	1.66E-20
artJ	4.32	5.31E-18
argA	2.86	2.93E-10
yqaE	-2.59	8.33E-10
metA	2.09	9.59E-09
argI	2.84	3.01E-08
ftsI	-1.97	3.37E-08
dsdC2	-86.34	8.26E-07
purF	1.94	8.26E-07
yabI	-2.26	2.52E-06
argG	2.41	1.34E-05
ydiA	-1.56	1.53E-05
CE10_1649	-2.69	2.08E-05
carA	3.14	2.14E-05
metN	1.91	3.36E-05
argE	2.04	3.36E-05
mprA	2.5	7.58E-05
metB	1.8	9.18E-05
metK	1.73	1.26E-04
metI	1.77	1.26E-04
ybfA	-1.89	1.45E-04
leuA	1.76	1.63E-04
ilvB	1.68	1.94E-04
argC	2.64	2.03E-04
ilvG	1.7	3.27E-04
metF	1.77	3.27E-04
hisJ	1.82	3.38E-04
raiA	-1.74	4.69E-04
ychH	-1.74	5.14E-04
ribB	-1.61	7.11E-04
CE10_5129	-2.33	7.19E-04
papA2	1.71	1.10E-03
CE10_3054	-1.8	1.45E-03
cysU	-1.6	3.09E-03
rplJ	1.59	3.09E-03
dsrB	-2.14	4.16E-03
ilvM	1.66	4.16E-03
CE10_4032	-1.84	4.62E-03
argH	2.01	4.62E-03

carB	1.94	4.75E-03
ybgF	1.63	5.02E-03
CE10_0837	-1.85	5.70E-03
yehW	-1.55	5.70E-03
htpG	1.52	5.70E-03
purC	1.63	6.11E-03
ytjA	-1.54	6.21E-03
pyrC	2	6.21E-03
CE10_1046	-2.06	0.01
ecnB	-1.66	0.01
ypeC	-1.65	0.01
hslU	1.58	0.01
purH	1.6	0.01
yhcO	-1.71	0.02
yqjA	-1.59	0.02
aceB	1.51	0.02
artP	1.54	0.02
pyrH	1.84	0.02
ftsB	1.92	0.02
yfiD	-1.87	0.03
scsC	-1.72	0.03
yfdY	-1.67	0.03
yfdQ	-1.64	0.03
CE10_2441	-1.64	0.03
yaiE	-1.62	0.03
murJ	-1.55	0.03
CE10_4263	-1.52	0.03
mokC	-1.51	0.03
rimM	1.51	0.03
ilvN	1.56	0.03
appA	2.14	0.03
nepI	-1.67	0.04
gadB	1.5	0.04
argB	1.96	0.04
ybiV	2.13	0.04
cedA	-4.65	0.05
ypdI	-2.79	0.05
yaiY	-2.43	0.05
mgrB	-1.76	0.05
CE10_2453	-1.66	0.05
CE10_2123	-1.6	0.05
argD	1.61	0.05
yoaB	2.89	0.05

Table S6. Differentially expressed genes identified by RNA-seq for NMEC wild type versus Δ*dsdC1/2* in M9 plus 1 mM D-ser

Feature ID	Fold change	FDR p-value correction
yjiY	21.21	2.55E-38
metF	5.9	8.68E-27
gcvP	9.5	1.11E-24
gcvH	17.14	1.87E-24
artJ	8.27	5.64E-21
gcvT	10.7	1.25E-20
zinT	14.19	2.94E-20
metB	5.32	3.64E-19
metN	4.22	3.98E-18
metA	4	7.00E-17
argC	4.85	1.07E-13
argI	5.45	6.31E-13
argH	4.99	6.02E-12
argG	4.09	2.52E-11
CE10_A40	8.32	5.15E-10
ilvB	2.99	7.11E-10
neuO	-4.57	8.56E-10
argA	4.35	1.09E-09
znuA	3.23	1.75E-09
rpmE2	7.87	2.70E-09
metL	3.05	2.70E-09
argB	3.66	1.16E-08
metI	2.88	1.25E-08
gudP	9.81	2.15E-08
ilvN	2.84	2.61E-08
metC	2.64	2.61E-08
rfbB	3.13	2.92E-08
garP	21.84	3.36E-08
CE10_2908	2.64	3.90E-08
yehX	-2.98	4.45E-08
neuB	6.93	9.96E-08
CE10_3484	7.12	1.67E-07
entD	4.62	1.67E-07
hisP	3.04	1.81E-07
yjiA	3.17	2.08E-07
hisJ	3.19	2.72E-07
rpmJ1	6.12	3.80E-07
metQ	2.5	5.03E-07
metJ	3.79	8.69E-07

CE10_2905	2.59	8.76E-07
fepA	2.56	1.24E-06
neuC	6.57	1.99E-06
neuA	4.32	1.99E-06
CE10_2889	2.57	2.61E-06
carA	2.9	2.68E-06
gudD	4.29	5.68E-06
ychH	-2.68	7.63E-06
gnsB	7.02	7.90E-06
ompF	2.59	7.97E-06
dsdC2	-74.42	1.15E-05
ilvG	2.27	1.31E-05
CE10_1649	-2.9	1.77E-05
ytjA	-2.47	1.96E-05
wzx	7.6	2.05E-05
CE10_2441	-2.68	2.84E-05
yqaE	-3.1	2.84E-05
ribB	-2.32	3.07E-05
CE10_4311	5.04	3.63E-05
rplJ	2.87	3.63E-05
sdhD	2.66	3.63E-05
CE10_2452	-2.55	4.07E-05
glnA	2.86	4.32E-05
shf	2.42	5.45E-05
yohC	-2.89	7.03E-05
yddB	2.11	8.48E-05
wbbC	7.38	8.82E-05
metK	1.99	1.14E-04
neuS	6.89	1.30E-04
gntT	2.47	1.45E-04
rpsO	2.68	1.48E-04
adk	2.58	1.74E-04
argD	2.06	1.93E-04
hisM	2.23	2.00E-04
CE10_4700	10.37	2.04E-04
kpsT	3.03	2.08E-04
zapA	2.4	2.08E-04
CE10_0707	2.15	2.08E-04
yohF	-2.56	2.08E-04
mprA	2.34	2.61E-04
yddA	2.1	2.94E-04
rpsT	3.15	2.99E-04
yeiE	-2.11	3.05E-04

ybhN	-2.38	3.14E-04
rpsQ	2.63	3.47E-04
CE10_2900	2.14	3.68E-04
CE10_5114	-2.36	3.68E-04
rplL	2.95	4.04E-04
CE10_2903	2.95	4.13E-04
CE10_C1	1.55	4.17E-04
yqiB	2.73	4.43E-04
ymgE	-2.52	4.43E-04
lrp	1.98	4.96E-04
vioA	3.71	5.23E-04
argE	2.04	5.39E-04
wbbD	10.02	5.74E-04
rplM	2.56	5.93E-04
nmpC	4.21	5.94E-04
papA2	2.46	6.43E-04
neuE	4.27	6.44E-04
CE10_0582	3.15	6.47E-04
rplA	2.44	7.87E-04
CE10_0837	-1.97	7.99E-04
atpF	2.51	8.20E-04
tyrA	-1.86	8.35E-04
amiD	-2.01	8.35E-04
ispU	2.08	9.91E-04
wbbA	6.17	1.02E-03
cdaR	3.17	1.02E-03
rplD	2.15	1.02E-03
rpsL	1.97	1.02E-03
oppA	1.94	1.02E-03
yebA	1.91	1.02E-03
ypeC	-2.19	1.02E-03
alaC	2.1	1.05E-03
gltI	2.31	1.06E-03
sdhC	2.73	1.13E-03
evgA	3.08	1.30E-03
waaT	2.31	1.30E-03
papX	3.01	1.35E-03
mqo	1.91	1.40E-03
folX	2.78	1.66E-03
CE10_5123	7.35	1.71E-03
ymgG	2.4	1.71E-03
pheA	-2.04	1.86E-03
purM	2.39	1.90E-03

potA	2.16	2.04E-03
yciG	-2.16	2.15E-03
yehW	-2.32	2.15E-03
aroF	-1.82	2.28E-03
rplR	2.06	2.30E-03
hisQ	1.92	2.34E-03
ugpC	-2.05	2.35E-03
CE10_2453	-2.38	2.84E-03
phr	-1.92	2.95E-03
rpsK	1.96	2.98E-03
ybgF	1.82	2.98E-03
CE10_4312	11.08	3.00E-03
gudX	3.26	3.06E-03
hdhA	2.39	3.06E-03
dusB	2	3.13E-03
CE10_2904	1.96	3.13E-03
CE10_A10	1.69	3.14E-03
ycaD	2.01	3.24E-03
fabB	1.8	3.24E-03
CE10_1070	2.44	3.39E-03
hisF	1.85	3.39E-03
rplW	2.48	3.41E-03
CE10_1320	5.85	3.74E-03
rpmD	2.1	3.74E-03
adhP	-2.04	3.74E-03
nlpA	1.95	3.91E-03
yfbS	-1.96	3.92E-03
rplE	1.94	4.03E-03
rpmI	2.01	4.13E-03
ynfM	-1.88	4.13E-03
mdtD	-2.06	4.13E-03
CE10_1477	-2.43	4.34E-03
CE10_1029	2.09	4.51E-03
rimM	1.9	4.67E-03
rpsU	2.3	4.70E-03
irp1	-1.89	4.75E-03
cspC	1.9	4.77E-03
ycaC	-1.89	4.77E-03
slp	-2.24	4.77E-03
rplB	1.97	4.93E-03
hisG	1.72	4.93E-03
rpsJ	2.22	5.08E-03
hdeD	-2.61	5.23E-03

rpsC	2.2	5.32E-03
purE	2.06	5.32E-03
gltK	1.87	5.32E-03
cysB	1.79	5.32E-03
CE10_3456	-2.12	5.57E-03
rpsB	1.89	5.62E-03
ydgH	1.9	5.65E-03
elaB	-1.87	6.40E-03
glcA	-2.25	6.40E-03
puuA	2.14	6.42E-03
rpsA	1.88	6.44E-03
asnA	2.05	6.71E-03
mdtC	-1.94	6.77E-03
yceA	2.07	6.84E-03
ybhB	-1.8	6.84E-03
evgS	1.98	7.01E-03
CE10_1276	-4.16	7.12E-03
CE10_1254	1.77	7.21E-03
yebV	-2.21	7.21E-03
cjrA	1.71	7.91E-03
rplK	2.05	8.10E-03
rplF	1.87	8.21E-03
atpB	1.77	8.21E-03
dps	-2.17	8.21E-03
rpmB	1.83	8.40E-03
uspB	-2.01	8.40E-03
yccJ	-1.87	8.47E-03
ydgD	-1.9	8.67E-03
gltA	1.8	8.73E-03
narZ	-2.06	8.75E-03
ilvC	2.41	8.83E-03
cpsB1	2.42	8.89E-03
yohK	-2.05	8.94E-03
rpsM	2.13	8.98E-03
CE10_4442	-2.26	9.02E-03
kpsF	1.71	9.07E-03
rplI	2.22	9.23E-03
ydiU	-1.72	9.27E-03
ychF	2.04	9.55E-03
gpt	2.33	9.57E-03
glgS	-2.25	9.57E-03
CE10_2906	2.09	9.59E-03
rplQ	1.84	9.59E-03

ackA	1.71	9.59E-03
ybhO	-1.77	9.59E-03
ynfD	-2.27	9.59E-03
ygjH	-2.98	9.59E-03
ybtU	-1.79	9.75E-03
ais	38.93	0.01
CE10_0081	33.37	0.01
CE10_0789	2.84	0.01
rfbC	2.72	0.01
CE10_1048	2.46	0.01
rfaY	2.33	0.01
CE10_2898	2.31	0.01
tsx1	2.16	0.01
virK	2.09	0.01
tig	2.07	0.01
fiu	2.04	0.01
waaW	2.04	0.01
grpE	1.99	0.01
rplX	1.98	0.01
rpsD	1.94	0.01
panD	1.93	0.01
purF	1.93	0.01
cld	1.91	0.01
hscB	1.88	0.01
rpsN	1.87	0.01
rplC	1.85	0.01
trmD	1.83	0.01
rfbA	1.82	0.01
CE10_1068	1.81	0.01
kpsE	1.77	0.01
CE10_2897	1.75	0.01
ilvM	1.75	0.01
kpsM	1.74	0.01
rpoA	1.74	0.01
secA	1.74	0.01
hisC	1.68	0.01
gdhA	1.61	0.01
CE10_B3	1.55	0.01
fliC	-1.68	0.01
irp2	-1.79	0.01
yccU	-1.79	0.01
tktB	-1.87	0.01
CE10_4162	-1.89	0.01

CE10_5153	-1.91	0.01
CE10_2449	-2.04	0.01
gabP	-2.14	0.01
CE10_5152	-2.38	0.01
CE10_5132	-2.52	0.01
narU	-2.52	0.01
CE10_1733	-3.22	0.01
ydjF	4.38	0.02
CE10_5175	3.83	0.02
spr	2.41	0.02
yedV	2.38	0.02
groS	1.87	0.02
lrhA	1.87	0.02
rpsH	1.86	0.02
atpC	1.85	0.02
rplV	1.84	0.02
CE10_1063	1.81	0.02
crp	1.81	0.02
amtB	1.8	0.02
carB	1.8	0.02
cvpA	1.8	0.02
siiCA	1.76	0.02
rplN	1.74	0.02
dtpD	1.73	0.02
sdhA	1.72	0.02
hisB	1.67	0.02
nfuA	1.66	0.02
tgt	1.66	0.02
alaA	1.65	0.02
ppiD	1.65	0.02
cjrB	1.52	0.02
ygaM	-1.66	0.02
menC	-1.68	0.02
yqjK	-1.7	0.02
wzxE	-1.71	0.02
bcsG	-1.72	0.02
yncB	-1.72	0.02
yciV	-1.73	0.02
cdd	-1.75	0.02
ybfA	-1.76	0.02
yhjG	-1.76	0.02
treF	-1.77	0.02
gabD	-1.79	0.02

patA	-1.81	0.02
ypfG	-1.82	0.02
mdlB	-1.83	0.02
ybiO	-1.88	0.02
yfiM	-1.89	0.02
yfiL	-1.9	0.02
blc2	-1.92	0.02
CE10_0551	-1.92	0.02
yddH	-2.05	0.02
yeeY	-2.05	0.02
yfgG	-2.22	0.02
CE10_2446	-2.32	0.02
ybaT	-2.32	0.02
gadA	-2.45	0.02
kdpE	-2.52	0.02
cspB	30.62	0.03
CE10_0658	4.89	0.03
mdtJ	3.42	0.03
CE10_4407	2.82	0.03
CE10_2902	2.4	0.03
papH	2.33	0.03
waaV	2.12	0.03
rpmC	2.03	0.03
coaA	1.99	0.03
cirA	1.96	0.03
rplY	1.96	0.03
rpsF	1.92	0.03
tsf	1.87	0.03
xanP	1.87	0.03
pyrC	1.82	0.03
fhuE	1.81	0.03
sdhB	1.76	0.03
lptC	1.73	0.03
hns	1.72	0.03
fusA	1.71	0.03
rpsE	1.71	0.03
znuC	1.69	0.03
hfq	1.68	0.03
hisI	1.68	0.03
modF	1.68	0.03
speE	1.66	0.03
dppA	1.65	0.03
cysP	1.63	0.03

iscA	1.62	0.03
cyoA	1.61	0.03
hisA	1.6	0.03
efeO	1.59	0.03
mug	-1.62	0.03
fic	-1.63	0.03
kefB	-1.64	0.03
aldB	-1.65	0.03
nuoL	-1.65	0.03
ydhF	-1.66	0.03
menE	-1.68	0.03
prr	-1.73	0.03
yieF	-1.74	0.03
yfdY	-1.78	0.03
sapC	-1.8	0.03
nuoN	-1.81	0.03
mtr	-1.82	0.03
yehY	-1.86	0.03
yabI	-1.91	0.03
CE10_1475	-2.07	0.03
alkB	-2.1	0.03
ybaS	-2.15	0.03
focB	-2.26	0.03
yhdV	-2.47	0.03
CE10_1020	3.28	0.04
CE10_2381	2.56	0.04
ynfC	2.37	0.04
repB	1.95	0.04
ycjY	1.94	0.04
rstA	1.86	0.04
rpsS	1.77	0.04
rpsG	1.75	0.04
rplT	1.7	0.04
yceD	1.69	0.04
aroA	1.63	0.04
pdxH	1.61	0.04
iscS	1.59	0.04
senB	1.59	0.04
CE10_3055	1.58	0.04
dapB	1.54	0.04
CE10_5036	-1.57	0.04
CE10_A16	-1.57	0.04
csrA	-1.62	0.04

ubiF	-1.62	0.04
nadB	-1.63	0.04
ybaL	-1.63	0.04
CE10_5167	-1.65	0.04
rsvB	-1.65	0.04
ybtT	-1.7	0.04
tam	-1.71	0.04
yhjD	-1.73	0.04
wrbA	-1.75	0.04
yohD	-1.82	0.04
scsC	-1.91	0.04
yebW	-2.17	0.04
astE	-2.37	0.04
gatY1	-3.16	0.04
ybcM	2.67	0.05
CE10_4737	2.49	0.05
rpsR	2.08	0.05
yqiC	1.93	0.05
iscX	1.92	0.05
CE10_4228	1.87	0.05
rsmE	1.85	0.05
manA	1.75	0.05
ompT	1.72	0.05
yciT	1.72	0.05
topA	1.68	0.05
rplS	1.64	0.05
atpH	1.63	0.05
cspA	1.62	0.05
ibeC	1.62	0.05
aceB	1.59	0.05
dhaM	1.59	0.05
accC	1.58	0.05
atpA	1.58	0.05
nusA	1.58	0.05
atpG	1.57	0.05
hisD	1.5	0.05
udp	-1.55	0.05
otsB	-1.56	0.05
mntH	-1.57	0.05
aidB	-1.58	0.05
ampE	-1.58	0.05
aroL	-1.58	0.05
yniA	-1.6	0.05

dkgA	-1.64	0.05
yebS	-1.66	0.05
yqcA	-1.71	0.05
yraQ	-1.71	0.05
pgpA	-1.77	0.05
yhcO	-1.79	0.05
yjcB	-2.02	0.05
ydcV	-2.11	0.05
flgN	-2.78	0.05
rhaB	-24.76	0.05

Table S7. Differentially expressed genes identified by RNA-seq for NMEC wild type versus *ΔdsdC1/2* + pDsdA in M9 plus 1 mM D-ser

Feature ID	Fold change	FDR p-value correction
dsdA1	99.84	5.38E-263
dsdA2	49.25	2.01E-227
yfiD	11.59	8.38E-90
neuO	-8.15	5.07E-71
ychH	5.69	6.85E-31
dps	3.19	7.99E-25
raiA	3.25	7.47E-21
yegP	3.02	1.48E-19
CE10_2893	2.77	2.72E-19
secG	-2.86	4.99E-19
sdhC	-4.1	8.37E-19
hdeA	3.58	8.52E-19
spr	-2.88	2.15E-18
atpI	-3	1.61E-17
dsdC2	-210.52	1.61E-17
ompC	2.56	2.34E-17
rnpA	-3.45	1.33E-16
neuS	3.98	2.71E-16
dcuA	2.55	2.71E-16
rpsU	-2.67	5.33E-16
hdeB	3.9	9.08E-16
yciG	2.68	4.81E-15
gapC	2.74	5.03E-15
bssR	3.3	7.21E-15
hchA	2.49	1.76E-14
yahO	2.61	2.41E-14
CE10_1649	-3.17	3.31E-14
CE10_4853	-161.47	2.44E-13
gadC	2.9	1.23E-12
adhP	2.24	1.83E-12
ycaC	2.31	2.13E-12
sdhD	-2.86	1.31E-11
sra	2.48	1.69E-11
ycgB	2.25	1.77E-11
yiaG	2.38	2.56E-11
neuC	2.31	2.56E-11
neuE	3.1	3.41E-11
CE10_3922	3.11	3.51E-11
patA	2.14	3.51E-11

dusB	-2.3	3.51E-11
infA	-2.38	3.54E-11
grxB	2.21	4.06E-11
ybgS	2.48	4.39E-11
fbaB	2.12	5.01E-11
lysP	-2.25	5.01E-11
yeaG	2.11	6.48E-11
gnsB	2.69	6.58E-11
slp	2.35	7.01E-11
gadB	2.5	7.62E-11
rpsT	-2.17	7.69E-11
yccJ	2.12	1.51E-10
osmY	2.08	1.92E-10
fis	-2.42	2.36E-10
waaV	4.25	2.40E-10
hpf	2.08	8.86E-10
tktB	2.02	1.03E-09
yjbJ	4.03	1.17E-09
suhB	-2.63	1.28E-09
osmC	2.1	1.33E-09
ybfA	2.27	1.80E-09
tomB	2.43	2.03E-09
bax	-2.16	2.03E-09
stpA	-2.2	2.10E-09
pheA	2	2.16E-09
ndh	2.09	3.16E-09
neuB	1.99	6.19E-09
adhE	1.95	6.19E-09
yeaH	2.08	6.78E-09
ygaU	2.02	7.34E-09
ytjA	2.12	8.10E-09
CE10_C2	1.55	9.45E-09
neuA	2.01	1.11E-08
msyB	1.97	1.29E-08
yeeF	-2.09	1.30E-08
fabA	-2.07	1.92E-08
gadX	2.01	2.94E-08
dadA	2.48	3.79E-08
irp2	1.99	5.37E-08
elaB	1.9	9.09E-08
apt	-2.15	9.09E-08
yghA	2.23	1.10E-07
ecnB	1.92	1.31E-07

vioA	2.25	1.48E-07
ndk	-2.07	1.56E-07
mltD	-1.88	1.59E-07
irp1	1.96	1.71E-07
wrbA	1.86	1.82E-07
xanP	-1.92	2.13E-07
ydcK	2.27	2.45E-07
yceK	1.95	2.64E-07
ybaS	2.23	2.68E-07
uspD	1.98	2.76E-07
yebV	1.96	3.16E-07
cspE	-1.83	3.23E-07
gmr	1.93	4.37E-07
rlmH	-2.45	4.66E-07
gabD	1.85	4.88E-07
bolA	1.89	5.12E-07
sixA	-1.9	5.97E-07
poxB	1.81	6.09E-07
mdtJ	-11.2	7.77E-07
aceE	1.78	7.98E-07
CE10_5036	1.81	8.05E-07
yeaQ	1.89	8.08E-07
lpxB	-1.92	8.64E-07
argC	-1.94	8.64E-07
rpmB	-1.81	8.81E-07
entD	-2.1	8.81E-07
aceF	1.77	9.45E-07
yohC	2.55	9.61E-07
CE10_0523	3.76	1.02E-06
psiF	2.3	1.06E-06
ybhB	1.9	1.06E-06
gcd	1.78	1.11E-06
arnB	-2.05	1.15E-06
yhcO	2.28	1.16E-06
yaeH	1.94	1.30E-06
atpB	-1.79	1.32E-06
uspB	2.04	1.39E-06
treF	1.89	1.44E-06
ykgC	2.13	1.46E-06
yeaZ	-2.24	1.46E-06
CE10_1097	-1.79	1.77E-06
fic	1.87	2.15E-06
otsA	1.79	2.32E-06

deoB	1.79	2.35E-06
rpmH	-2.21	2.59E-06
panD	-1.86	2.75E-06
CE10_0522	1.98	2.93E-06
ydhS	1.95	3.06E-06
asnA	-1.86	3.29E-06
yajG	-1.94	3.29E-06
CE10_4652	-4.54	4.63E-06
aidB	1.81	5.00E-06
CE10_1313	2.59	5.61E-06
CE10_3484	2.54	6.15E-06
mqsA	2.18	6.79E-06
iucD	1.7	6.79E-06
cirA	-1.73	6.81E-06
yfdY	2.27	7.27E-06
cdd	2.03	7.42E-06
deoA	1.87	8.44E-06
mreC	-1.93	9.07E-06
gpt	-2.01	9.39E-06
ymgE	3	9.67E-06
gabP	1.98	9.74E-06
yhhA	2.53	1.04E-05
glgS	2.11	1.04E-05
cbpM	2.15	1.05E-05
waaT	2.06	1.13E-05
cspA	-3.95	1.16E-05
osmB	2.11	1.38E-05
uspF	1.83	1.38E-05
ubiX	-2.35	1.61E-05
mltA	-1.84	2.02E-05
luxS	1.68	2.08E-05
uspE	1.7	2.09E-05
leuC	1.66	2.35E-05
wzy	3.22	2.44E-05
ispA	-1.85	2.52E-05
gadA	2.04	2.70E-05
cfa	1.67	2.70E-05
lysC	-1.66	2.70E-05
adk	-1.72	2.85E-05
CE10_2482	2.1	3.01E-05
aqpZ	1.82	3.22E-05
ampG	-2.08	3.22E-05
tatC	-1.84	3.28E-05

agp	1.69	3.49E-05
uspG	1.7	3.60E-05
metB	1.7	3.93E-05
ygaM	1.68	3.95E-05
ompX	1.64	4.07E-05
sdhA	-1.68	4.28E-05
argA	-1.71	4.28E-05
ybaT	2.1	4.69E-05
gabT	1.67	4.77E-05
CE10_0428	2.68	4.80E-05
purE	-1.76	4.91E-05
rsxB	-2.23	4.91E-05
dkgA	1.72	5.03E-05
phoH	1.66	5.05E-05
mdtI	-6.41	5.19E-05
deoC	1.92	5.76E-05
ppiA	-1.78	6.16E-05
ydiY	-2.46	6.21E-05
plsX	-1.71	6.55E-05
CE10_0262	1.87	6.64E-05
fruB	1.89	7.13E-05
leuD	1.62	7.53E-05
CE10_0837	2.19	8.21E-05
yahK	1.65	8.30E-05
talA	1.63	9.44E-05
psiE	2.15	9.70E-05
yhbY	-1.79	1.01E-04
ydhR	1.86	1.02E-04
yqjD	1.63	1.02E-04
kpsT	1.66	1.04E-04
exuR	1.75	1.06E-04
yceD	-1.61	1.08E-04
aroK	-1.66	1.11E-04
envC	-1.76	1.11E-04
queA	-2.05	1.11E-04
amyA	1.61	1.15E-04
hofQ	-8.45	1.20E-04
yjcD	-1.64	1.28E-04
yjdJ	2.07	1.31E-04
gadE	16.68	1.45E-04
potA	-1.69	1.51E-04
qorA	1.67	1.64E-04
accA	-1.63	1.64E-04

metQ	1.59	1.65E-04
waaW	1.88	1.67E-04
ybjN	-1.85	1.67E-04
ydiZ	1.72	1.69E-04
ivy	1.63	1.71E-04
rzpD	-2.75	1.90E-04
qmcA	1.73	1.93E-04
ybhL	1.65	1.96E-04
ybeL	1.66	2.04E-04
aldB	1.68	2.10E-04
tsx1	-1.74	2.13E-04
ybaY	1.64	2.15E-04
ybgC	-1.78	2.16E-04
ycaO	-1.72	2.23E-04
pflB	1.57	2.26E-04
kbl	1.67	2.28E-04
leuB	1.57	2.28E-04
ygdI	2.15	2.34E-04
plsY	-2.06	2.44E-04
CE10_1314	1.79	2.53E-04
ldhA	1.66	2.53E-04
yqeF	-1.8	2.53E-04
ygaH	-2.02	2.53E-04
fiu	-1.62	2.57E-04
ynhG	1.64	2.62E-04
CE10_0706	-1.94	2.62E-04
katE	1.64	2.72E-04
gloB	1.81	2.75E-04
tonB	-1.61	2.82E-04
mioC	-1.65	2.82E-04
yniC	-1.77	2.82E-04
yiiS	1.93	2.85E-04
rfaI	1.73	3.02E-04
fxsA	-2.2	3.06E-04
yehS	-2.35	3.14E-04
yajO	1.67	3.22E-04
mscS	1.56	3.22E-04
yjcC	1.82	3.30E-04
fyuA	1.68	3.30E-04
rplU	-1.58	3.30E-04
ydfH	-1.79	3.36E-04
accD	-1.57	3.68E-04
yoaC	1.94	3.75E-04

bamD	-1.59	3.79E-04
wzx	2.29	3.87E-04
purM	-1.58	3.88E-04
dadX	2.15	4.08E-04
mqsR	2.15	4.14E-04
exbB	-1.56	4.34E-04
purN	-1.62	4.40E-04
yceA	-1.67	4.44E-04
mscL	1.56	4.45E-04
speD	-1.59	4.48E-04
hdeD	2.38	4.50E-04
yqjG	1.66	4.81E-04
modF	-1.57	4.93E-04
CE10_0263	1.93	4.99E-04
pheP	-1.82	4.99E-04
glnA	-1.54	5.13E-04
carA	-1.57	5.17E-04
phnB	2.18	5.59E-04
pepT	1.58	5.59E-04
yqjE	1.56	5.59E-04
holD	-2.08	5.59E-04
yncB	1.61	5.76E-04
metL	1.55	6.10E-04
uspC	1.83	6.28E-04
hmp	1.68	6.28E-04
crr	1.53	6.36E-04
dppA	1.53	6.36E-04
rplJ	-1.53	6.36E-04
rplY	-1.58	6.52E-04
glpD	1.85	6.83E-04
yhjG	1.61	7.00E-04
rsxA	-1.92	7.00E-04
ybjG	-1.91	7.40E-04
yigI	-1.9	7.67E-04
rsmD	-1.92	7.67E-04
yieF	1.6	7.72E-04
yggL	1.59	7.72E-04
yniA	1.59	7.72E-04
iutA	1.51	8.10E-04
yciA	-1.91	8.80E-04
yidB	1.66	8.84E-04
wbbB	2.92	9.05E-04
nanM	1.66	9.09E-04

ybhK	-1.84	9.09E-04
yejG	-1.88	9.17E-04
gss	1.55	9.27E-04
rplL	-1.51	9.32E-04
fepE	-2.1	9.44E-04
yibT	1.82	9.59E-04
secE	-1.58	9.59E-04
CE10_5090	7.74	9.68E-04
secD	-1.53	9.95E-04
ytfP	-1.61	9.95E-04
aroF	1.5	1.02E-03
iscR	-1.53	1.03E-03
alaA	-1.57	1.03E-03
slmA	-1.74	1.03E-03
rsmG	-2.11	1.03E-03
yfdQ	1.68	1.05E-03
yfdP	2.01	1.06E-03
rfe	-1.62	1.07E-03
accB	-1.53	1.13E-03
nuoA	-1.67	1.13E-03
pth	-1.84	1.15E-03
rimO	-1.69	1.17E-03
tolQ	-1.6	1.23E-03
serB	-1.66	1.26E-03
wbbC	2.12	1.35E-03
amn	1.54	1.35E-03
prfA	-1.76	1.35E-03
hisM	-1.6	1.48E-03
tam	1.67	1.49E-03
sufD	1.5	1.54E-03
ybgT	1.79	1.60E-03
chuU	-1.54	1.71E-03
kdsC	-1.62	1.71E-03
repA	-1.54	1.90E-03
copA	1.56	1.98E-03
yiaU	2.03	2.11E-03
purR	-1.53	2.11E-03
malG	1.96	2.12E-03
CE10_1784	3.38	2.17E-03
udp	1.5	2.17E-03
fadL	-1.65	2.18E-03
repB	1.59	2.19E-03
prrA	1.6	2.24E-03

argB	-1.55	2.25E-03
mreD	-1.78	2.29E-03
dsdC1	-36.41	2.32E-03
dapB	-1.51	2.35E-03
ybjE	-1.82	2.40E-03
cvpA	-1.52	2.44E-03
rpmG	-1.52	2.44E-03
rnhB	-1.75	2.44E-03
papA2	1.57	2.50E-03
rph	-1.68	2.51E-03
ldcC	1.53	2.52E-03
cdsA	-1.54	2.54E-03
nudG	-2.31	2.58E-03
cydB	1.5	2.59E-03
ydgD	1.58	2.63E-03
prmC	-2	2.66E-03
potB	-1.62	2.70E-03
rfaL	1.92	2.73E-03
ybtS	1.64	2.73E-03
ydjN	-1.52	2.79E-03
rsuA	-1.65	2.82E-03
yafK	-1.59	2.91E-03
ybdK	1.59	2.95E-03
ybjQ	1.65	2.96E-03
agaI	-2.6	2.96E-03
CE10_3923	1.92	3.07E-03
hdhA	1.64	3.07E-03
menA	-1.72	3.09E-03
CE10_1462	1.6	3.17E-03
glcC	-2.07	3.22E-03
phoE	-2.39	3.22E-03
yccM	-2.93	3.25E-03
holE	-2.74	3.30E-03
ydeI	2.09	3.31E-03
mtr	1.62	3.33E-03
hslV	-1.64	3.58E-03
mlaE	-1.57	3.75E-03
rluC	-1.64	3.75E-03
bssS	1.99	3.76E-03
yjgR	1.5	3.76E-03
pldA	-1.67	3.76E-03
treA	1.51	3.77E-03
ygiQ	-1.75	3.93E-03

CE10_4266	-1.67	4.19E-03
dctA	-1.87	4.27E-03
yacL	1.62	4.36E-03
otsB	1.5	4.41E-03
ddpX	-2.98	4.69E-03
lhgO	1.53	4.78E-03
tdh	1.5	4.83E-03
CE10_2891	-2.67	5.10E-03
CE10_2919	5.67	5.15E-03
ybiB	1.5	5.21E-03
csiD	1.52	5.34E-03
holA	-1.5	5.66E-03
yheA	-1.6	5.66E-03
yrbA	-1.67	5.66E-03
trmJ	-1.6	5.73E-03
yjbQ	1.58	5.74E-03
ubiE	-1.52	5.75E-03
glpE	-1.98	5.91E-03
ybiI	1.77	5.92E-03
hicB	1.71	5.92E-03
yqaA	-1.74	5.95E-03
eptA	-1.59	6.06E-03
ygjR	1.61	6.14E-03
yjbE	4.53	6.27E-03
ygaZ	-1.52	6.45E-03
yfcC	-4.07	6.55E-03
ypjD	-1.61	6.66E-03
lipA	1.5	6.68E-03
yifK	-1.55	6.76E-03
ydcI	-1.54	6.80E-03
CE10_4268	1.59	7.09E-03
ybeB	-1.6	7.28E-03
fadR	-1.59	7.49E-03
deoR	-1.56	7.77E-03
gadW	1.52	7.99E-03
plsC	-1.65	8.07E-03
CE10_1029	1.51	8.11E-03
yjaH	-1.66	8.26E-03
yoaA	-1.55	8.34E-03
rfaY	1.73	8.36E-03
nudE	-1.52	8.55E-03
alx	-2.73	8.71E-03
ompW	2.05	9.20E-03

amiA	-1.6	9.57E-03
aldA	-1.63	9.85E-03
CE10_3406	1.7	9.98E-03
yodD	1.69	9.98E-03
yhcN	2.04	0.01
sorC	1.95	0.01
yhfG	1.85	0.01
yhdZ	1.82	0.01
yjiY	1.82	0.01
ymdB	1.71	0.01
dkgB	1.66	0.01
ypfH	1.58	0.01
ygdR	1.57	0.01
dosP	1.52	0.01
yccU	1.5	0.01
fdhE	-1.5	0.01
recQ	-1.5	0.01
kdgT	-1.62	0.01
CE10_4738	-1.77	0.01
rnk	-1.78	0.01
yfeZ	-1.85	0.01
ytfF	-1.99	0.01
CE10_1214	-2.04	0.01
yahB	-2.08	0.01
ais	-2.14	0.01
uhpA	-2.33	0.01
pspB	2.55	0.02
yibI	2.4	0.02
ygbA	2.28	0.02
CE10_1278	2.16	0.02
wbbA	1.98	0.02
wbbD	1.98	0.02
CE10_0373	1.97	0.02
ybtE	1.74	0.02
gudD	1.68	0.02
sohA	1.64	0.02
yafC	1.59	0.02
uxaB	1.57	0.02
CE10_4667	1.54	0.02
glpQ	1.54	0.02
nrdD	1.51	0.02
ugpC	1.51	0.02
cbl	-1.52	0.02

clpS	-1.53	0.02
lipB	-1.53	0.02
pyrF	-1.53	0.02
cyaY	-1.55	0.02
yhaJ	-1.55	0.02
rluF	-1.56	0.02
ydeK	-1.57	0.02
ascG	-1.58	0.02
rtn	-1.59	0.02
trmA	-1.63	0.02
yjgM	-1.64	0.02
yecJ	-1.66	0.02
rumB	-1.7	0.02
yjiJ	-1.71	0.02
nohA1	-1.72	0.02
CE10_1648	-1.73	0.02
dusC	-1.73	0.02
yobD	-1.82	0.02
CE10_2123	-1.83	0.02
CE10_1767	-1.89	0.02
CE10_3461	-1.95	0.02
CE10_2510	-2	0.02
yiaD	-2.19	0.02
ymfQ	-5.45	0.02
CE10_1978	2.29	0.03
CE10_2740	2.2	0.03
mcbA	2.04	0.03
yeeD	1.89	0.03
CE10_1034	1.8	0.03
bhsA	1.74	0.03
yqaE	1.73	0.03
nhoA	1.63	0.03
CE10_1448	1.62	0.03
CE10_3624	1.58	0.03
CE10_1441	1.56	0.03
yodC	1.51	0.03
recF	-1.5	0.03
ucpA	-1.5	0.03
mglB	-1.55	0.03
glpX	-1.58	0.03
rarD	-1.58	0.03
yjeJ	-1.58	0.03
xseB	-1.59	0.03

yehU	-1.59	0.03
yacC	-1.66	0.03
yiiQ	-1.67	0.03
rfaH	-1.71	0.03
cdaR	-1.88	0.03
sanA	-1.89	0.03
cedA	-2.15	0.03
yqeA	2.12	0.04
eco	1.83	0.04
CE10_4480	1.78	0.04
melR	1.73	0.04
CE10_1315	1.72	0.04
adrB	1.68	0.04
hsdS	1.55	0.04
CE10_3456	1.53	0.04
codB	-1.53	0.04
yeiP	-1.53	0.04
yhbE	-1.53	0.04
ylaC	-1.57	0.04
hcaR	-1.59	0.04
ybhQ	-1.65	0.04
yaeB	-1.66	0.04
tatD	-1.68	0.04
dinF	-1.74	0.04
rnt	-1.74	0.04
cdh	-1.81	0.04
CE10_2481	-2.38	0.04
xylF	-3.99	0.04
papE1	9.39	0.05
CE10_1025	2.64	0.05
quuQ	2.27	0.05
gatY1	2.11	0.05
csgE	1.75	0.05
frwD	1.64	0.05
lsrK	1.63	0.05
yidX	1.63	0.05
hcaT	-1.63	0.05
yjeA	-1.65	0.05
cynR	-2	0.05
gspO	-2.99	0.05

Table S8. Common differentially expressed genes between NMEC vs Δ*dsdC1/2* and NMEC vs Δ*dsdC1/2* + pDsdA in the presence of D-ser.

	**Condition 2 CE10 WT$^{D\text{-}ser}$ vs. ΔΔ*dsdC1/2*$^{D\text{-}ser}$**		**Condition 3 CE10 WT$^{D\text{-}ser}$ vs. ΔΔ*dsdC1/2* + pDsdA1/2$^{D\text{-}ser}$**	
	**Fold change**	**FDR *p*-value**	**Fold change**	**FDR *p*-value**
kpsT	3.03	2.08E-04	1.66	1.04E-04
neuA	4.32	1.99E-06	2.01	1.11E-08
neuB	6.93	9.96E-08	1.99	6.19E-09
neuE	4.27	6.44E-04	3.1	3.41E-11
neuC	6.57	1.99E-06	2.31	2.56E-11
neuS	6.89	1.30E-04	3.98	2.71E-16
neuO	-4.57	8.56E-10	-8.15	5.07E-71
papA2	2.46	6.43E-04	1.57	2.50E-03
metQ	1.59	1.65E-04	2.5	5.03E-07
wbbD	10.02	5.74E-04	1.98	2.00E-02
wbbA	6.17	1.02E-03	1.98	2.00E-02
rfaY	2.33	1.00E-02	1.73	8.36E-03
wbbC	7.38	8.82E-05	2.12	1.35E-03
wzx	7.6	2.05E-05	2.29	3.87E-04
waaW	2.04	1.00E-02	1.88	1.67E-04
waaT	2.31	1.30E-03	2.06	1.13E-05
vioA	3.71	5.23E-04	2.25	1.48E-07
waaV	2.12	3.00E-02	4.25	2.40E-10
gudD	4.29	5.68E-06	1.68	2.00E-02
hdhA	2.39	3.06E-03	1.64	3.07E-03
metL	3.05	2.70E-09	1.55	6.10E-04
metB	5.32	3.64E-19	1.7	3.93E-05
CE10_1649	-2.9	1.77E-05	-3.17	3.31E-14
repB	1.95	4.00E-02	1.59	2.19E-03
gnsB	7.02	7.90E-06	2.69	6.58E-11
dsdC2	-74.42	1.15E-05	-210.52	1.61E-17
yjiY	21.21	2.55E-38	1.82	1.00E-02
dppA	1.65	3.00E-02	1.53	6.36E-04
CE10_1029	2.09	4.51E-03	1.51	8.11E-03
CE10_3484	7.12	1.67E-07	2.54	6.15E-06

*Genes that had an increase in fold change values are shown in green, and genes that had a decrease in red

Table S9. *neuO* co-ordinates and correlation with *dsdCXA* presence and location

Strain	Accession	***neuO*** **co-ordinates**[*]	***neuO*** **pseudogene**[†]	***neuO*** **location**	***dsdCXA*** **co-ordinates**[‡]	**Proximity** ***neuO-dsdCXA***[§]
CE10	CP003034.1	839544-840320<br>2782259-2782020		*pgl-bioA*<br>*argW-dsdC*	2790372-2794223<br>4331432-4335252	8352
GN02175	CP041550.1	2307786-2308559		*argW-dsdC*	201050-204869<br>2267175-2263350	40611
O16:H6 strain CU37RT-2	CP082774.1	3463855-3463082		*argW-dsdC*	514697-510877<br>3504551-3508376	40696
FDAARGOS 1375	CP077389.1	4993652-4994425		*argW-dsdC*	2895732-2899552<br>4952837-4949012	40815
FDAARGOS 1374	CP077314.1	3041646-3040873		*argW-dsdC*	22631-18811<br>3082461-3086286	40815
APEC IMT5155	CP005930.1	493342-492566		*argW-dsdC*	533945-537770	40603
elppa4	CP083512.1	4135038-4135811		*thrW-paoD*	1386193-<br>1387752[#]	2748845
FDAARGOS_1304	CP069990.1	236378-237154		*argW-dsdC*	197310-193485	39068
DE17	CP045206.1	1523964-1524740		*argW-dsdC*	1483361-1479536	40603
RHB33-C14	CP057192.1	3729709-3728936		*thrW-ecpE*	1527546-1523695	2201390
SCU-124	CP051706.1	1482526-1483299		*argW-dsdC*	1433033-1429182	49493
SCU-123	CP051711.1	1209629-1208856	Y	*argW-dsdC*	1248819-1252644	39190
SCU-108	CP051735.1	1209621-1208848		*argW-dsdC*	1248741-1252566	39120
PA45B	CP021288.1	2556421-2555648	Y	*argW-dsdC*	2595506-2599331	39085
A42	CP028734.1	1524070-1524843		*argW-dsdC*	1474543-1470692	49527
F17EC0245	CP088874.1	1616350-1617123		*argW-dsdA*	1571230-<br>1569664[#]	45120
18MD05VL07 005213EC	CP063729.1	3314500-3313727<br>3888131-3887358	Y	*pgl-bioA*<br>*thrW-paoD*	1502911-<br>1501352[#]	1810816
EC931	CP049118.1	4218269-4219042	Y	*pgl-bioA*	1278887-1282712	2031015
SCU-488	CP054449.1	3741980-3742756	Y	*argW-dsdC*	3704019-3700197	37961
SCU-116	CP051719.1	1240022-1239249	Y	*argW-dsdC*	1250251-1254076	10229
EC28	CP049101.1	1571460-1572233	Y	*argW-dsdC*	1519803-1515952	51657
BEN2908	LR740776.1	2531319-2530546	Y	*argW-dsdC*	2570588-257441	39269
PU-1	CP042246.1	3379957-3379184<br>1617782-1618483	Y	*pgl-bioA*<br>*argW-dsdC*	1579808-1575983	37974
GN03409	CP041535.1	1822232-1821459	Y	*argW-dsdC*	4068757-4064938<br>1860603-1864428	38371
GN05696	CP041526.1	4108193-4108966		*argW-dsdC*	4090643-4086818	17550
NCTC11151	LR134031.1	1578165-1578938	NG	*argW-dsdC*	1537457-1533633	40708
DSM 30083	CP033092.2	1563146-1563919	Y	*argW-dsdC*	1522504-1518679	40642
MS7163	CP026853.1	2535240-2534467		*argW-dsdC*	2575725-2579550	40485
MS8345	CP025401.1	2544386-2543613		*argW-dsdC*	2566493-2570318	22107
ExPEC XM	CP025328.1	845960-846733<br>866476-867249<br>2628638-2627923	Y	*pgl-bioA*<br>*pgl-bioA*<br>*argW-dsdC*	2666611-2670436	37973
NU14	CP019777.1	2610579-2609806	Y	*argW-dsdC*	4875832-4872013<br>2648852-2652677	38273
G749	CP014488.1	2457980-2457207	Y	*argW-dsdC*	2507639-2511464	49659
SF-173	CP012631.1	1507629-1508402	Y	*argW-dsdC*	4300623-4304436<br>1469237-1465412	38392
SF-088	CP012635.1	1494417-1495190	Y	*argW-dsdC*	1483880-1480055	10537
NMEC O18	CP007275.1	1758634-1757858		*argW-emrB*		
APEC O18	CP006830.1	1468028-1467252		*argW-dsdC*	1506284-1510109	38256
RS218	CP007149.1	2618822-2618049	NG	*argW-dsdC*	4927657-4923838<br>2657092-2660917	38270

EC9682	CP095271.1	1508456-1509229		*argW-dsdC*	4406882-4410695<br>1470120-1466295	38336
W224N	CP094339.1	1557492-1558265	GNA	*argW-dsdC*	4384104-4387917	2825839
B16EC0986	CP088740.1	3392169-3391396		*argW-dsdC*	3401279-3405104	9110
C16EC0488	CP088662.1	2436200-2435427		*argW-dsdC*	2474292-2478117	38092
F16EC0267	CP088421.1	1653333-1654106		*argW-dsdC*	1615124-1611299	38209
PMV-1	HG428755.1	2602625-2601852		*argW-dsdC*	2640898-2644723	38273
FDAARGOS 1386	CP077294.1	284621-283848	Y	*argW-dsdC*	322642-326467	38021
FDAARGOS_1297	CP070148.1	3155997-3155224	Y	*argW-dsdC*	391234-387415<br>3196348-3200173	40351
FDAARGOS_1298	CP070115.1	3232928-3233701	Y	*argW-dsdC*	3194816-3190991	38112
FDAARGOS_1303	CP070045.1	2658428-2657655	Y	*argW-dsdC*	4968287-4964468<br>2696869-2700694	38441
FDAARGOS_1300	CP069996.1	3881183-3881956		*argW-dsdC*	3856273-3852448	24910
FDAARGOS_1299	CP069978.1	308284-309057	Y	*argW-dsdC*	3111629-3115442<br>269955-266130	38329
FDAARGOS_1295	CP069890.1	226643-225870	Y	*argW-dsdC*	2537970-2534151<br>267078-270903	40435
UTI89	CP064825.1	2599703-2598930	Y	*argW-dsdC*	4864958-4861139<br>2637976-2641801	38273
RHBSTW-00087	CP056867.1	1653228-1654001		*argW-dsdC*	1614908-1611083	38320
S88	CU928161.2	2495525-2494752	Y	*argW-dsdC*	2533608-2537433	38083
APEC O1	CP000468.1	2572393-2571620		*argW-dsdC*	2610513-2614338	38120
SCU-487	CP054454.1	1851069-1851844	Y	*argW-dsdC*	1840702-1836877	10367
A16EC0054	CP088869.1	3546003-3545244		*argW-dsdC*	3585060-3585886<br>1660213-1656388	39057
UM146	CP002167.1	1042712-1043484	NG	*argW-dsdC*	4606222-4602404<br>1004442-1000617	38270
ST95-32	CP043950.1	3518985-3518235		*argW-dsdC*	3556958-3560783	37973
SCU-175	CP054379.1	3638704-3637985<br>1611195-1611434		*pgl-bioA*<br>*argW-dsdC*	1603082-1599231<br>94008-90188	8352
IHE3034	CP001969.1	2714949-2714213	Y	*argW-dsdC*	2753210-2757035	38261
Es_ST80_L1_NDM_10_2017	CP031215.1	1981-2712		*argW-dsdA*	5173589-<br>5172023#	43104
C5	CP072911.1	3476602-3475880	Y	*argW-dsdC*	703851-700032<br>3516815-3520637	40213
FDAARGOS_1301	CP070111.1	31949-32671	Y	*argW-dsdC*	16228-12403	15721
SCAID URN1-2021	CP082824.1	1541545-154082<br>1606859-1607576		*argW-dsdA*	1499405-<br>1497839#	42140
SF-468	CP012625.1	1650449-1651164	Y	*argW-dsdC*	1612474-1608649	37975
4928STDY7071353	LR607328.1	1283877-1284585	GNA	*argW*	4930869-4929009<br>2670259-2671681	1385674
ZJ3920	CP020545.1	2439099-2438391		*argW-dsdC*	2477073-2480898	37974
FHI99	LM997272.1	301901-301193	GNA	*argW-dsdA*	263024-261516#	38877
BEN5202	LR740758.1	2468105-2467404		*argW-dsdC*	2502717-2506542	34612
elppa1	CP083534.1	4032577-4031876		*argW-dsdC*	4070552-4074377	37975
IAI39	CU928164.2	789059-789760<br>2590371-2590132	Y	*argW-dsdC*	2598484-2602335<br>4390042-4393862	8113
SP15 DNA	AP024131.1	1710899-1710205	NG	*argW-dsdC*	1749160-1752985	38261

FDAARGOS_1302	CP069935.1	2115282-2114588	Y	*argW-dsdC*	2126115-2129940	10833
UPEC129	CP071522.1	1563995-1564677	Y	*argW-dsdC*	1524924-1521099	39071
HS13-1	CP026491.1	1223176-1222496	Y	*argW-dsdC*	1261152-1264977	37976
FDAARGOS_1296	CP070066.1	1628893-1629559		*argW-dsdC*	1587143-1583318	41750
AR_0013	CP032204.1	2928440-2928679		*argW-dsdC*	2918057-2914206<br>1403323-1399503	10383
RHBSTW-00176	CP056800.1	1612434-1612673		*argW-dsdC*	1602050-1598199<br>94036-90216	10384

[*]Co-ordinates obtained by BLASTn comparison with CE10 *neuO*; [†]*neuO* is annotated as a pseudogene (Y), no gene annotated in region with similarity to CE10 *neuO* (NG), genome not annotated (GNA); [‡]Co-ordinates obtained by BLASTn comparison with CE10 *dsdCXA1*, [#]indicates broken *dsd* loci lacking *dsdC* and the 5' end of *dsdX*; [§]Proximity (bp) of *neuO* to nearest *dsd* locus

Table S10. Bacterial strains used in this study

Strain	Characteristics	Source
CE10	Wild type NMEC O7:K1	Lu *et al.*, 2011
DsdC1^{FLAG}	NMEC strain with FLAG-tag fused to the *dsdC1* gene	This study
Δ*dsdC1*	NMEC *dsdC1* knockout	This study
DsdC2^{FLAG}	NMEC strain with FLAG-tag fused to the *dsdC2* gene	This study
Δ*dsdC2*	NMEC *dsdC2* knockout	This study
Δ*dsdC1/2*	NMEC *dsdC1/dsdC2* knockout	This study
Δ*neuO*	NMEC *neuO* knockout	This study
DH5a	Commercial *E. coli* strain	Invitrogen
BL21 DE3	Commercial *E. coli* strain	Invitrogen

Table S11. Plasmids used in this study

Plasmid	Characteristics	Source
pET28a	Plasmid for overexpression of N-terminal 6xHistidine tagged proteins (Kan^R)	Roe lab inventory
pET28_*dsdC1*	HIS-tag overexpression plasmid with CE10 derived *dsdC1* inserted between *Nde*I and XhoI (Kan^R)	This study
pET28_*dsdC2*	HIS-tag overexpression plasmid with CE10 derived *dsdC2* inserted between *Nde*I and XhoI (Kan^R)	This study
pSR	Plasmid used for DNase I foot-printing (Amp^R)	Dr Douglas Browning
pSR_*dsdCX1*	pSR with CE10 derived *dsdCX1* intergenic region inserted between *EcoR*I and *Hind*III (Amp^R)	This study
pKD3	Template plasmid for Lambda Red mutagenesis (Kan^R)	Datsenko and Wanner., 2000.
pKD4	Template plasmid for Lambda Red mutagenesis (Cm^R)	Datsenko and Wanner., 2000.
pKD46	Lambda Red recombinase expressing plasmid (Amp^R)	Datsenko and Wanner., 2000.
pACYC184	Multicopy plasmid (Cm^R)	Roe lab inventory
pDsdC1	pACYC184 with CE10 derived *dsdC1* + promoter (Cm^R)	This study
pDsdC2	pACYC184 with CE10 derived *dsdC2* + promoter (Cm^R)	This study
pNeuO	pACYC184 with CE10 derived *neuO* fused to the *gapA* promoter (Cm^R)	This study
pDsdA	pACYC184 with CFT073 derived *dsdC* (Cm^R)	Prof Rodney Welch
p*dsdXA*-GFP	pUA66 with the *dsdXA* promoter fused to GFP (Kan^R)	Zaslaver *et al.,* 2006

Table S12. Primers used in this study

Primer name	Description	Sequence
DsdC_FLAG_CHECK_F	Forward check for DsdC1/2-FLAG tag	GTTTGGCGATATGACGGTGA
DsdC1_FLAG_CHECK_R1	Reverse check for CE10 DsdC1-FLAG tag	TTCGACGCCAAGAAGATGTG
DsdC2_FLAG_CHECK_R2	Reverse check for CE10 DsdC2-FLAG tag	CGACTCCGGTACATACGACA
EMSA_dsdC_CE10_F	Forward for the ChIP peak region for EMSA for *dsdXA promoter*	CGTGCTCACAACCCAGATTT
EMSA_dsdC_CE10_R	Reverse for the ChIP peak region for EMSA for *dsdXA promoter*	CCTGGCTGCCACTTCAAAAG
EMSA_neuO_CE10_F	Forward for the ChIP peak region for EMSA *neuO* 3' end	CATTATATCTAGTTACGTATGGGTAGG
EMSA_neuO_CE10_R	Reverse for the ChIP peak region for EMSA *neuO* 3' end	GATGTTTTATATTTATTGCGTGAG
EMSA_pneuO_CE10_F	Forward for the *neuO* promoter region	GCAGCGCAGAGAAATGGATA
EMSA_pneuO_CE10_R	Reverse for the *neuO* promoter region	TCAATGGAAAACGAGTCTTGAGT
EMSA_adhE_CE10_F	Forward for the adhE gene	CTGGTACAGGTTCTGAAGTCAC
EMSA_adhE_CE10_R	Reverse for the adhE gene	CAGATATTCTTTCAGCAGTTTCAG
gapA_qPCR_F	Forward for *gapA* RT-qPCR	TTTCCGTGCTGCTCAGAAAC
gapA_qPCR_R	Reverse for *gapA* RT-qPCR	GGCCGTGAGTGGAGTCATAT
pSR_check_F	Forward check pSR insertion	CCGAAAAGTGCCACCTGAC
pSR_check_R	Reverse check pSR insertion	CGACAAGTTGCTGCGATTCT
dsdC1_insert_pSR_F	Forward to clone the *dsdCX1* intergenic region into pSR with EcoRI RE	CGCGAATTCCGTGCTCACAACCCAGATTT
dsdC1_insert_pSR_R	Reverse to clone the *dsdCX1* intergenic region into pSR with HindIII RE	GCGAAGCTTCCTGGCTGCCACTTCAAAAG
neuO_qPCR_F	Forward for *neuO* RT-qPCR	TCGGTAGAAGAACGACAATAGGT
neuO_qPCR_R	Reverse for *neuO* RT-qPCR	ATGCAGTCATGGCCAATTGT
neuO_Red_F	Forward for *neuO* lambda red mutagenesis in CE10	TTTTAATGACAAGAAAAGTAACTAGTGG TAAAATAACGTAGGATACTAACGTGTAGGCTGGAGCTGCTTC
neuO_Red_R	Reverse for *neuO* lambda red mutagenesis in CE10	TTTATTACCTTATATTAATATTGGTAATATGTCTG CATGATGTTTTATATCATATGAATATCCTCCTTAG
neuO_184_F	Check forward for *neuO* lambda red mutagenesis in CE10	TGAAGTCAGCCCCATACGATGCCGCTGACTGATAATGACG
neuO_184_R	Check reverse for *neuO* lambda red mutagenesis in CE10	CAATCCATGCCAACCCGTTCCATTACCGCGGTTAATGCCC
pACYC-184_gibson_F	Forward for pACYC-184 linearisation	TGAATGGAAGCCGGCGGC

pACYC-184_gibson_R	Reverse for pACYC-184 linearisation	CATACACGGTGCCTGACTGC
pACYC_neuO_gibson_F	Forward for *neuO* for gibson assembly	GCAGTCAGGCACCGTGTATGTTATTGCGTGAGCTTCGC
neuO_gapAp_gibson_R	Reverse for *neuO* for gibson assembly	GGTGGAATATATGTCAAGACTCAAGACTCAAG
gapAp_neuO_gibson_F	Forward for *gapA* promoter for gibson assembly	GTCTTGACATATATTCCACCAGCTATTTG
gapAp_pACYC_R	Reverse for *gapA* promoter for gibson assembly	GTGCCGCCGGCTTCCATTCACGTAATTGCC CTTTAAAATTC
dsdC1_insert_pACYC_F	Forward for cloning CE10 dsdC1 with *Hind*III	CCCAAGCTTCAACGCAGGCTGACAAAC
dsdC1_insert_pACYC_R	Forward for cloning CE10 dsdC1 with *BamH*I	CCCGGATCCGGAACCAGATGATTTAATG
dsdC2_insert_pACYC_F	Forward for cloning CE10 dsdC2 with *Hind*III	CCCAAGCTTTTGCCTTTCACATGAGCT
dsdC2_insert_pACYC_R	Forward for cloning CE10 dsdC2 with *BamH*I	CCCGGATCCTCACTGATACTGACAAGA

Supplementary references

1. K. A. Datsenko, B. L. Wanner, One-step inactivation of chromosomal genes in Escherichia coli K-12 using PCR products. *Proc. Natl. Acad. Sci. U. S. A.* **97** (2000).
2. D. J. Lee, *et al.*, Gene doctoring: A method for recombineering in laboratory and pathogenic Escherichia coli strains. *BMC Microbiol.* **9** (2009).
3. J. P. R. Connolly, N. O'Boyle, N. C. A. Turner, D. F. Browning, A. J. Roe, Distinct intraspecies virulence mechanisms regulated by a conserved transcription factor. *Proc. Natl. Acad. Sci. U. S. A.* **116**, 19695–19704 (2019).
4. A. Zaslaver, *et al.*, A comprehensive library of fluorescent transcriptional reporters for Escherichia coli. *Nat. Methods* **3** (2006).
5. F. Strino, M. Lappe, Identifying peaks in *-seq data using shape information. *BMC Bioinformatics* **17** (2016).
6. M. D. Robinson, D. J. McCarthy, G. K. Smyth, edgeR: A Bioconductor package for differential expression analysis of digital gene expression data. *Bioinformatics* **26** (2009).
7. K. J. Livak, T. D. Schmittgen, Analysis of relative gene expression data using real-time quantitative PCR and the 2-ΔΔCT method. *Methods* **25** (2001).
